# DNA methylases for site-selective inhibition of type IIS restriction enzyme activity

**DOI:** 10.1007/s00253-024-13015-7

**Published:** 2024-01-25

**Authors:** Carol N. Flores-Fernández, Da Lin, Katherine Robins, Chris A. O’Callaghan

**Affiliations:** 1 Wellcome Trust Centre for Human Genetics, Nuffield Department of Medicine, University of Oxford, Roosevelt Drive, Oxford, OX3 7BN UK; 2 Current address: Triple Helix Biotechnology Ltd, Moneta Building (B280), Babraham Research Campus, Babraham, Cambridge, CB22 3AT UK; 3 Current address: Complete Regulatory, 19-20 King Edward Street, Macclesfield, SK10 1AQ UK

**Keywords:** Non-switchable methylases, Switch methylases, DNA methylation, Type IIS restriction enzymes, Recombinant methylases, Blocking endonuclease activity

## Abstract

**Abstract:**

DNA methylases of the restriction-modifications (R-M) systems are promising enzymes for the development of novel molecular and synthetic biology tools. Their use *in vitro* enables the deployment of independent and controlled catalytic reactions. This work aimed to produce recombinant DNA methylases belonging to the R-M systems, capable of *in vitro* inhibition of the type IIS restriction enzymes *Bsa*I, *Bpi*I, or *Lgu*I. Non-switchable methylases are those whose recognition sequences fully overlap the recognition sequences of their associated endonuclease. In switch methylases, the methylase and endonuclease recognition sequences only partially overlap, allowing sequence engineering to alter methylation without altering restriction. In this work, ten methylases from type I and II R-M systems were selected for cloning and expression in *E. coli* strains tolerant to methylation. Isopropyl β-D-1-thiogalactopyranoside (IPTG) concentrations and post-induction temperatures were tested to optimize the soluble methylases expression, which was achieved with 0.5 mM IPTG at 20 °C. The C-terminal His6-Tag versions showed better expression than the N-terminal tagged versions. DNA methylation was analyzed using purified methylases and custom test plasmids which, after the methylation reactions, were digested using the corresponding associated type IIS endonuclease. The non-switchable methylases M2.*Eco*31I, M2.*Bsa*I, M2.*Hpy*AII, and M1.*Mbo*II along with the switch methylases M.*Osp*807II and M2.*Nme*MC58II showed the best activity for site-selective inhibition of type IIS restriction enzyme activity. This work demonstrates that our recombinant methylases were able to block the activity of type IIS endonucleases *in vitro*, allowing them to be developed as valuable tools in synthetic biology and DNA assembly techniques.

**Key points:**

*• Non-switchable methylases always inhibit the relevant type IIS endonuclease activity*

*• Switch methylases inhibit the relevant type IIS endonuclease activity depending on the sequence engineering of their recognition site*

*• Recombinant non-switchable and switch methylases were active in vitro and can be deployed as tools in synthetic biology and DNA assembly*

**Graphical Abstract:**

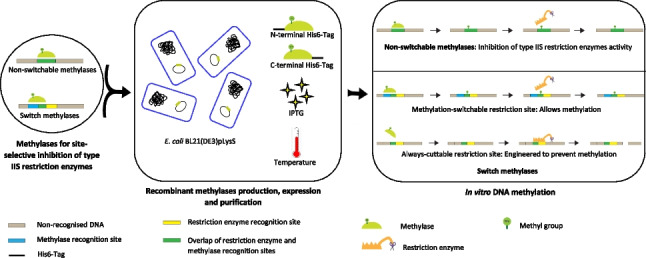

**Supplementary Information:**

The online version contains supplementary material available at 10.1007/s00253-024-13015-7.

## Introduction

DNA methylases are enzymes responsible for introducing methyl groups into DNA and have high potential in the development of tools for molecular and synthetic biology, genetic engineering, and epigenetics (Galbraith and Snuderl 2022; Lin and O’Callaghan 2018; Matsumura 2022). Bacteria produce methylases as solitary (orphan) enzymes, or as part of restriction-modification (R-M) systems in which they are associated with a restriction enzyme (Adhikari and Curtis 2016; Casadesús and Sánchez-Romero 2022). The function of a methylase in an R-M system is to protect the bacterial genomes from self-digestion by the associated restriction enzyme. Thereby, when a base within the recognition sequence of the restriction enzyme is methylated, the endonuclease is not able to digest the target DNA (Adhikari and Curtis 2016; Anton and Roberts 2021; Wilkowska et al. 2020). Three main types of R-M have been described. Types I and III are formed by multisubunit enzymes which have methylation and restriction activities in different subunits of the same enzyme. Type I comprises the S, M, and R subunits responsible for DNA specificity, methylation, and restriction; respectively. Type III consists of the Mod subunit which is responsible for DNA specificity and methylation, and the Res subunit which carries out the restriction. In contrast, type II consists of two separate enzymes that act independently for methylation and restriction. Necessarily, each one of these two enzymes has a specificity function. Type II are the most common and studied R-M systems (Casadesús and Sánchez-Romero 2022; Chen et al. 2021; Ge and Qiu 2022; Gulati et al. 2023; Loenen et al. 2014; Tock and Dryden 2005).

**Fig. 1:**
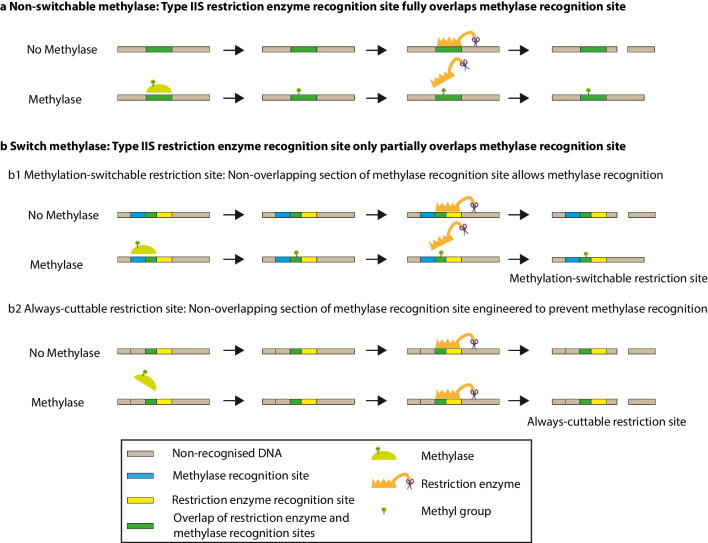
Schematic representation of **a** non-switchable and **b** switch methylase action. With switch methylases, the section of the methylase recognition site that does not overlap with the restriction enzyme recognition site can either contribute to methylase recognition allowing methylation to occur in a methylation-switchable site (b1) or can be engineered to prevent methylase recognition and so prevent methylation in an always-cuttable site (b2)

A number of methylases and type IIS restriction enzymes have been described (Roberts et al. 2023). Methylases can be used for different purposes depending on the relationship between their recognition site and activity with the recognition site for the relevant type IIS restriction enzyme. The recognition site for a type IIS restriction enzyme may fully or partially overlap with the recognition site for a methylase (Fig. 1). If the two sites fully overlap, such that the restriction enzyme recognition site always includes the methylase recognition site, then in the presence of the methylase, the restriction enzyme recognition site will always be methylated and so blocked. We term such methylases non-switchable methylases. The situation in which the methylase recognition site and the restriction enzyme recognition site only partially overlap is of particular interest and utility. In this situation, it is possible to alter the non-overlapping part of the methylase recognition site such that the methylase no longer recognizes this site, while leaving the restriction enzyme recognition site intact. In this way, the activity of the methylase at this restriction enzyme recognition site is “switched” off and we, therefore, term such methylases switch methylases. For these switch methylases, we term restriction enzyme recognition sites that have been altered to prevent methylase activity “always-cuttable” restriction sites. We term restriction enzyme recognition sites that allow methylase activity and so blockade “methylation-switchable” restriction sites term (Fig. 1 and Figure S1). Combinations of always-cuttable and methylation-switchable restriction sites offer particular value in DNA assembly (Lin and O’Callaghan 2018, 2020).

Studies demonstrating the separate applications of DNA methylases and type IIS restriction enzymes have been reported. DNA methylases have been widely used in epigenetics where different strategies for improving the specificity of targeted DNA methylation have been tested (Ślaska-Kiss et al. 2021). In addition, Weber et al. (2011) developed a modular cloning system for the assembly of multigene constructs using type IIS restriction enzymes. Meanwhile, the use of methylases and type IIS restriction enzymes together has been exploited for the development of novel molecular techniques allowing the assembly of multiple and large DNA fragments in a one-pot reaction (Lin and O’Callaghan 2018, 2020). However, most of these studies have been carried out using *in vivo* methylation. The alternative approach of using purified recombinant enzymes for *in vitro* methylation offers considerable advantages. For instance, the catalytic activity is not dependent on, or limited by, cell growth, survival, or function, which is especially important given the potentially toxic consequences of DNA methylation to the host organism. In addition, the *in vitro* approach greatly facilitates the testing and comparison of two or more enzymes of different enzyme concentrations and generally provides much greater control of the reaction conditions (Golynskiy et al. 2013; Sanchez and Demain 2012).

The aim of this work was to obtain highly active purified recombinant methylases and to test their *in vitro* capacity to block the action of the relevant type IIS restriction enzyme at specific sites. To achieve this, candidate methylases were selected for molecular cloning and expression. After purification, their activity was tested on different plasmids, some of which were especially constructed to contain sites where the activity of the restriction enzyme would be selectively blocked by methylase activity. The results of this work demonstrate that our recombinant methylases displayed excellent *in vitro* activity and, along with their associated type IIS restriction enzymes, could be applied in the development of synthetic biology and DNA assembly techniques.

## Materials and methods

### Bacterial strains, plasmids, enzymes, and chemicals

Unless otherwise stated, bacterial strains, restriction enzymes, and other molecular biology reagents were purchased from New England Biolabs (Hitchin, UK), while plasmids were purchased from Novagen and chemicals from Sigma-Aldrich, Merck KGaA (Darmstadt, Germany).

### Molecular cloning of methylases

A total of 10 methylases (Table S1) whose recognition sequences fully or partially overlap with the recognition sequences of the type IIS restriction enzymes *Bsa*I, *Bpi*I, and *Lgu*I were retrieved from REBASE (http://rebase.neb.com/rebase/index.html). The gene sequences encoding these enzymes were codon-optimized for cloning and expression. All the synthetic sequences are shown in the Supplementary Information. Primers, restriction enzymes, and plasmids used for PCR, cloning, and expression of the methylase genes are described in Table 1. For M2.*Eco*31I and M2.*Bsa*I, truncated versions for which the second ATG was used as the start codon were also cloned as M2.*Eco*31I_2 and M2.*Bsa*I_2, respectively. All the methylase genes were cloned separately with an N- and C-terminal His6-Tag. PCR reactions were carried out using Q5 High-Fidelity DNA Polymerase in 25 μL reactions under the following conditions: 98 °C for 30 s followed by 30 cycles of denaturation at 98 °C for 10 s, annealing according to Table S2 for 20 s and extension at 72 °C for 1 min; then a final extension at 72 °C for 2 min (MJ Research – PTC-225 Peltier Thermal Cycler PCR 96 well Tetrad 4 block). Following electrophoresis, the correctly sized PCR products were purified from agarose gels using QIAquick Gel Extraction Kit (Qiagen, Manchester, UK) and ligated into the plasmids using T4 DNA ligase. The ligated mixtures were transformed into NEB^®^ 10-beta Competent *E. coli*, and the recombinant colonies were screened by colony PCR using BIOTAQ™ DNA Polymerase (Bioline, London, UK). Then, the recombinant plasmids were extracted using QIAprep^®^ Spin Miniprep Kit and sent for sequencing (Source BioScience Genomics). Finally, the plasmids were transformed into the expression host *E. coli* BL21(DE3)pLysS (Novagen, Merck KGaA).

**Table 1:** Primers, restriction enzymes, and plasmids used for PCR, cloning, and expression of methylases

Methylase	POC number - Code	Plasmid^a^	His6-tag Terminal	Forward primer	Restriction enzyme	Reverse primer	Restriction enzyme	Gene size (bp)	Protein size (aa)	Protein MW^c^ (kDa)
M2.*Eco*31I	POC1463 - KPL01	pET15b	N	CO9302	*Nde*I	CO9304	*Bam*HI	1191	396	46.96 46.31
	POC1464 - KPL02	pET30b	C	CO9302	*Nde*I	CO9303	*Hin*dIII			
M2.*Eco*31I_2	POC1465 - KPL03	pET15b	N	CO9301	*Nde*I	CO9304	*Bam*HI	1170	389	46.13 45.49
	POC1466 - KPL04	pET30b	C	CO9301	*Nde*I	CO9303	*Hind*III			
M2.*Bsa*I	POC1467 - KPL05	pET15b	N	CO9305	*Nde*I	CO9308	*Bam*HI	1188	395	46.51 45.87
	POC1468 - KPL06	pET30b	C	CO9305	*Nde*I	CO9307	*Hin*dIII			
M2.*Bsa*I_2	POC1469 - KPL07	pET15b	N	CO9306	*Nde*I	CO9308	*Bam*HI	1146	381	44.96 44.32
	POC1470 - KPL08	pET30b	C	CO9306	*Nde*I	CO9307	*Hin*dIII			
M.*Osp*807II^b^	POC1471 - KPL09	pET15b	N	CO9309	*Nde*I	CO9311	*Bam*HI	1068	355	41.85 40.75
	POC1472 - KPL10	pET30b	C	CO9309	*Nde*I	CO9310	*Xho*I			
M2.*Nme*MC58II^b^	POC1473 - KPL11	pET15b	N	CO9312	*Nde*I	CO9314	*Bam*HI	651	216	27.56 26.92
	POC1474 - KPL12	pET30b	C	CO9312	*Nde*I	CO9313	*Hin*dIII			
M.*Sen*0738I^bd^	POC1475 - KPL13	pET15b	N	CO9315	*Nde*I	CO9317	*Xho*I	1470	489	57.75 56.65
	POC1476 - KPL14	pET30b	C	CO9315	*Nde*I	CO9316	*Xho*I			
S.*Sen*0738I^d^	POC1477 - KPL15	pET15b	N	CO9318	*Nde*I	CO9320	*Bam*HI	1779	592	68.85 67.75
	POC1478 - KPL16	pET30b	C	CO9318	*Nde*I	CO9319	*Xho*I			
M1.*Eco*31I	POC1479 - KPL17	pET28a	N	CO9323	*Nhe*I	CO9324	*Bam*HI	1641	546	64.58 63.65
	POC1480 - KPL18	pET28a	C	CO9325	*Nco*I	CO9326	*Hin*dIII			
M.*Xmn*I^b^	POC1481 - KPL19	pET28a	N	CO9331	*Nhe*I	CO9332	*Hin*dIII	1863	620	71.10 70.17
	POC1482 - KPL20	pET28a	C	CO9333	*Nco*I	CO9334	*Hin*dIII			
M1.*Hpy*AII	POC1483 - KPL21	pET28a	N	CO9384	*Nde*I	CO9385	*Bam*HI	783	260	32.63 31.75
	POC1484 - KPL22	pET30b	C	CO9384	*Nde*I	CO9386	*Not*I			
M2.*Hpy*AII	POC1485 - KPL23	pET28a	N	CO9452	*Nde*I	CO9453	*Not*I	864	287	35.68 34.79
	POC1486 - KPL24	pET30b	C	CO9339	*Nde*I	CO9341	*Not*I			
M1.*Mbo*II	POC1487 - KPL25	pET28a	N	CO9454	*Nde*I	CO9455	*Not*I	795	264	32.79
	POC1488 - KPL26	pET30b	C	CO9342	*Nde*I	CO9344	*Hin*dIII			32.14

^a^pET15b: ampicillin^R^, pET30b: kanamycin^R^, and pET28a: kanamycin^R^; ^b^switch methylase; ^c^molecular weight including His6-tag; and aa: amino acids. ^d^The M.Sen0738I M subunit forms a complex with the S.Sen0738I S subunit (M_2_S) for methylation. *MW* molecular weight

### Expression of the recombinant methylases

*E. coli* BL21(DE3)pLysS harboring the recombinant plasmids were grown in 10 mL of LB broth containing either 0.1 g/L ampicillin or 0.05 g/L kanamycin with 0.05 g/L chloramphenicol and incubated at 37 °C overnight. The overnight starter cultures were used to inoculate 150 mL of the same medium without chloramphenicol. Cultivation was carried out in 1-L baffled shake flasks at 37 °C and 230 rpm in a shaker incubator (New Brunswick™ Innova^®^ 44/44R Incubator Shakers, Eppendorf, Stevenage UK) until the absorbance at 600 nm reached 0.3–0.5. At this point, the cells were induced by the addition of different isopropyl β-D-1-thiogalactopyranoside (IPTG) concentrations (0.005, 0.05, 0.5, and 1 mM) and incubated at 37 °C and 230 rpm for a further 5 h. In addition, incubation temperatures of 20, 30, and 37 °C after 0.5 mM IPTG induction were tested for 24, 18, and 5 h, respectively. The cells were harvested by centrifugation (4000×g at 4 °C for 20 min) and stored at −20 °C until analysis. For testing the total protein expression, the cell pellets were resuspended in Sample Buffer Laemmli 2X Concentrate, heated at 95 °C for 10 min, and analyzed by SDS-PAGE. For testing the soluble protein expression, the cell pellets were re-suspended in 50 mM sodium phosphate buffer, pH 7.5, and disrupted by sonication (Ultrasonic processors, Vibra-Cell™ VCX 500) with 20 cycles of 10 s ON and 50 s OFF at 35% amplitude. Subsequently, the cell suspension was centrifuged (17,200×g at 4 °C for 30 min) and the clarified cell lysate was recovered and kept at 4 °C for protein quantification and SDS-PAGE analysis. Protein concentration was determined using Pierce™ BCA Protein Assay Kit (Thermo Fisher Scientific, Loughborough, UK) following the microplate procedure. SDS-PAGE was performed using 10% Mini-PROTEAN^®^ TGX™ Precast Protein Gels (Bio-Rad, Watford, UK), and the protein bands were visualized with InstantBlue^®^ Coomassie Protein Stain (Abcam, Cambridge, UK). Amersham™ ECL™ Rainbow™ Marker - Full range RPN800E, 12 to 225 kDa (Cytiva, Amersham, UK), and PageRuler™ Plus Prestained Protein Ladder, 10 to 250 kDa (Thermo Fisher Scientific), were used as molecular weight markers.

### Purification of the recombinant methylases

The methylases were purified by gravity-flow affinity chromatography using a His-Tag Ni-affinity resin (Ni-NTA Agarose, Thermo Fisher Scientific Inc., UK). Buffers containing 10, 50, and 400 mM imidazole were used as binding, washing, and elution buffer, respectively. These buffers were prepared in 50 mM sodium phosphate buffer containing 300 mM NaCl and 10% glycerol, pH 7.5. The purified enzymes were desalted using PD-10 Desalting Columns (Cytiva). The desalted purified enzymes were stored at −80 °C for further assays. Plasmids encoding the enzymes are available from the Addgene repository.

### Methylases activity assays

All the methylase activity assays were carried out using purified enzymes. The activity of the *Bsa*I and *Bpi*I-associated non-switchable methylases was tested using pET15b. *Lgu*I-associated non-switchable methylases were not included in this study. The methylation reactions were carried out by mixing 2 μL of 10X methylase buffer (0.5 M Tris-HCl, 0.1 M EDTA, and 50 mM DTT, pH 7.5), 1 μL of 3200 μM S-adenosylmethionine (SAM), 200 ng of pET15b, and 1 μL of each methylase in a 20 μL reaction. The mixtures were incubated at 37 °C for 1 h and heat-inactivated at 80 °C for 20 min. For *Bsa*I-associated methylases, the restriction reactions were performed by adding 3 μL of the corresponding buffer, 4 μL of 50 mM MgCl_2_, and 0.5 μL of *Bsa*I and *Apa*I to each methylation reaction. For *Bpi*I-associated methylases, the restriction reactions were carried out by adding 0.5 μL of *Bpi*I and *Bsa*I. The final volume of the restriction reactions was 30 μL and they were incubated at 37 °C for 1 h. Negative controls without the addition of the methylase were used. The methylase activity was analyzed by 1% agarose gel electrophoresis using Quick-Load^®^ 1 kb Extend DNA Ladder, 0.5 to 48.5 kb as molecular weight marker.

The activity of *Bsa*I, *Bpi*I, and *Lgu*I-associated switch methylases was tested using POC1399, POC1400, and POC1401 plasmids, respectively. These plasmids were generated by synthesis using the MoClo pL1R-1 plasmid vector backbone as a base (Weber et al. 2011). The methylation and restriction reactions were carried out as described above using the corresponding associated restriction enzyme.

In addition, the effect of pH on methylase activity was assayed using the methylase buffer at pH values of 6.9, 7.5, 7.9, 8.4, and 8.8 and following the procedure described above.

## Results

### Molecular cloning of methylases

To produce recombinant methylases with the capacity for *in vitro* methylation and so blockade of the recognition sites of the type IIS restriction enzymes *Bsa*I, *Bpi*I, and *Lgu*I, 10 methylases were selected (Table 2). All the genes encoding these methylases were amplified by PCR (Figures S2–S6) and successfully cloned in the plasmids described in Table 1, except the N-terminal His6-Tag versions of M2.*Hpy*AII and M1.*Mbo*II (KPL23 and KPL25, respectively). In these two cases, the recombinant plasmids containing the methylase genes could not be confirmed by colony PCR or sequencing.

**Table 2:** Methylases recognition sequence and methylated bases, methylases-associated endonucleases, and the resulting methylated products

Methylase	Methylase recognition sequence^b^ and methylated base^c^	Associated type IIS endonuclease	Type IIS endonuclease recognition sequence^d^ and restriction site^e^	Plasmid for the activity test	Methylated product^f^
M2.*Eco*31I (KPL01 – KPL04)	5′... GGT**C**TC ... 3′ 3′... CCAGAG... 5′	*Bsa*I	5′... GGTCTC(N)_1_^**▼**^… 3′ 3′...CCAGAG(N)_5▲_ ... 5′	pET15b	5′... GGT**C**TC(N)_1_^**▼**^… 3′ 3′...CCAGAG(N)_5▲_ ... 5′
M1.*Eco*31I (KPL17 – KPL18)	5′... GGTCTC ... 3′ 3′... CC**A**GAG... 5′				5′... GGTCTC(N)^1▼^… 3′ 3′...CC**A**GAG(N)_5▲_ ... 5′
M2.*Bsa*I (KPL05 – KPL08)	5′... GGTCTC ... 3′ 5′... CCAGAG ... 3′ 5-methylcytosine top strand (base undetermined)				5′... GGTCTC(N)_1_^**▼**^… 3′ 3′...CCAGAG(N)_5▲_ ... 5′ 5-methylcytosine top strand (base undetermined)
M1.*Hpy*AII (KPL21 – KPL22)	5′... GAAG**A** ... 3′ 3′... CTTCT ... 5′	*Bpi*I	5′...GAAGAC(N)_2_^**▼**^… 3′ 3′... CTTCTG(N)_6▲_ ... 5′	pET15b	5′...GAAG**A**C(N)^2▼^… 3′ 3′... CTTCTG(N)_6▲_ ... 5′
M2.*Hpy*AII (KPL23 – KPL24)	5′... GAAGA ... 3′ 3′... CTT**C**T ... 5′				5′...GAAGAC(N)^2▼^… 3′ 3′... CTT**C**TG(N)_6▲_ ... 5′
M1.*Mbo*II (KPL25 – KPL26)	5′... GAAG**A** ... 3′ 3′... CTTCT ... 5′				5′...GAAG**A**C(N)_2_^**▼**^… 3′ 3′... CTTCTG(N)_6▲_ ... 5′
M.*Osp*807II^a^ (KPL09 – KPL10)	5′... G**A**CNNNGTC ... 3′ 3′... CTGNNNC**A**G ... 5′	*Bsa*I	5′... GGTCTC(N)_1_^**▼**^… 3′ 3′...CCAGAG(N)_5▲_ ... 5′	POC1399	5′... G**A**CNNGGTCTC(N)_1_^**▼**^ ... 3′ 3′... CTGNNCC**A**GAG(N)_5**▲**_ ... 5′
M.*Sen*0738I^a^ and S.Sen0738I (KPL13 – KPL16)	5′... CC**A**GNNNNNNNNTCT ... 3′ 3′... GGTCNNNNNNNN**A**GA ... 5′		5′... GGTCTC(N)_1_^**▼**^… 3′ 3′...CCAGAG(N)_5▲_ ... 5′	-	5′... CC**A**GNNNNNNGGTCTC(N)_1_^▼^… 3′ 3′...GGTCNNNNNNCC**A**GAG(N)_5▲_ ... 5′
M2.*Nme*MC58II^a^ (KPL11 – KPL12)	5′... G**A**CGC ... 3′ 3′... CTGCG ... 5′	*Bpi*I	5′...GAAGAC(N)_2_^**▼**^… 3′ 3′... CTTCTG(N)_6▲_ ... 5′	POC1400	5′...GAAG**A**CGC ^▼^… 3′ 3′... CTTCTGCGN_4▲_ ... 5′
M.*Xmn*I^a^ (KPL19 – KPL20)	5′... G**A**ANNNNTTC ... 3′ 3′... CTTNNNNA**A**G ... 5′	*Lgu*I	5′... GCTCTTC (N)_1_^**▼**^… 3′ 3′...CGAGAAG(N)_4▲_ ... 5′	POC1401	5′... G**A**AGCTCTTC (N)_1_^**▼**^… 3′ 3′...CTTCGAGA**A**G(N)_4▲_ ... 5′

^a^Switch methylases, ^b^the methylase recognition sequence is in red, ^c^the methylated base is in bold and underlined, ^d^the endonuclease recognition sequence is highlighted in light blue, ^e^the endonuclease restriction site is marked with ^▼^_▲_, ^f^the methylated product shows the methylase recognition sequence and the methylated base as well as the endonuclease recognition sequence and its restriction site

### Expression and purification of recombinant methylases

**Fig. 2:**
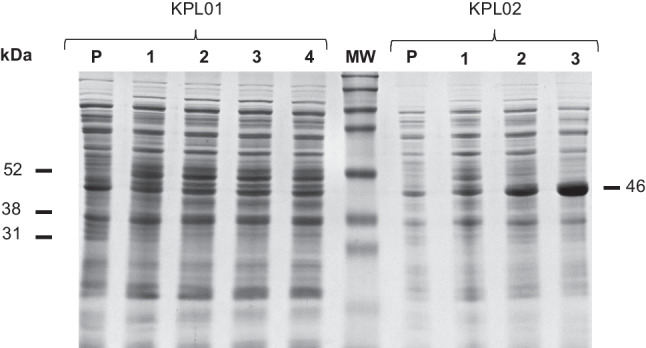
SDS-PAGE showing the expression of M2.*Eco*31I (KPL01 and KPL02) at different IPTG concentrations. Lanes: P, pre-induction; 1, 0.005 mM; 2, 0.05 mM; 3, 0.5 mM; and 4, 1 mM. MW, molecular weight marker (Amersham™ ECL™ Rainbow™ Marker - Full range, Full Range, Cytiva RPN800E, 12 to 225 kDa)

Different IPTG concentrations were tested to improve the expression of the recombinant methylase proteins in *E. coli* BL21(DE3)pLysS. The C-terminal His6-Tag versions of the non-switchable methylases M2.*Eco*31I (KPL02), M2.*Eco*31I_2 (KPL04), M2.*Bsa*I (KPL06), M2.*Bsa*I_2 (KPL08), M1.*Hpy*AII (KPL22), M2.*Hpy*AII (KPL24), and M1.*Mbo*II (KPL26) showed better expression than their N-terminal His6-Tag, with 0.5 mM being the optimum IPTG concentration (Fig. 2 and Figures S7, S8, S9, S16, and S17). In the case of the switch methylases M.*Osp*807II (KLP09 and KPL10), M2.*Nme*MC58II (KPL11 and KLP12), and M.*Xmn*I (KPL19 and KPL20), both N- and C-terminal His6-Tag versions exhibited good expression levels with an optimum IPTG concentration of 0.5 mM for both enzymes (Figures S10, S11, and S15). Subsequently, different temperatures were assayed to enhance the soluble protein expression. All the methylases described above were best expressed in their soluble form at 20 °C. However, lower cell density was observed at this temperature compared to 37 °C for M2.*Bsa*I_2 (KPL08), M2.*Nme*MC58II (KPL11), M.*Xmn*I (KPL19), and M2.*Hpy*AII (KPL24), so these enzymes were expressed at 37 °C. Neither the N- nor C-terminal His6-Tag versions of M.*Sen*0738I (KPL13 and KPL14), S.*Sen*0738I (KPL15 and KPL16), and M1.*Eco*31I (KPL17 and KPL18) were expressed as assayed by SDS-PAGE analysis (Figures S12, S13, and S14).

**Fig. 3:**
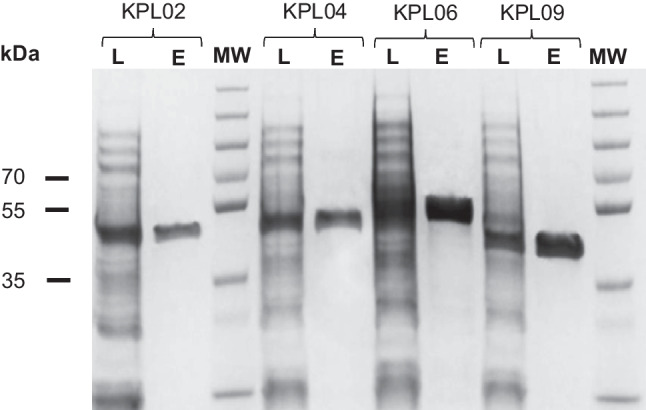
SDS-PAGE showing the purification by affinity chromatography of methylases M2.*Eco*31I (KPL02), M2.*Eco*31I_2 (KPL04), M2.*Bsa*I (KPL06), and M.*Osp*807II (KPL09). Lanes: L, clarified lysate; and E, eluted fraction. MW, molecular weight marker (PageRuler™ Plus Prestained Protein Ladder, Thermo Fisher Scientific 26619, 10 to 250 kDa)

All the methylases successfully expressed in their soluble form were purified by affinity chromatography with a protein recovery of around 99% based on protein quantification assays. The purification process was analyzed by SDS-PAGE where single bands were observed for the purified enzymes (Fig. 3). The methylases did not lose activity during the purification, and no significant differences in activity or purity were observed between batches.

### Analysis of DNA methylation

**Fig. 4:**
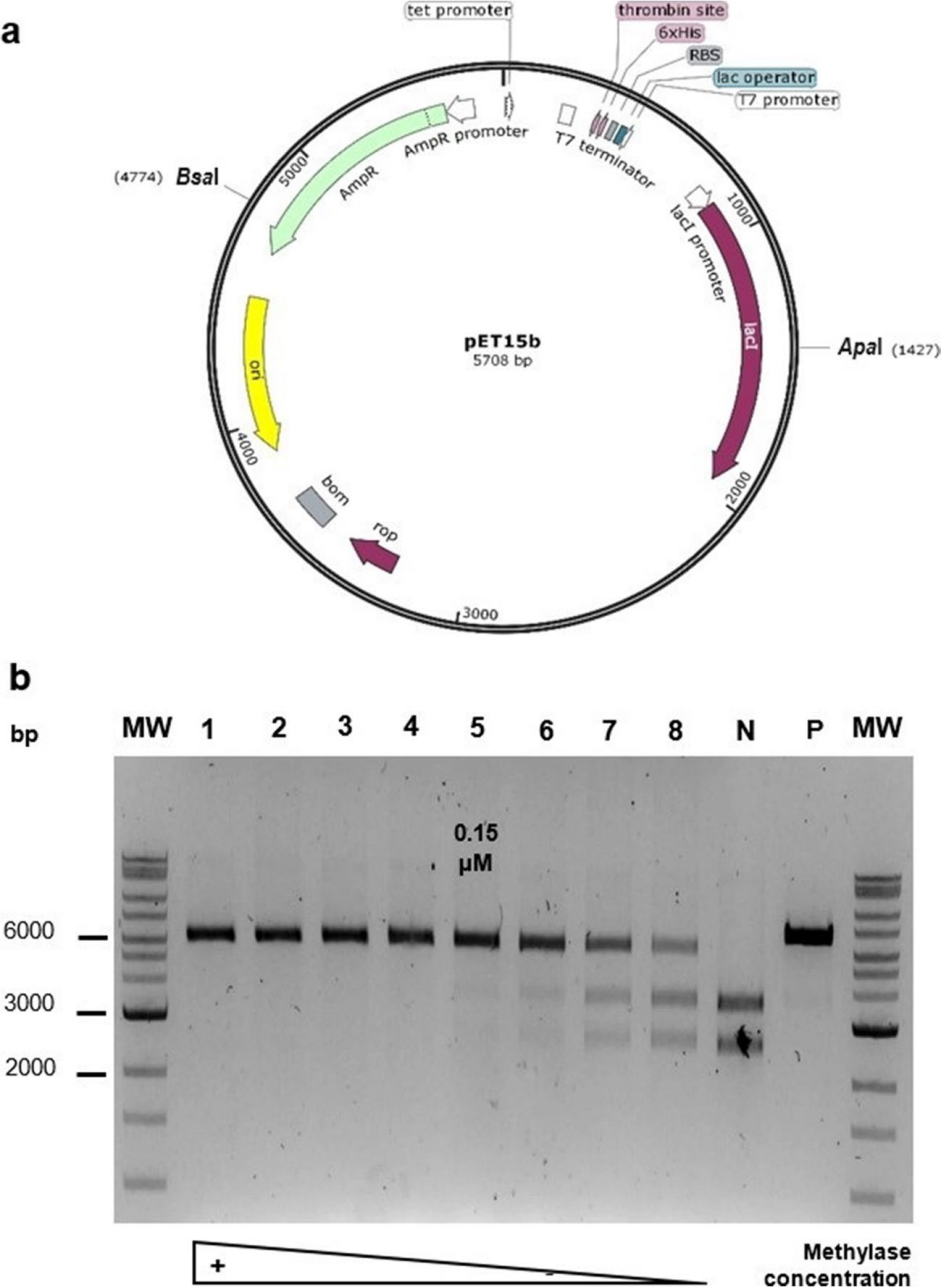
Activity of *Bsa*I-associated non-switchable methylases on pET15b. **a** pET15b showing the *Bsa*I and *Apa*I restriction sites for the activity test (Figure S18). The methylated plasmid will exhibit on the gel a band (5708 bp) corresponding to the linearized plasmid digested only by *Apa*I. The non-methylated plasmid will exhibit two bands (2361 and 3347 bp) as a result of the digestion by both enzymes. **b** Agarose gel electrophoresis (1%) showing the activity of M2.*Eco*31I (KPL02) at different concentrations. Lanes: 1 to 8, twofold serial dilutions from 2.4 to 0.019 μM; N, negative control (without methylase); and P, positive control (pET15b from a methylase expressing strain). MW, molecular weight marker (Quick-Load^®^ 1 kb Extend DNA Ladder, New England Biolabs, N3239S, 0.5 to 48.5 kb)

**Fig. 5:**
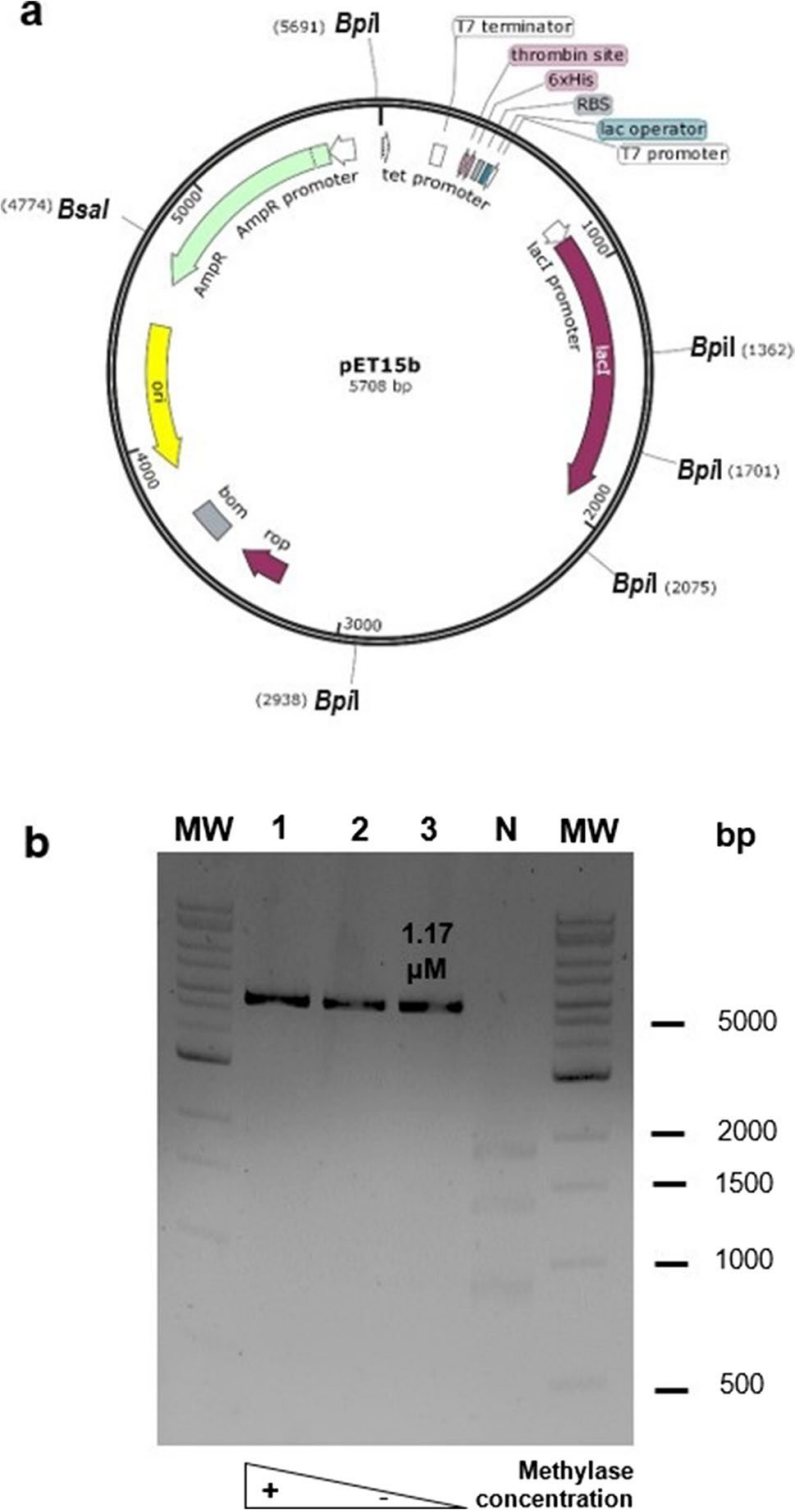
Activity of *Bpi*I-associated non-switchable methylases on pET15b. **a** pET15b showing the *Bpi*I and *Bsa*I restriction sites for the activity test (Figure S21). The methylated plasmid will exhibit on the gel a band (5708 bp) corresponding to the linearized plasmid digested only by *Bsa*I. The non-methylated plasmid will exhibit six bands (339, 374, 863, 917, 1379, and 1836 bp) as a result of the digestion by both enzymes. **b** Agarose gel electrophoresis (1%) showing the activity of M2.*Hpy*AII (KPL24) at different concentrations. Lanes: 1 to 3, 2-fold serial dilutions from 4.68 to 1.17 μM; and N, negative control (without methylase). MW, molecular weight marker (Quick-Load^®^ 1 kb Extend DNA Ladder, New England Biolabs, N3239S, 0.5 to 48.5 kb). Bands less than 500 bp are not observed in the gel

Detailed information about the methylases, their associated type IIS endonucleases, and their activity is described in Table 2. The activity of the *Bsa*I and *Bpi*I-associated non-switchable methylases was tested using pET15b, which has one restriction site each for *Bsa*I and *Apa*I as well as five restriction sites for *Bpi*I. The recognition sequences of the non-switchable methylases fully overlap with those of their associated endonucleases, so all the restriction sites will be methylated, and the endonuclease activity will be blocked (Figures S18 and S21). In the case of the *Bsa*I-associated methylases, the activity was evidenced by the restriction of the plasmid with *Bsa*I and *Apa*I after the methylation reaction. If methylation occurs, a band corresponding to the linearized plasmid cut by *Apa*I would be observed in the gel. In the absence of methylation, two bands of around 2361 and 3347 bp will be observed as a result of digestion by both *Apa*I and *Bsa*I. Enzyme titration assays showed that M2.*Eco*31I (KPL02) generated fully methylated plasmids from 0.15 μM (Fig. 4) and its truncated version M2.*Eco*31I_2 (KPL04) from 0.014 μM (Figure S19), demonstrating that the truncated version was more active than the full-length enzyme. Conversely, M2.*Bsa*I (KPL06) exhibited good activity from 1.6 μM (Figure S20), while its truncated version M2.*Bsa*I_2 (KPL08) did not have demonstratable activity. For *Bpi*I-associated methylases, the activity was evidenced by the restriction of the plasmid with *Bpi*I and *Bsa*I following the methylation reaction. If methylation occurs, a band corresponding to the plasmid linearized by *Bsa*I would be observed in the gel. In the absence of methylation, six bands of around 339, 374, 863, 917, 1379, and 1836 bp will be observed as a result of digestion by both *Bpi*I and *Bsa*I. At the tested concentrations, M1.*Hpy*AII (KPL22) demonstrated low activity generating partially methylated plasmids (Figure S22), while M2.*Hpy*AII (KPL24) and M1.*Mbo*II (KPL26) demonstrated good activity producing full methylation (Fig. 5 and Figure S23, respectively).

**Fig. 6:**
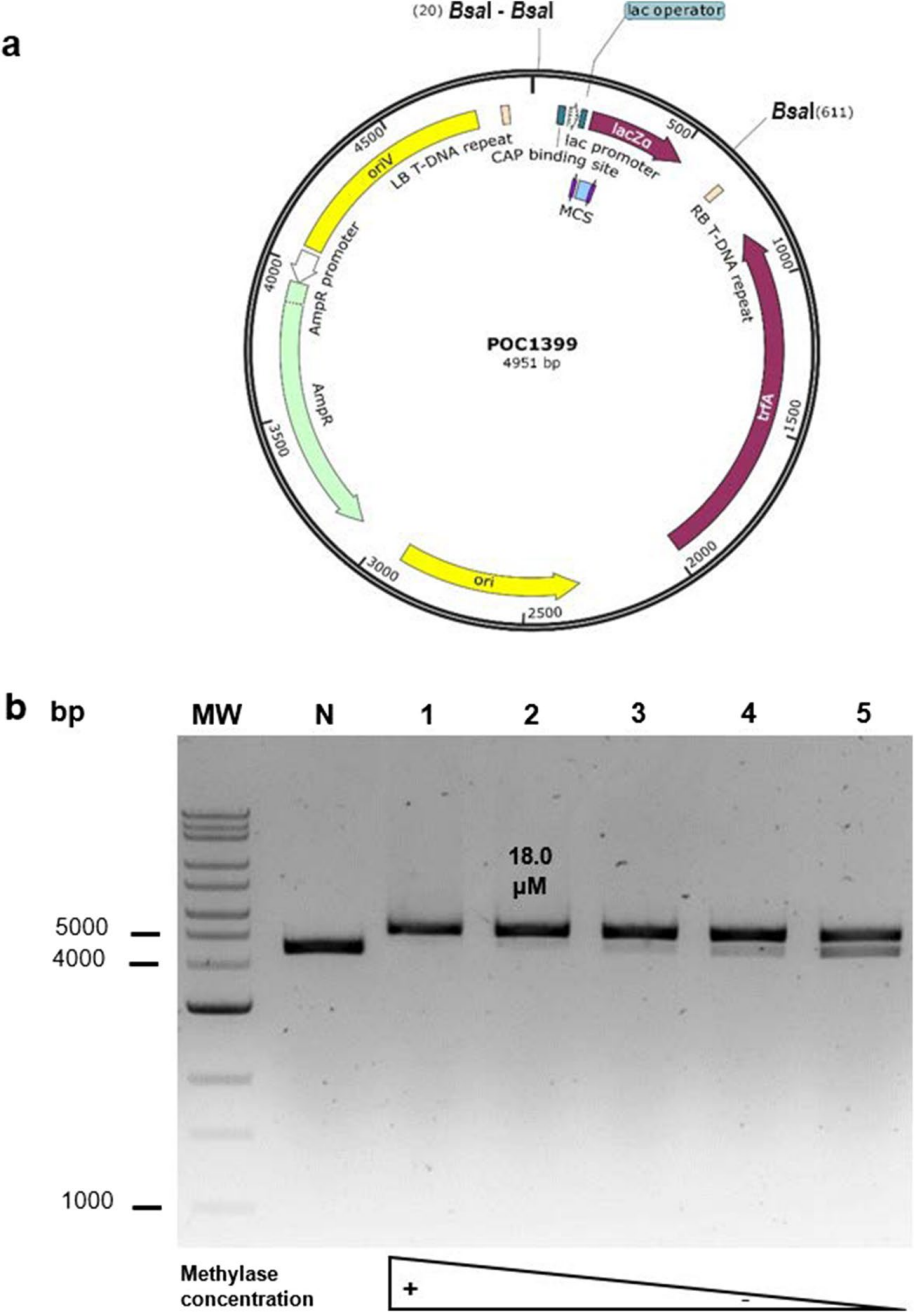
Activity of *Bsa*I-associated switch methylases on POC1399. **a** POC1399 showing the *Bsa*I restriction sites for the methylase activity test (Figure S24). The methylated plasmid will be cut by *Bsa*I at the always-cuttable site at position 611 resulting in a single band (4951 bp) corresponding to the linearized plasmid. In contrast, the non-methylated plasmid will be cut at the always-cuttable site at position 611 and at the methylation-switchable site at position 20 resulting in two bands (591 and 4360 bp). **b** Agarose gel electrophoresis (1%) showing the activity of M.*Osp*807II (KPL09) at different protein concentrations. Lanes: MW, molecular weight marker (Quick-Load® 1 kb Extend DNA Ladder, New England Biolabs, N3239S, 0.5 to 48.5 kb); N, negative control (without methylase); and 1 to 5 correspond to 24, 18, 12, 6, and 2.4 μM of methylase, respectively. Bands less than 1000 bp are not observed in the gel

Plasmids POC1399, POC1400, and POC1401 were constructed to test the activity of the *Bsa*I, *Bpi*I, and *Lgu*I-associated switch methylases, respectively. Based on the recognition sequences and activity of the switch methylases and type IIS restriction enzymes, these plasmids were each designed to have one methylation-switchable and one always-cuttable restriction site for the respective enzyme. The methylation-switchable site incorporates both the recognition sequence of the methylase and that of the endonuclease; therefore, in the presence of the methylase, DNA methylation will occur, blocking the endonuclease activity. In contrast, the always-cuttable site only contains the recognition sequence of the endonuclease and not of the methylase; therefore, in the presence of the methylase, the DNA will not be methylated and so in the presence of the endonuclease, it will be digested (Fig. 1b and Figures S1b, S24, S25, and S27). The activity of the methylases was evidenced by the restriction of the plasmids either with *Bsa*I, *Bpi*I, or *Lgu*I after the methylation reactions. If methylation occurs, there should be a band corresponding to the linearized plasmid, digested only at the single always-cuttable site. In contrast, if no methylation occurs, there should be two bands of 4360 bp and around 600 bp arising from digestion at both the always-cuttable and methylation-switchable sites. The assays showed that fully methylated plasmids were produced by the *Bsa*I-associated M.*Osp*807II (KPL09) at concentrations of 18 μM and above (Fig. 6), and for the *Bpi*I-associated M2.*Nme*MC58II (KPL11) at concentrations of 0.58 μM and above (Figure S26). However, even at the lowest concentrations tested (2.4 μM for M.*Osp*807II and 0.07 μM for M2.*Nme*MC58II), the amount of methylated plasmid was higher than that of non-methylated plasmid. For these two enzymes, comparable activity was observed for both the N- and C-terminal His6-Tag versions. For the *Lgu*I-associated M.*Xmn*I (KPL19 and KPL20), both N- and C-terminal His6-Tag versions exhibited low activity resulting in only partially methylated plasmids at the tested concentrations (Figure S28). Finally, to optimize the activity of some of the methylases, different pH values were tested. The results showed that the enzymes exhibited their highest activity at the standard pH value of 7.5 (Figure S29).

## Discussion

Bacterial DNA methylases in combination with type IIS restriction endonucleases are useful enzymes for the development of different tools in molecular biology, synthetic biology, and genetic engineering. The study of methylases, their production as purified recombinant enzymes, and the analysis of *in vitro* DNA methylation provide further insights into their potential applications. Methylases selected for this study belong to type I and II R-M systems and use SAM as the methyl group donor (Ge and Qiu 2022; Tock and Dryden 2005). In this work, M.*Sen*0738I and S.*Sen*0738I are M and S subunits, respectively, from a type I R-M system, and must form a complex (M_2_S) to achieve DNA methylation (Anton and Roberts 2021; Gulati et al. 2023; Tock and Dryden 2005). The rest of our methylases belong to the type II R-M system, mainly to subtype IIS which recognizes asymmetrical sequences and methylates a single strand in the target sequence (Furmanek-Blaszk et al. 2009; Kumar et al. 2018, 2020; Madhusoodanan and Rao 2010; Zhu et al. 2004). DNA methylation typically occurs on adenine at the sixth position (N6-methyladenine: m6A) or on cytosine either at the fourth (N4-methylcytosine: m4C) or the fifth position (C5-methylcytosine: m5C) (Anton and Roberts 2021; Ge and Qiu 2022; Gulati et al. 2023; Jurkowska and Jeltsch 2022). In our study, we included methylases that can introduce methyl groups on either adenine or cytosine residues.

*E. coli* is the most widely used host for recombinant enzyme production due to its well-studied genetics, fast growth, easy and fast transformation, and capacity for using low-cost substrates (Chou 2007; Rosano and Ceccarelli 2014; Terpe 2006). Our methylases were cloned and expressed in *E. coli* strains. However, methylases are potentially toxic enzymes and might cause the death of the bacterial hosts used for enzyme expression (Furuta et al. 2022). Such toxicity could underlie the unsuccessful cloning of the N-terminal His6-Tag versions of M2.*Hpy*AII and M1.*Mbo*II, even in methylation tolerant NEB^®^ 10-beta Competent *E. coli* cells. *E. coli* BL21(DE3)pLysS, which is favored for toxic protein expression, was chosen as the expression host for all methylases. Despite this, M.*Sen*0738I and S.*Sen*0738I as well as M1.*Eco*31I were not efficiently expressed. All the methylases that were expressed demonstrated good soluble expression at 20 °C, which is likely to arise because lower temperatures favor protein folding and reduce inclusion body formation (Liu et al. 2013; Rosano and Ceccarelli 2014; Terpe 2006; Vera et al. 2007). The very low cell density observed in some methylase cultures might be attributed to the potential toxicity of the enzymes (Rosano and Ceccarelli 2014; Terpe 2006). The soluble expression of M.*Sen*0738I and S.*Sen*0738I as well as M1.*Eco*31I might be improved by modifying the culture conditions (medium composition, pH, temperature, and agitation), inducer concentration, affinity tag, and the expression host (Liu et al. 2013; Terpe 2006). Our results show that in most cases, there was better soluble expression of the C-terminal His6-Tag versions than of the N-terminal His6-Tag. This is consistent with studies showing that C-terminal tags can improve the solubility of some recombinant enzymes (Costa et al. 2014; Paraskevopoulou and Falcone 2018). The truncated version of M2.*Eco*31I which uses the second ATG as the start codon was more active than the full-length enzyme. This parallels the findings of Miura et al. (2022) who reported that for some methylases, N-terminal deletions enhanced their activity.

We tested the activity of our recombinant methylases in blocking the activity of an associated type IIS restriction enzyme. Based on the extent of overlap between the methylase recognition sequence and the restriction enzyme recognition site, we classified these enzymes as non-switchable or switch methylases. The recognition sequence of the non-switchable methylases fully overlaps with that of the associated endonuclease, so in the presence of the methylase, all the restriction enzyme recognition sites will be methylated and blocked from digestion. In the case of switch methylases, their recognition sequence only partially overlaps with that of the associated endonuclease, allowing the sites to be engineered such that the restriction enzyme recognition site is maintained, but the methylase recognition site is no longer active. In the presence of the methylase and the restriction enzyme, the natural methylation-switchable site will be methylated and protected from digestion, but the modified always-cuttable site will not be methylated and will be digested by the restriction enzyme (Fig. 1 and Figure S1). Thus, the non-switchable *Bsa*I-associated methylases M2.*Eco*31I, M2.*Eco*31I_2, and M2.*Bsa*I, as well as the non-switchable *Bpi*I-associated methylases M2.*Hpy*AII and M1.*Mbo*II, fully methylated all the sites of the associated endonuclease hindering its cutting activity at these sites. The switch methylases M.*Osp*807II (*Bsa*I-associated) and M2.*Nme*MC58II (*Bpi*I-associated) fully methylated specific sites of the endonuclease thus blocking digestion at those sites. Although the rest of our methylases did not exhibit as high activity as those mentioned above and generated partially methylated DNA, they could still be valuable. The use of non-switchable and switch methylases along with type IIS restriction enzyme partners could be used to develop DNA assembly techniques.

This work demonstrates the production of recombinant methylases with high *in vitro* activity for blocking the action of the type IIS restriction enzymes *Bsa*I, *Bpi*I, and *Lgu*I. Recombinant technology improves the protein production yield, supporting the feasibility of using these enzymes for industrial activity. Using these enzymes *in vitro* has several advantages over *in vivo* use as the enzymatic reactions and their conditions are independent of those necessitated by the producing host cells. The non-switchable methylases were able to block the endonuclease activity in all their sites, while the switch methylases allowed the possibility of creating specific endonuclease sites where digestion was blocked and other sites where it was not. Finally, this work also provides further insights into the feasibility of applying DNA methylases and type IIS restriction enzymes to expand the range of existing molecular tools, including those for DNA assembly.

##  Supplementary information

ESM 1 (PDF 2509 kb)
