# Supplementary material for: DNA methylases for site-selective inhibition of type IIS restriction enzyme activity
Source: Appl Microbiol Biotechnol. 2024 Jan 25;108(1):174. doi: 10.1007/s00253-024-13015-7 (PMC10810934; doi:10.1007/s00253-024-13015-7)
Supplement: Supplementary file 1 — (PDF 2509 kb) [file 253_2024_13015_MOESM1_ESM.pdf]

Supplementary Information

DNA methylases for site-selective inhibition of type IIS restriction enzyme activity

Applied Microbiology and Biotechnology

Carol N. Flores-Fernández[1], Da Lin[1†], Katherine Robins[1‡], Chris A. O'Callaghan[1*]

[1]Wellcome Trust Centre for Human Genetics, Nuffield Department of Medicine, University of Oxford, Roosevelt Drive, Oxford OX3 7BN, UK

[†]Current address: Triple Helix Biotechnology Ltd, Moneta Building (B280), Babraham Research Campus, Babraham, CB22 3AT, UK

[‡]Current address: Complete Regulatory, 19-20 King Edward Street, Macclesfield, SK10 1AQ, UK

*Corresponding author

E-mail: chris.ocallaghan@ndm.ox.ac.uk

Table S1 Methylases for cloning and expression

Methylase	Original source	Type	Protein Id	UniProtKB	EC number
M2.*Eco*31I	*E. coli* RFL31	Type IIS DNA 5-cytosine methylase	AAM09639.1	Q8RNY6	2.1.1.37
M2.*Bsa*I	*B. stearothermophilus* 6-55	Type IIS DNA 5-cytosine methylase	AAR96017.1	Q6SPF5	2.1.1.37
M.*Osp*807II	*Olsenella* sp. oral taxon 807 F0089	Type II N-6 adenine-specific DNA methylase	WP_083443746.1	Not found	2.1.1.72
M2.*Nme*MC58II	*N. meningitidis* B MC58 kan	Type IIS N-6 adenine-specific DNA methylase	AAF41140.1	Q9K090	2.1.1.72
M.*Sen*0738I[a]	*S. enterica* subsp. *enterica* serovar Thompson CFSAN000738	Type I R-M system N-6 adenine-specific DNA methylase (M) subunit	APW12375.1	A0A3Y8CIH9	2.1.1.72
S.*Sen*0738I[a]		Type I R-M system specificity (S) subunit	APW12376.1	A0A3Y6H358	-
M1.*Eco*31I	*E. coli* RFL31	Type IIS N-6 adenine-specific DNA methylase	AAM09640.1	Q8RNY5	2.1.1.72
M.*Xmn*I	*X. manihotis* 7AS1	Type II N-6 adenine-specific DNA methylase	AAC44403.1	Q56788	2.1.1.72
M1.*Hpy*AII	*H. pylori* 26695	Type IIS N-6 adenine-specific DNA methylase	AAD08411.1	O25920	2.1.1.72
M2.*Hpy*AII	*H. pylori* 26695	Type IIS N-4 cytosine-specific DNA methylase	AAD08412.1	O25921	2.1.1.113
M1.*Mbo*II	*M. bovis* ATCC 10900	Type IIS N-6 adenine-specific DNA methylase	CAA40297.1	P23192	2.1.1.72

[a]M.*Sen*0738I M subunit forms a complex with S.*Sen*0738I S subunit (M_2S) for methylation.

Table S2 Primers used in the PCR amplification of the methylases

**Primer name**	**Sequence**[a]	**Tm (°C)**
CO9301	5'-CTAGC**CATATG**ATTCCAAACCACAAGG-3'	59
CO9302	5'-CTAGC**CATATG**ACCAAGTCTGAAACATTC-3'	59
CO9303	5'-CTAG**AAGCTT**AAGCGACGGCTTAATGTTGC-3'	62
CO9304	5'-CTAG**GGATCC**TTAAAGCGACGGCTTAATG-3'	62
CO9305	5'-CTAGC**CATATG**ATTCCCAACCACGTATC-3'	60
CO9306	5'-CTAGC**CATATG**CTTATCATTAAACACGTGCC-3'	61
CO9307	5'-CTAG**AAGCTT**AATGATAGCTAAGGAGGACAGC-3'	61
CO9308	5'-CTAG**GGATCC**TTAAATGATAGCTAAGGAGGACAG-3'	61
CO9309	5'-CTAGC**CATATG**GCAAAGAAGGACACAAAC-3'	61
CO9310	5'-CTAG**CTCGAG**TTCCCCTTCTTTAAACTCGTAAAC-3'	63
CO9311	5'-CTAG**GGATCC**TTATTCCCCTTCTTTAAACTCG-3'	61
CO9312	5'-CTAGC**CATATG**ATCACAATCTCAAACGAAG-3'	59
CO9313	5'-CTAG**AAGCTT**TTCGATGATACAGTAGCCAG-3'	60
CO9314	5'-CTAG**GGATCC**TTATTCGATGATACAGTAGCCAG-3'	62
CO9315	5'-CTAGC**CATATG**TCAATCTCATCTGCTATTAAAAGC-3'	60
CO9316	5'-CTAG**CTCGAG**GTTCGCCTCTTTACCACTC-3'	64
CO9317	5'-CTAG**CTCGAG**TTAGTTCGCCTCTTTACCACTC-3'	64
CO9318	5'-CTAGC**CATATG**GCGGTGGAAAAACTTATTGTAG-3'	62
CO9319	5'-CTAG**CTCGAG**GTTAATTGCGGCATCAGTCAGG-3'	66
CO9320	5'-CTAG**GGATCC**TTAGTTAATTGCGGCATCAGTCAG-3'	64
CO9323	5'-CTAGC**GCTAGC**ATGGAGGAGATCTTCTATATGAAAC-3'	63
CO9324	5'-CTAG**GGATCC**TTAATCGTAGTGCTGGAAGATG-3'	62
CO9325	5'-CTAG**CCATGG**AGGAGATCTTCTATATGAAAC-3'	59
CO9326	5'-CTAG**AAGCTT**ATCGTAGTGCTGGAAGATG-3'	60
CO9331	5'-CTAGC**GCTAGC**ATGCGTGATCTTGCTAGTACTTATCG-3'	66
CO9332	5'-CTAG**AAGCTT**TTACCCGGCGCGCAAACC-3'	67
CO9333	5'-CTAG**CCATGG**GTGATCTTGCTAGTACTTATCGTCGTG-3'	66
CO9334	5'-CTAG**AAGCTT**CCCGGCGCGCAAACCATC-3'	68
CO9339	5'-CTAGC**CATATG**AACATTAATAAGGTGTTCTACC-3'	58
CO9341	5'-CTAG**GCGGCCGC**TTCAAAGTCAAATAAATTGCCC-3'	67
CO9342	5'-CTAGC**CATATG**GTAGAGAACATGTTAGAG-3'	57
CO9344	5'-CTAG**AAGCTT**GTTAATCTCCAACTGGTTC-3'	59
CO9452	5'-CCTAG**CATATG**AACATTAATAAGGTGTTCTACCAT-3'	59
CO9453	5'-CTAG**GCGGCCGC**TTATTCAAAGTCAAATAAATTGCCC-3'	67
CO9454	5'-CCTAG**CATATG**GTAGAGAACATGTTAGAGATC-3'	59
CO9455	5'-CTAG**GCGGCCGC**TTAGTTAATCTCCAACTGGTTCAGC-3'	69
CO9384	5'-CTAGC**CATATG**ATTCTGAATAAGATCTACATTGAG-3'	58
CO9385	5'-CTAG**GGATCC**TTATTCACACTCGTTATAGCG-3'	61
CO9386	5'-CTAG**GCGGCCGC**TTCACACTCGTTATAGCG-3'	68

[a]Nucleotides in **bold** and underlined correspond to the restriction site of the enzyme.

Gene sequences of methylases

M2.*Eco*31I (POC1463-KPL01)

ATGACCAAGTCTGAAACATTCATGATTCCAAACCACAAGGCCGCCAAATTAAGTGAGCT
GGATATGATGATCGTTAACTCTGTCCCGCCTGGGGAAACTGGAAGAATATTCCCTTGG
ATGTACCATCGAAACGTATCGAACAGATTCGTGACAGCTATGCTCAAGGAAAAGGGTCG
CGCAGCACATACTACGGGCGTTTATTGCCCGATATGCCAGCTTATACGATCAATACTTA
TTTCAATCGTCCTGGCAACGGATGCCACATTCACTACGAGCAAGATCGCGTACTTTCAC
AACGCGAAGCTGCACGTCTGCAGTCGTTCCCTGATGACTTTATCTTTTTTGGAGGTCAA
ACGGCGATTAATACGCAAATCGGTAATGCCGTGCCTCCCTTTCTTGCGTTTCTTATTGC
AAAAGAAATTGAAAAAGCGATCGGTAATACCGGCTACTACATTGACTTATTCAGTGGTG
CAGGCGGATTGGGGTTGGGCTTTAAGTGGGCCGGGTGGACTCCATTGTTAGCTAATGAC
ATTGAGGAAAAGTACTTACAGACATACTCGAACAACGTACACAAAGAAGTTTTGTGCGG
AAGCATTTCGGACAACGAAACTTTTTCTAAGATCGCAGACAAGATTTCTGGCTTTAAGA
AATTATATTTTGATAAACAGCTGTGGATTCTGGGCGGGCCTCCGTGCCAGGGATTTAGC
ACGGCTGGCAACGCGCGTACAATGGACGACCCACGCAACAGTCTGTTTATGCACTACAA
GTCGCTGCTTAACGAGATTAAGCCGAATGGATTCATTTTCGAGAACGTCGCCGGCCTGT
TGAACATGGAAAAAGGAAAGGTCTTTGAACGTGTTAAGGAGGAATTCTCGTCCACAATG
AAAACCATGAATGGTTGGATTTTAAATTCGGAACATTACGCAATTCCACAACGCCGTAA
GCGTGTAATTCTTGTGGGCAGCAATGATCCGCTGTTCTCGATCGAACCACCTCAGAAGC
TGACGGAAGATAAAGAGTCTTGGGTGTCAGTAAAAGATGCGTTATCTGACCTTCCCCCA
TTACAACACGGCGAGGATGGATCTGGTAAATACTATATCCACCACCCGGAAAATGATTA
CCAGTTGTTTATGCGTGGAAACATTACACCCTCAGAGTATTATGAACGCAACATTAAGC
CGTCGCTTTAA

M2.*Eco*31I (POC1464-KPL02)

ATGACCAAGTCTGAAACATTCATGATTCCAAACCACAAGGCCGCCAAATTAAGTGAGCT
GGATATGATGATCGTTAACTCTGTCCCGCCTGGGGAAACTGGAAGAATATTCCCTTGG
ATGTACCATCGAAACGTATCGAACAGATTCGTGACAGCTATGCTCAAGGAAAAGGGTCG
CGCAGCACATACTACGGGCGTTTATTGCCCGATATGCCAGCTTATACGATCAATACTTA
TTTCAATCGTCCTGGCAACGGATGCCACATTCACTACGAGCAAGATCGCGTACTTTCAC
AACGCGAAGCTGCACGTCTGCAGTCGTTCCCTGATGACTTTATCTTTTTTGGAGGTCAA
ACGGCGATTAATACGCAAATCGGTAATGCCGTGCCTCCCTTTCTTGCGTTTCTTATTGC
AAAAGAAATTGAAAAAGCGATCGGTAATACCGGCTACTACATTGACTTATTCAGTGGTG
CAGGCGGATTGGGGTTGGGCTTTAAGTGGGCCGGGTGGACTCCATTGTTAGCTAATGAC
ATTGAGGAAAAGTACTTACAGACATACTCGAACAACGTACACAAAGAAGTTTTGTGCGG
AAGCATTTCGGACAACGAAACTTTTTCTAAGATCGCAGACAAGATTTCTGGCTTTAAGA
AATTATATTTTGATAAACAGCTGTGGATTCTGGGCGGGCCTCCGTGCCAGGGATTTAGC
ACGGCTGGCAACGCGCGTACAATGGACGACCCACGCAACAGTCTGTTTATGCACTACAA
GTCGCTGCTTAACGAGATTAAGCCGAATGGATTCATTTTCGAGAACGTCGCCGGCCTGT
TGAACATGGAAAAAGGAAAGGTCTTTGAACGTGTTAAGGAGGAATTCTCGTCCACAATG
AAAACCATGAATGGTTGGATTTTAAATTCGGAACATTACGCAATTCCACAACGCCGTAA
GCGTGTAATTCTTGTGGGCAGCAATGATCCGCTGTTCTCGATCGAACCACCTCAGAAGC
TGACGGAAGATAAAGAGTCTTGGGTGTCAGTAAAAGATGCGTTATCTGACCTTCCCCCA
TTACAACACGGCGAGGATGGATCTGGTAAATACTATATCCACCACCCGGAAAATGATTA

CCAGTTGTTTATGCGTGGAAACATTACACCCTCAGAGTATTATGAACGCAACATTAAGC
CGTCGCTTTAA

M2.*Eco*31I_2 (POC1465-KPL03), truncated M2.*Eco*31I

ATGATTCCAAACCACAAGGCCGCCAAATTAAGTGAGCTGGATATGATGATCGTTAACTC
TGTCCCGCCTGGGGGAAACTGGAAGAATATTCCCTTGGATGTACCATCGAAACGTATCG
AACAGATTCGTGACAGCTATGCTCAAGGAAAAGGGTCGCGCAGCACATACTACGGGCGT
TTATTGCCCGATATGCCAGCTTATACGATCAATACTTATTTCAATCGTCCTGGCAACGG
ATGCCACATTCACTACGAGCAAGATCGCGTACTTTCACAACGCGAAGCTGCACGTCTGC
AGTCGTTCCCTGATGACTTTATCTTTTTTGGAGGTCAAACGGCGATTAATACGCAAATC
GGTAATGCCGTGCCTCCCTTTCTTGCGTTTCTTATTGCAAAAGAAATTGAAAAAGCGAT
CGGTAATACCGGCTACTACATTGACTTATTCAGTGGTGCAGGCGGATTGGGGTTGGGCT
TTAAGTGGGCCGGGTGGACTCCATTGTTAGCTAATGACATTGAGGAAAAGTACTTACAG
ACATACTCGAACAACGTACACAAAGAAGTTTTGTGCGGAAGCATTTCGGACAACGAAAC
TTTTTCTAAGATCGCAGACAAGATTTCTGGCTTTAAGAAATTATATTTTGATAAACAGC
TGTGGATTCTGGGCGGGCCTCCGTGCCAGGGATTTAGCACGGCTGGCAACGCGCGTACA
ATGGACGACCCACGCAACAGTCTGTTTATGCACTACAAGTCGCTGCTTAACGAGATTAA
GCCGAATGGATTCATTTTCGAGAACGTCGCCGGCCTGTTGAACATGGAAAAAGGAAAGG
TCTTTGAACGTGTTAAGGAGGAATTCTCGTCCACAATGAAAACCATGAATGGTTGGATT
TTAAATTCGGAACATTACGCAATTCCACAACGCCGTAAGCGTGTAATTCTTGTGGGCAG
CAATGATCCGCTGTTCTCGATCGAACCACCTCAGAAGCTGACGGAAGATAAAGAGTCTT
GGGTGTCAGTAAAAGATGCGTTATCTGACCTTCCCCCATTACAACACGGCGAGGATGGA
TCTGGTAAATACTATATCCACCACCCGGAAAATGATTACCAGTTGTTTATGCGTGGAAA
CATTACACCCTCAGAGTATTATGAACGCAACATTAAGCCGTCGCTTTAA

M2.*Eco*31I_2 (POC1466-KPL04), truncated M2.*Eco*31I

ATGATTCCAAACCACAAGGCCGCCAAATTAAGTGAGCTGGATATGATGATCGTTAACTC
TGTCCCGCCTGGGGGAAACTGGAAGAATATTCCCTTGGATGTACCATCGAAACGTATCG
AACAGATTCGTGACAGCTATGCTCAAGGAAAAGGGTCGCGCAGCACATACTACGGGCGT
TTATTGCCCGATATGCCAGCTTATACGATCAATACTTATTTCAATCGTCCTGGCAACGG
ATGCCACATTCACTACGAGCAAGATCGCGTACTTTCACAACGCGAAGCTGCACGTCTGC
AGTCGTTCCCTGATGACTTTATCTTTTTTGGAGGTCAAACGGCGATTAATACGCAAATC
GGTAATGCCGTGCCTCCCTTTCTTGCGTTTCTTATTGCAAAAGAAATTGAAAAAGCGAT
CGGTAATACCGGCTACTACATTGACTTATTCAGTGGTGCAGGCGGATTGGGGTTGGGCT
TTAAGTGGGCCGGGTGGACTCCATTGTTAGCTAATGACATTGAGGAAAAGTACTTACAG
ACATACTCGAACAACGTACACAAAGAAGTTTTGTGCGGAAGCATTTCGGACAACGAAAC
TTTTTCTAAGATCGCAGACAAGATTTCTGGCTTTAAGAAATTATATTTTGATAAACAGC
TGTGGATTCTGGGCGGGCCTCCGTGCCAGGGATTTAGCACGGCTGGCAACGCGCGTACA
ATGGACGACCCACGCAACAGTCTGTTTATGCACTACAAGTCGCTGCTTAACGAGATTAA
GCCGAATGGATTCATTTTCGAGAACGTCGCCGGCCTGTTGAACATGGAAAAAGGAAAGG
TCTTTGAACGTGTTAAGGAGGAATTCTCGTCCACAATGAAAACCATGAATGGTTGGATT
TTAAATTCGGAACATTACGCAATTCCACAACGCCGTAAGCGTGTAATTCTTGTGGGCAG
CAATGATCCGCTGTTCTCGATCGAACCACCTCAGAAGCTGACGGAAGATAAAGAGTCTT
GGGTGTCAGTAAAAGATGCGTTATCTGACCTTCCCCCATTACAACACGGCGAGGATGGA
TCTGGTAAATACTATATCCACCACCCGGAAAATGATTACCAGTTGTTTATGCGTGGAAA
CATTACACCCTCAGAGTATTATGAACGCAACATTAAGCCGTCGCTTTAA

M2.*Bsa*I (POC1467-KPL05)

ATGATTCCCAACCACGTATCTTCCAAGTTAAGTGAACTTGACATGCTTATCATTAAACA
CGTGCCGCCCGGAGGAAATTGGAAAGACATCCCCGAATGGGTGCCGTCTAAGCGTTTGG
AGCAAATTCGTAAGTCTTATGCTGAGGGAAAAGGATCACGCTCAACTTACTATGGCCGC
CTGTTACCAGATATGCCAAGCTACACTATCAACACGTACTTCAACCGTCCAGGTAATGG
GTGCCACATCCATTATGAACAGGATCGCACTCTGTCACAGCGTGAGGCCGCGCGCTTGC
AGTCTTTCCCTGACGATTTCATCTTCTACGGTAGCAAGACGGCCATCAACAACCAGATC
GGCAACGCTGTTCCGCCCCTTCTTGCTTATCAGATCGCCAAGGCATTTCCCTTCAAAGG
CCAATTTGTCGATCTTTTTAGTGGGGCGGGGGGCCTTTCCTTAGGTTTCCTTTGGGCCG
GGTGGAAACCGATCATCGCAAATGACATCGATAAATGGGCGCTGACCACCTACATGAAT
AATATTCACAATGAAGTAGTGCTTGGGGATATCCGCGACGAAAAGGTCAGTGAGACTAT
CATTCAAAAGTGCCTGATCGCAAAGAAGTCGAACCCGGACCGTCCATTGTTTGTTCTTG
GGGGACCACCCTGCCAAGGGTTTTCGACTGCAGGAAAAAAACGTTCAATCGTGGACGAG
CGCAACTGGCTGTTCGAAAGTTACGTGAGTATCCTGAAAGAAGTCAAGCCAGACGGTTT
TATTTTTGAAAACGTGACTGGCCTGTTGTCGATGGAGAAGGGAGCATTCTTTGAGATGG
TTAAATCGGAATTGTCTAAGACGGTGTCGAATCTTTTCGTATATAAACTTAATTCGGTG
GATTACGGTGTGCCCCAACGCCGCAACCGCGTAGTCATCATCGGGGACTCCACCGGAAC
GAAGAACAGCGAGCCCCCTATCCCAATTACGTCTCTTAAAGGAGAAAAGACCCTGTTTG
ACGCCTTAAGCAGTGCCATTTCCGTAAAGGAGGCCCTTTCAGACTTACCCCTTTTGTCG
CCTAATGAGGACGGCTCTTGGAAGAACTACGTTTGTGAGCCACAAAATATTTATCAGTC
ATTCATGCGCAAGAAGATCACAGCCCAGCAGTATATCGAGATGCTGTCCTCCTTAGCTA
TCATTTAA

M2.*Bsa*I (POC1468-KPL06)

ATGATTCCCAACCACGTATCTTCCAAGTTAAGTGAACTTGACATGCTTATCATTAAACA
CGTGCCGCCCGGAGGAAATTGGAAAGACATCCCCGAATGGGTGCCGTCTAAGCGTTTGG
AGCAAATTCGTAAGTCTTATGCTGAGGGAAAAGGATCACGCTCAACTTACTATGGCCGC
CTGTTACCAGATATGCCAAGCTACACTATCAACACGTACTTCAACCGTCCAGGTAATGG
GTGCCACATCCATTATGAACAGGATCGCACTCTGTCACAGCGTGAGGCCGCGCGCTTGC
AGTCTTTCCCTGACGATTTCATCTTCTACGGTAGCAAGACGGCCATCAACAACCAGATC
GGCAACGCTGTTCCGCCCCTTCTTGCTTATCAGATCGCCAAGGCATTTCCCTTCAAAGG
CCAATTTGTCGATCTTTTTAGTGGGGCGGGGGGCCTTTCCTTAGGTTTCCTTTGGGCCG
GGTGGAAACCGATCATCGCAAATGACATCGATAAATGGGCGCTGACCACCTACATGAAT
AATATTCACAATGAAGTAGTGCTTGGGGATATCCGCGACGAAAAGGTCAGTGAGACTAT
CATTCAAAAGTGCCTGATCGCAAAGAAGTCGAACCCGGACCGTCCATTGTTTGTTCTTG
GGGGACCACCCTGCCAAGGGTTTTCGACTGCAGGAAAAAAACGTTCAATCGTGGACGAG
CGCAACTGGCTGTTCGAAAGTTACGTGAGTATCCTGAAAGAAGTCAAGCCAGACGGTTT
TATTTTTGAAAACGTGACTGGCCTGTTGTCGATGGAGAAGGGAGCATTCTTTGAGATGG
TTAAATCGGAATTGTCTAAGACGGTGTCGAATCTTTTCGTATATAAACTTAATTCGGTG
GATTACGGTGTGCCCCAACGCCGCAACCGCGTAGTCATCATCGGGGACTCCACCGGAAC
GAAGAACAGCGAGCCCCCTATCCCAATTACGTCTCTTAAAGGAGAAAAGACCCTGTTTG
ACGCCTTAAGCAGTGCCATTTCCGTAAAGGAGGCCCTTTCAGACTTACCCCTTTTGTCG
CCTAATGAGGACGGCTCTTGGAAGAACTACGTTTGTGAGCCACAAAATATTTATCAGTC
ATTCATGCGCAAGAAGATCACAGCCCAGCAGTATATCGAGATGCTGTCCTCCTTAGCTA
TCATTTAA

M2.*Bsa*I_2 (POC1469-KPL07), truncated M2.*Bsa*I

ATGCTTATCATTAAACACGTGCCGCCCGGAGGAAATTGGAAAGACATCCCCGAATGGGT
GCCGTCTAAGCGTTTGGAGCAAATTCGTAAGTCTTATGCTGAGGGAAAAGGATCACGCT
CAACTTACTATGGCCGCCTGTTACCAGATATGCCAAGCTACACTATCAACACGTACTTC
AACCGTCCAGGTAATGGGTGCCACATCCATTATGAACAGGATCGCACTCTGTCACAGCG
TGAGGCCGCGCGCTTGCAGTCTTTCCCTGACGATTTCATCTTCTACGGTAGCAAGACGG
CCATCAACAACCAGATCGGCAACGCTGTTCCGCCCCTTCTTGCTTATCAGATCGCCAAG
GCATTTCCCTTCAAAGGCCAATTTGTCGATCTTTTTAGTGGGGCGGGGGGCCTTTCCTT
AGGTTTCCTTTGGGCCGGGTGGAAACCGATCATCGCAAATGACATCGATAAATGGGCGC
TGACCACCTACATGAATAATATTCACAATGAAGTAGTGCTTGGGGATATCCGCGACGAA
AAGGTCAGTGAGACTATCATTCAAAAGTGCCTGATCGCAAAGAAGTCGAACCCGGACCG
TCCATTGTTTGTTCTTGGGGGACCACCCTGCCAAGGGTTTTCGACTGCAGGAAAAAAAC
GTTCAATCGTGGACGAGCGCAACTGGCTGTTCGAAAGTTACGTGAGTATCCTGAAAGAA
GTCAAGCCAGACGGTTTTATTTTTGAAAACGTGACTGGCCTGTTGTCGATGGAGAAGGG
AGCATTCTTTGAGATGGTTAAATCGGAATTGTCTAAGACGGTGTCGAATCTTTTCGTAT
ATAAACTTAATTCGGTGGATTACGGTGTGCCCCAACGCCGCAACCGCGTAGTCATCATC
GGGGACTCCACCGGAACGAAGAACAGCGAGCCCCCTATCCCAATTACGTCTCTTAAAGG
AGAAAAGACCCTGTTTGACGCCTTAAGCAGTGCCATTTCCGTAAAGGAGGCCCTTTCAG
ACTTACCCCTTTTGTCGCCTAATGAGGACGGCTCTTGGAAGAACTACGTTTGTGAGCCA
CAAAATATTTATCAGTCATTCATGCGCAAGAAGATCACAGCCCAGCAGTATATCGAGAT
GCTGTCCTCCTTAGCTATCATTTAA

M2.*Bsa*I_2 (POC1470-KPL08), truncated M2.*Bsa*I

ATGCTTATCATTAAACACGTGCCGCCCGGAGGAAATTGGAAAGACATCCCCGAATGGGT
GCCGTCTAAGCGTTTGGAGCAAATTCGTAAGTCTTATGCTGAGGGAAAAGGATCACGCT
CAACTTACTATGGCCGCCTGTTACCAGATATGCCAAGCTACACTATCAACACGTACTTC
AACCGTCCAGGTAATGGGTGCCACATCCATTATGAACAGGATCGCACTCTGTCACAGCG
TGAGGCCGCGCGCTTGCAGTCTTTCCCTGACGATTTCATCTTCTACGGTAGCAAGACGG
CCATCAACAACCAGATCGGCAACGCTGTTCCGCCCCTTCTTGCTTATCAGATCGCCAAG
GCATTTCCCTTCAAAGGCCAATTTGTCGATCTTTTTAGTGGGGCGGGGGGCCTTTCCTT
AGGTTTCCTTTGGGCCGGGTGGAAACCGATCATCGCAAATGACATCGATAAATGGGCGC
TGACCACCTACATGAATAATATTCACAATGAAGTAGTGCTTGGGGATATCCGCGACGAA
AAGGTCAGTGAGACTATCATTCAAAAGTGCCTGATCGCAAAGAAGTCGAACCCGGACCG
TCCATTGTTTGTTCTTGGGGGACCACCCTGCCAAGGGTTTTCGACTGCAGGAAAAAAAC
GTTCAATCGTGGACGAGCGCAACTGGCTGTTCGAAAGTTACGTGAGTATCCTGAAAGAA
GTCAAGCCAGACGGTTTTATTTTTGAAAACGTGACTGGCCTGTTGTCGATGGAGAAGGG
AGCATTCTTTGAGATGGTTAAATCGGAATTGTCTAAGACGGTGTCGAATCTTTTCGTAT
ATAAACTTAATTCGGTGGATTACGGTGTGCCCCAACGCCGCAACCGCGTAGTCATCATC
GGGGACTCCACCGGAACGAAGAACAGCGAGCCCCCTATCCCAATTACGTCTCTTAAAGG
AGAAAAGACCCTGTTTGACGCCTTAAGCAGTGCCATTTCCGTAAAGGAGGCCCTTTCAG
ACTTACCCCTTTTGTCGCCTAATGAGGACGGCTCTTGGAAGAACTACGTTTGTGAGCCA
CAAAATATTTATCAGTCATTCATGCGCAAGAAGATCACAGCCCAGCAGTATATCGAGAT
GCTGTCCTCCTTAGCTATCATTTAA

M.*Osp*807II (POC1471-KPL09)

ATGGCAAAGAAGGACACAAACCTTAAGCTTTCCCACCTTTATAAGGGTAACGTATCGGA
AGTCTACGGGCGCTGGCCTAGTCCGGACTTAATTGTATCGGATGGGGCATACGGTGTTC
GTGGATTTCGCGGGGACACCGTTGACGCTGCCGGGTTGACGGACTGGTATAAGCCACAC
GTGCTGGCGTGGGCCAAAGCGGCCAAGCCTTCCACTTCTTTGTGGTTTTGGAACACAGA
AGTAGGCTGGGCCACGGTCCACCCTTTATTATTGTCCACCGGATGGGAATACGTGCAGT
TAGCTGTCTGGGATAAAGGACTGGCGCACATTGCCGGCAATGTTAACGGGAAAACAATC
CGTCAATTGCCCGTGGTAACAGAGGTGGCGGCCCTTTATCGCCGTACGGTGTACCTGGA
AACTGGAGACGGACTTACATTAAACGCAAAGTCATGGTTGCGCGCAGAGTGGCGTCGCT
CGGGCCTGTCCCTTTCAAAGTCGAATGAAGCGTGTGGTGTGAAGAACGCTGCGACCCGT
AAATATTTGACAGCAGACTGGCTGTGGTACTGGCCGCCTGGTGACGCGGTCCAAAAGAT
GGCGGAGTACTGCATGCAATATGGTAAAAAAACTTCTTGGCCCTATTTCAGTCTGGATG
GCAAAACTATGATTTCAGCTCATGATTGGGATAGTTTACGCACTACATGGAATCACCGC
AATGGAGTCACAAACGTGTGGAGCCGTCCGCCGTTGGCCGATAGTGAGCGTTTGAAAGG
GACAATGGAACGCTCCGCACCTCGCACGTATAAACCCACCAAACAGTCAGCAGCACATC
TGAACCAAAAGCCCCTGGACCTGATGCTTACTCAGGTAGCAGCAGCCAGTAATGTCGGC
GATACAGTCTGGGAACCATTTGGAGGTCTGTGCTCGGCTAGTGTGGCATCGTCATTACT
TGGTCGCCGCAGTTACGCTGCAGAAATTGACGACACATTTTACAAGTTAGCAGCGGCCC
GCCTGAATGAGGCAAATGCCTATTTTGAATCGAACGGTGTTTACGAGTTTAAAGAAGGG
GAATAA

M.*Osp*807II (POC1472-KPL10)

ATGGCAAAGAAGGACACAAACCTTAAGCTTTCCCACCTTTATAAGGGTAACGTATCGGA
AGTCTACGGGCGCTGGCCTAGTCCGGACTTAATTGTATCGGATGGGGCATACGGTGTTC
GTGGATTTCGCGGGGACACCGTTGACGCTGCCGGGTTGACGGACTGGTATAAGCCACAC
GTGCTGGCGTGGGCCAAAGCGGCCAAGCCTTCCACTTCTTTGTGGTTTTGGAACACAGA
AGTAGGCTGGGCCACGGTCCACCCTTTATTATTGTCCACCGGATGGGAATACGTGCAGT
TAGCTGTCTGGGATAAAGGACTGGCGCACATTGCCGGCAATGTTAACGGGAAAACAATC
CGTCAATTGCCCGTGGTAACAGAGGTGGCGGCCCTTTATCGCCGTACGGTGTACCTGGA
AACTGGAGACGGACTTACATTAAACGCAAAGTCATGGTTGCGCGCAGAGTGGCGTCGCT
CGGGCCTGTCCCTTTCAAAGTCGAATGAAGCGTGTGGTGTGAAGAACGCTGCGACCCGT
AAATATTTGACAGCAGACTGGCTGTGGTACTGGCCGCCTGGTGACGCGGTCCAAAAGAT
GGCGGAGTACTGCATGCAATATGGTAAAAAAACTTCTTGGCCCTATTTCAGTCTGGATG
GCAAAACTATGATTTCAGCTCATGATTGGGATAGTTTACGCACTACATGGAATCACCGC
AATGGAGTCACAAACGTGTGGAGCCGTCCGCCGTTGGCCGATAGTGAGCGTTTGAAAGG
GACAATGGAACGCTCCGCACCTCGCACGTATAAACCCACCAAACAGTCAGCAGCACATC
TGAACCAAAAGCCCCTGGACCTGATGCTTACTCAGGTAGCAGCAGCCAGTAATGTCGGC
GATACAGTCTGGGAACCATTTGGAGGTCTGTGCTCGGCTAGTGTGGCATCGTCATTACT
TGGTCGCCGCAGTTACGCTGCAGAAATTGACGACACATTTTACAAGTTAGCAGCGGCCC
GCCTGAATGAGGCAAATGCCTATTTTGAATCGAACGGTGTTTACGAGTTTAAAGAAGGG
GAATAA

M2.*Nme*MC58II (POC1473-KPL11)

ATGATCACAATCTCAAACGAAGATAATATGATCCTGATGAGTCGCTATCCAGACAAGTA
CTTTGACCTGGCCATTGTTGACCCCCCTTATGGAATCTTGAACAAAACTAAACGCGGTG
GAGATTATAAATTCAACATGAACGAATACAGCCAGTGGGATATTAAACCTGACCAGACC
TACTTCAACGAGTTATTTCGCGTTAGCAAGAATCAGATTATTTGGGGGGGCAACTATTT
TGGAGAATTGTGGTTACGTAGCGAATATAATAAGGGCTTCATTATCTGGGACAAGAACC
AACCGGAAACCTTAAACAATTTTTCGATGGCAGAAATGGCCTGGTCAAGTTTCGATCGC
CCATCGAAGATTTTTCGCTTCAGTGTTCGTAAAAACCGCAACAAGACACATCCGACGCA
GAAGCCAGTAGAGCTTTACCAATGGTTATTGAAGATGTACGCAAAACAGGGTGATAAAA
TTTTGGATACACACCTGGGATCTGGGACTTTAGCGATTGCATGTTGTATTGCCCAATTT
GATTTAACAGCGTGTGAGATTAACTCTGATTATTACCAGCAGTCCATCGAGAAGATTAA
GAACAACTTGCCCGAAGCACGCATTTCCTTTGGCCACCCTGGCTACTGTATCATCGAAT
AA

M2.*Nme*MC58II (POC1474-KPL12)

ATGATCACAATCTCAAACGAAGATAATATGATCCTGATGAGTCGCTATCCAGACAAGTA
CTTTGACCTGGCCATTGTTGACCCCCCTTATGGAATCTTGAACAAAACTAAACGCGGTG
GAGATTATAAATTCAACATGAACGAATACAGCCAGTGGGATATTAAACCTGACCAGACC
TACTTCAACGAGTTATTTCGCGTTAGCAAGAATCAGATTATTTGGGGGGGCAACTATTT
TGGAGAATTGTGGTTACGTAGCGAATATAATAAGGGCTTCATTATCTGGGACAAGAACC
AACCGGAAACCTTAAACAATTTTTCGATGGCAGAAATGGCCTGGTCAAGTTTCGATCGC
CCATCGAAGATTTTTCGCTTCAGTGTTCGTAAAAACCGCAACAAGACACATCCGACGCA
GAAGCCAGTAGAGCTTTACCAATGGTTATTGAAGATGTACGCAAAACAGGGTGATAAAA
TTTTGGATACACACCTGGGATCTGGGACTTTAGCGATTGCATGTTGTATTGCCCAATTT
GATTTAACAGCGTGTGAGATTAACTCTGATTATTACCAGCAGTCCATCGAGAAGATTAA
GAACAACTTGCCCGAAGCACGCATTTCCTTTGGCCACCCTGGCTACTGTATCATCGAAT
AA

M.*Sen*0738I (POC1475-KPL13)

ATGTCAATCTCATCTGCTATTAAAAGCTTGCAAGACATCATGCGCAAAGATGCCGGTGT
TGATGGTGATGCCCAGCGTTTGGGCCAGCTTTCTTGGTTACTGTTTTTAAAAATCTTTG
ATGCACAGGAACAGGCACTGGAGATTGAACAAGAAAAGTATCGTCTGCCTATGCCAGAA
CGCTATTTATGGCGCAATTGGGCCGCCGATAATGAAGGAATTACAGGGGATAAATTGTT
GGCGTTCGTCAACGATGATCTTTTTCCCACCTTGAAGGACTTGCCCGCCCAGATCGACA
TCAACCCCCGCGGGTATGTAGTAAAGCAGGCTTTCTCCGACGCTTATAACTACATGAAA
AACGGGACGTTGCTGCGCCAAGTTATCAACAAGTTAAATGAGATTGACTTTACACGTGC
TTCAGAGCGCCATCTTTTTGGAGATATCTACGAGCAAATTTTGCGTGACTTGCAGGCTG
CCGGTAATGCTGGTGAATTTTATACGCCTCGCGCTGTTACTCGCTTCATGGTTGAACGT
GTAGACCCTAAACTGGGTGAGTCGATCATGGATCCGGCTTGTGGTACGGGGGGATTTTT
GGCATGCGCCTTCGACCACGTTAAAAACCATTATGCCCATACTGTGACTGACCATCAAA
TCTTGCAGAAACAAATTCATGGGGTCGAAAAGAAGCAATTACCACACTTGTTGTGTACG
ACCAACATGCTGCTTCACGGCATCGAAGTCCCCGTTCAAATCCGCCACGACAATACTCT
TAATAAACCACTTTCATCGTGGGACGAGCAAATGGATGTTATCATCACTAACCCCCCAT
TCGGGGGTACTGAGGAAGATGGCATTGAAAAGAACTTCCCCTCTGACATGCAGACACGT
GAGACGGCGGACTTATTTCTTCAGCTTATTATTGAGGTCTTAGCTAAGAACGGTCGTGC
CGCCGTGGTGTTGCCCGATGGTACGCTTTTTGGTGAAGGTGTTAAAACTAAGATTAAAA
AGTTGCTGACCGAAGAGTGCAATTTGCACACAATTGTCCGTTTACCTAACGGCGTTTTT

AACCCCTATACGGGGATCAAAACTAACCTGTTATTTTTCACGAAAGGGCAGCCGACCAA
GGAGATCTGGTTTTATGAACACCCTTACCCCGCAGGTGTTAAAAACTACAGCAAGACGA
AACCAATGAAATTTGAGGAGTTTCAGGCAGAAATTGATTGGTGGGGTAACGAAGCGGAT
GGGTTTGCAAGTCGCGTCGAGAATGAGCAGGCCTGGAAAGTCTCTATTGATGAAGTTAT
CGCCCGTAACTTCAATCTTGATATTAAGAACCCCCACCAGGCAGAaACCGTATCCCATG
ACCCAGACGAGCTGTTAGCACAATACGCCAAACAGCAAGAGGCCATCCAAACTCTTCGC
CATCAGCTTCGTGACATCTTAGGAACTGCGTTGAGTGGTAAAGAGGCGAACTAA

M.*Sen*0738I (POC1476-KPL14)

ATGTCAATCTCATCTGCTATTAAAAGCTTGCAAGACATCATGCGCAAAGATGCCGGTGT
TGATGGTGATGCCCAGCGTTTGGGCCAGCTTTCTTGGTTACTGTTTTTAAAAATCTTTG
ATGCACAGGAACAGGCACTGGAGATTGAACAAGAAAAGTATCGTCTGCCTATGCCAGAA
CGCTATTTATGGCGCAATTGGGCCGCCGATAATGAAGGAATTACAGGGGATAAATTGTT
GGCGTTCGTCAACGATGATCTTTTTCCCACCTTGAAGGACTTGCCCGCCCAGATCGACA
TCAACCCCCGCGGGTATGTAGTAAAGCAGGCTTTCTCCGACGCTTATAACTACATGAAA
AACGGGACGTTGCTGCGCCAAGTTATCAACAAGTTAAATGAGATTGACTTTACACGTGC
TTCAGAGCGCCATCTTTTTGGAGATATCTACGAGCAAATTTTGCGTGACTTGCAGGCTG
CCGGTAATGCTGGTGAATTTTATACGCCTCGCGCTGTTACTCGCTTCATGGTTGAACGT
GTAGACCCTAAACTGGGTGAGTCGATCATGGATCCGGCTTGTGGTACGGGGGGATTTTT
GGCATGCGCCTTCGACCACGTTAAAAACCATTATGCCCATACTGTGACTGACCATCAAA
TCTTGCAGAAACAAATTCATGGGGTCGAAAAGAAGCAATTACCACACTTGTTGTGTACG
ACCAACATGCTGCTTCACGGCATCGAAGTCCCCGTTCAAATCCGCCACGACAATACTCT
TAATAAACCACTTTCATCGTGGGACGAGCAAATGGATGTTATCATCACTAACCCCCCAT
TCGGGGGTACTGAGGAAGATGGCATTGAAAAGAACTTCCCCTCTGACATGCAGACACGT
GAGACGGCGGACTTATTTCTTCAGCTTATTATTGAGGTCTTAGCTAAGAACGGTCGTGC
CGCCGTGGTGTTGCCCGATGGTACGCTTTTTGGTGAAGGTGTTAAAACTAAGATTAAAA
AGTTGCTGACCGAAGAGTGCAATTTGCACACAATTGTCCGTTTACCTAACGGCGTTTTT
AACCCCTATACGGGGATCAAAACTAACCTGTTATTTTTCACGAAAGGGCAGCCGACCAA
GGAGATCTGGTTTTATGAACACCCTTACCCCGCAGGTGTTAAAAACTACAGCAAGACGA
AACCAATGAAATTTGAGGAGTTTCAGGCAGAAATTGATTGGTGGGGTAACGAAGCGGAT
GGGTTTGCAAGTCGCGTCGAGAATGAGCAGGCCTGGAAAGTCTCTATTGATGAAGTTAT
CGCCCGTAACTTCAATCTTGATATTAAGAACCCCCACCAGGCAGAaACCGTATCCCATG
ACCCAGACGAGCTGTTAGCACAATACGCCAAACAGCAAGAGGCCATCCAAACTCTTCGC
CATCAGCTTCGTGACATCTTAGGAACTGCGTTGAGTGGTAAAGAGGCGAACTAA

S.*Sen*0738I (POC1477-KPL15)

ATGGCGGTGGAAAAACTTATTGTAGATCATATCGACACCTGGACTACCGCCCTTCAGAC
GCGCTCCACGGCCGGTCGCGGGAGCAGCGGCAAAATCGACTTGTATGGAATTAAGAAGC
TTCGCGAACTTATTTTAGAACTGGCCGTACGTGGCAAGCTGGTCCCGCAAGACCCAAAT
GACAAGCCCGCTTCGGTGCTTTTAGAACGCATCGCGACAGAAAAAGCGGAGCTGGTGAA
ACAAGGTAAGATTAAAAAGCAGAAGCCCTTGCCAGAAATCTCTGAGGAGGAAAAGCCAT
TTGAACTGCCAGCGGGCTGGGAATGGGCTCGTTTAAATGAATTAGCTCCTATGGGAATC
ATTGACGGGGATTGGATCGAGTCAAAAGACCAGGACCCGTCAGGCGCTTACCGTTTGAT
TCAGTTAGCTGATGTTGGCGTGGGAGATTTCAAGGACAAGTCCGATCGCTATATCAACA
CTTCGACGTTTCATCGTTTGAACTGCCATCAACTGATGGAGGGTGATATCCTTATCGCG
CGCTTACCGAACCCGATCGGACGCGCGTGCATCTTTCCTAAGCTTTCCCAAAGCGCTAT
CACTGTAGTAGATATTGCTACAATGCGCCCTTCAGGAAATTATAGTGCGGAATATATTA
TTTCCGCCATTAACAGTTTAACATTTCGTCAGCAAGTAGAATCGTTTGGAAAGGGCGCG

ACGCGCTTCCGCATCGCAACCGGGCACCTGAAaACATTGCTTTTGCCAATTCCACCCGT
CCAGGAACAATATAGTATCTTCAAAAAAATTAAGGAGCTGATGTCCCTTTGCGATCAGT
TAGAACAATATAGCCTGACTTCCCTTGATGCACATCAACAATTAGTGGAAACGTTGTTA
ACCACCTTGACGGACAGCCAGAATGCAGATGAGTTAGCTGAAAATTGGGCCCGTATCAG
CGAACACTTCGATACTCTTTTTACAACTGAAGTCAGTATCGACGCCCTGAAGCAGACGA
TCCTGCAACTGGCTGTCATGGGTAAGCTGGTGCCACAGGACCCAAACGATGAACCAGCA
TCTGAACTGCTGAAGCGTATTGCACAGGAAAAAGCACAGTTGGTAAAGGATGGAAAGAT
GAAGAAGCAAAAGCCATTACCGCCGATTAGTGATGAGGAAAAACCATTCGAGTTACCAT
CGGGTTGGGAATGGTGTTTATTTGAGGACGTCGTAGATATTCAATCGGGCATTACCAAG
GGCCGCAACTTAGCAAATCGCAAGCTTATTTCTATCCCGTATTTGCGCGTAGCCAACGT
GCAGCGCGGTTATTTGGACCTTTCAGAAGTTAAAGAAATCGACATTCCCGAAGAAGAGA
AGGATAAGTACCACGTGATCAAGGGCGACTTGTTAATCACGGAAGGCGGCGATTGGGAC
ACAGTAGGGCGTACTACTGTTTGGTGCCACGACTGGTATATCGCCAATCAAAACCACGT
GTTTAAGGGACGTATTATCGGGCAGGACATCGATCCCTATTGGCTTGAGACGTACATGA
ACTCTCCTTACGCCCGTGATTACTTCGCGAGTGCCTCTAAACAAACTACCAACCTGGCG
AGTATCAACAAGACGCAACTTCGTGGATGTCCAGTGGCTATTCCACCTAGTAGCGAAGC
AGAAAAAATCATGCTTAAATTAAACGATTTTAATGAACTGTGTGAAAAATTAAAGCTGC
AGATTCAGAGCGCTCAACAGACGCAGCTTCATCTTGCGGACGCCCTGACTGATGCCGCA
ATTAACTAA

S.*Sen*0738I (POC1478-KPL16)

ATGGCGGTGGAAAAACTTATTGTAGATCATATCGACACCTGGACTACCGCCCTTCAGAC
GCGCTCCACGGCCGGTCGCGGGAGCAGCGGCAAAATCGACTTGTATGGAATTAAGAAGC
TTCGCGAACTTATTTTAGAACTGGCCGTACGTGGCAAGCTGGTCCCGCAAGACCCAAAT
GACAAGCCCGCTTCGGTGCTTTTAGAACGCATCGCGACAGAAAAAGCGGAGCTGGTGAA
ACAAGGTAAGATTAAAAAGCAGAAGCCCTTGCCAGAAATCTCTGAGGAGGAAAAGCCAT
TTGAACTGCCAGCGGGCTGGGAATGGGCTCGTTTAAATGAATTAGCTCCTATGGGAATC
ATTGACGGGGATTGGATCGAGTCAAAAGACCAGGACCCGTCAGGCGCTTACCGTTTGAT
TCAGTTAGCTGATGTTGGCGTGGGAGATTTCAAGGACAAGTCCGATCGCTATATCAACA
CTTCGACGTTTCATCGTTTGAACTGCCATCAACTGATGGAGGGTGATATCCTTATCGCG
CGCTTACCGAACCCGATCGGACGCGCGTGCATCTTTCCTAAGCTTTCCCAAAGCGCTAT
CACTGTAGTAGATATTGCTACAATGCGCCCTTCAGGAAATTATAGTGCGGAATATATTA
TTTCCGCCATTAACAGTTTAACATTTCGTCAGCAAGTAGAATCGTTTGGAAAGGGCGCG
ACGCGCTTCCGCATCGCAACCGGGCACCTGAAaACATTGCTTTTGCCAATTCCACCCGT
CCAGGAACAATATAGTATCTTCAAAAAAATTAAGGAGCTGATGTCCCTTTGCGATCAGT
TAGAACAATATAGCCTGACTTCCCTTGATGCACATCAACAATTAGTGGAAACGTTGTTA
ACCACCTTGACGGACAGCCAGAATGCAGATGAGTTAGCTGAAAATTGGGCCCGTATCAG
CGAACACTTCGATACTCTTTTTACAACTGAAGTCAGTATCGACGCCCTGAAGCAGACGA
TCCTGCAACTGGCTGTCATGGGTAAGCTGGTGCCACAGGACCCAAACGATGAACCAGCA
TCTGAACTGCTGAAGCGTATTGCACAGGAAAAAGCACAGTTGGTAAAGGATGGAAAGAT
GAAGAAGCAAAAGCCATTACCGCCGATTAGTGATGAGGAAAAACCATTCGAGTTACCAT
CGGGTTGGGAATGGTGTTTATTTGAGGACGTCGTAGATATTCAATCGGGCATTACCAAG
GGCCGCAACTTAGCAAATCGCAAGCTTATTTCTATCCCGTATTTGCGCGTAGCCAACGT
GCAGCGCGGTTATTTGGACCTTTCAGAAGTTAAAGAAATCGACATTCCCGAAGAAGAGA
AGGATAAGTACCACGTGATCAAGGGCGACTTGTTAATCACGGAAGGCGGCGATTGGGAC
ACAGTAGGGCGTACTACTGTTTGGTGCCACGACTGGTATATCGCCAATCAAAACCACGT
GTTTAAGGGACGTATTATCGGGCAGGACATCGATCCCTATTGGCTTGAGACGTACATGA
ACTCTCCTTACGCCCGTGATTACTTCGCGAGTGCCTCTAAACAAACTACCAACCTGGCG
AGTATCAACAAGACGCAACTTCGTGGATGTCCAGTGGCTATTCCACCTAGTAGCGAAGC
AGAAAAAATCATGCTTAAATTAAACGATTTTAATGAACTGTGTGAAAAATTAAAGCTGC

AGATTCAGAGCGCTCAACAGACGCAGCTTCATCTTGCGGACGCCCTGACTGATGCCGCA
ATTAACTAA

M1.*Eco*31I (POC1479-KPL17)

ATGGAGGAGATCTTCTATATGAAACACATCCATTTAATCAACAGCCTGTCGCTGGACGA
AACTACTAAGTTCACGAAAAAAGCTACCGGTAAATACTACACCGACCCCAAAATCGCGC
TGTTAATGATCGAGAAGTTACTTCCGCTGATTAACTCCTGTGATAAAAAGAGCTATAAC
GTTGCTGATCCCTTTTCAGGTGACGGTCGTCTGATCACTCTTTTGATCAAGCAGTGGAT
GATTAACGGCTTCCCCGATGTCGAATGGAACGTCTACCTGTTTGACATTGAGAACACCG
GTCTGACTTACGCCAAAAACGCTCTGTCGGAATTGAAGTTAGCCGGCGCTAATATCAAT
ATTACAATTAAGAACTCGGATGTATTTTACGAATTTAAGAAGTATGTAGACTACTTCGA
CTGTGTGATCACAAATCCTCCCTGGGAGAACATTAAACCTGATTCTCGTGAGCTTGATT
TTTTCGAACCAAGCATGAAATCCATGTATATTGACAGCCTTCGTGAATTTGATGATTAC
CTTTCACGTGTGCTTCCCTATAGTCAGCCGAAGCGCAAGTTCGCTGGCTGGGGTACGAA
CTTAAGTCGTGTTGGTGCAGAGTTATCTCTTGAGATTTGCAACAAAAATGGGTTAGTAG
CGATCGTCATGCCCGCCAGCTTTTTTGCGGACGAGCAATCCTATATTTTACGTGAGAAG
TTTTTCAATTCCGGACGTATCGACTGTATTAACTATTATCCAGCCGAAGCAAAACTTTT
CGGTGGAGCAGATGTGAGCTCCTGCTCCTTCATTTTTAACAAGGGAGAATCCTTGAATG
ATAATATTCAATTAAGCGTCTACGATAAAAATTTGAACATCAAGTCATTGGGTTTTTTC
GATTTGTCAAGCATCGATTCCCAATATCTGTCGATCCCCGTGTCTCAAGGCGTGCATGC
GGTCCATCTTTTGCGCAAACTTCAAGAGGGTTACCCAACGTGGGGTAGTTTAGAAAAGA
ACGGCGAGATTTGGGCGGGCCGTGAAATCGACGAAACTGGCTCGTCGGATTGGACTCAG
AAGTCGGGTGGGGGGCTGTTGTTTATTAAGGGCAAAATGATTGGCCGCTACAATTTCCA
CAATGAAAAGTCTCTGCGCATTACGAAAAAGATTGACAAGGTGCTTTCCAATAGCAACT
TCGTCCGCATCGCCTGGCGCGATATTTCGCGCCCAAGCCAAAAACGCCGCATGATCGCA
ACTATCATTCCGCCTAACTCGTTGGCTGGTAACTCATTGGGTGTAGTATACTACAAATC
CGGGTCCCAGGATTCCCTGTTTTCCTTGCTTGGAATTATTAACTCTCTGTGCTTCGAAT
TTCAATTGCGCTCCTTTTTGGCTACTGGGCATGTTAGTCTGTCTGCTCTTCGTAAAACC
GCGATCCCTAGCGAAAAGATCTTACTGCAACACAGTGAGTTGAAACAGCTGGTAATTAG
CTGCATTGAGGGGTGCTGCGATGCGGAATTAAAGATTGAAGCATATGTGGCGAAGAACA
TTTACAAACTTGACCTGAATGAGTTCAACAAATTGCTTAGTAGCTTCGACAAAATCGAG
TTGGCAGAAAAAGAGTCTTTGTTACGCATCTTCCAGCACTACGATTAA

M1.*Eco*31I (POC1480-KPL18)

ATGGAGGAGATCTTCTATATGAAACACATCCATTTAATCAACAGCCTGTCGCTGGACGA
AACTACTAAGTTCACGAAAAAAGCTACCGGTAAATACTACACCGACCCCAAAATCGCGC
TGTTAATGATCGAGAAGTTACTTCCGCTGATTAACTCCTGTGATAAAAAGAGCTATAAC
GTTGCTGATCCCTTTTCAGGTGACGGTCGTCTGATCACTCTTTTGATCAAGCAGTGGAT
GATTAACGGCTTCCCCGATGTCGAATGGAACGTCTACCTGTTTGACATTGAGAACACCG
GTCTGACTTACGCCAAAAACGCTCTGTCGGAATTGAAGTTAGCCGGCGCTAATATCAAT
ATTACAATTAAGAACTCGGATGTATTTTACGAATTTAAGAAGTATGTAGACTACTTCGA
CTGTGTGATCACAAATCCTCCCTGGGAGAACATTAAACCTGATTCTCGTGAGCTTGATT
TTTTCGAACCAAGCATGAAATCCATGTATATTGACAGCCTTCGTGAATTTGATGATTAC
CTTTCACGTGTGCTTCCCTATAGTCAGCCGAAGCGCAAGTTCGCTGGCTGGGGTACGAA
CTTAAGTCGTGTTGGTGCAGAGTTATCTCTTGAGATTTGCAACAAAAATGGGTTAGTAG
CGATCGTCATGCCCGCCAGCTTTTTTGCGGACGAGCAATCCTATATTTTACGTGAGAAG
TTTTTCAATTCCGGACGTATCGACTGTATTAACTATTATCCAGCCGAAGCAAAACTTTT
CGGTGGAGCAGATGTGAGCTCCTGCTCCTTCATTTTTAACAAGGGAGAATCCTTGAATG

ATAATATTCAATTAAGCGTCTACGATAAAAATTTGAACATCAAGTCATTGGGTTTTTTC
GATTTGTCAAGCATCGATTCCCAATATCTGTCGATCCCCGTGTCTCAAGGCGTGCATGC
GGTCCATCTTTTGCGCAAACTTCAAGAGGGTTACCCAACGTGGGGTAGTTTAGAAAAGA
ACGGCGAGATTTGGGCGGGCCGTGAAATCGACGAAACTGGCTCGTCGGATTGGACTCAG
AAGTCGGGTGGGGGGCTGTTGTTTATTAAGGGCAAAATGATTGGCCGCTACAATTTCCA
CAATGAAAAGTCTCTGCGCATTACGAAAAAGATTGACAAGGTGCTTTCCAATAGCAACT
TCGTCCGCATCGCCTGGCGCGATATTTCGCGCCCAAGCCAAAAACGCCGCATGATCGCA
ACTATCATTCCGCCTAACTCGTTGGCTGGTAACTCATTGGGTGTAGTATACTACAAATC
CGGGTCCCAGGATTCCCTGTTTTCCTTGCTTGGAATTATTAACTCTCTGTGCTTCGAAT
TTCAATTGCGCTCCTTTTTGGCTACTGGGCATGTTAGTCTGTCTGCTCTTCGTAAAACC
GCGATCCCTAGCGAAAAGATCTTACTGCAACACAGTGAGTTGAAACAGCTGGTAATTAG
CTGCATTGAGGGGTGCTGCGATGCGGAATTAAAGATTGAAGCATATGTGGCGAAGAACA
TTTACAAACTTGACCTGAATGAGTTCAACAAATTGCTTAGTAGCTTCGACAAAATCGAG
TTGGCAGAAAAAGAGTCTTTGTTACGCATCTTCCAGCACTACGATTAA

M.*Xmn*I (POC1481-KPL19)

ATGCGTGATCTTGCTAGTACTTATCGTCGTGCCCACAATACCATGCGCAATTTAGATGG
ACTTCAACCGCAAGAGGCGTTTGAGGAACTGCTGAAGTTCTTATTCTTAAAGCAAATGT
CCGAGGAAGATGGTATCGCCCCTACAACGGGAAGCGCGATTCGCAAACGCTTTGCATTC
CACCTGTCCCGCCACAGTAGTTGGAGCACGAGTTTATGGCGCGATCGTGACTTTCACCT
TTCAGACCAGTGCTTAGAACAATTGAACGCTCTGTTCAGCGGGATTAATTTCACACAGA
TCGATTATGATATTCGTTCGGCGGCTCTTCGTGAGTTTTTGACACCAGAAGTACGCAAG
GGACTTGGGATCTTTCTGACTCCTGATGAAGTCGTACGTGAGGTAGTCTCGTTTGTAGA
CCCGCCCAGCTCTGCTAAATGCCTGGATCCTGCGTGCGGATCGGGGACCTTTTTAATTG
AAGTGATCAAGAAGTGGCGTAAGGAAAATGCGCAGAAGATCAGTGTCTGGGGGGCGGAT
AAGAACCCTCGTATGCTTTTAATTGGAGAACTTAACCTTGGTCACTTCCCTGGCTTAAC
GTTTAATCGCGCCCTGATGGACTCGCTTGTCGAACCGGGCAAACGCCATTCTAAACCCT
GGTGTCGCTACGGCTACTTCGATTTTATTCTTACCAATCCTCCATTCGGCGTAACAGTC
GAAGCCTCGGGTGCGGCTTACTCGGGGTACGATATCGCGTTTACAGCAAATGGAGAGCC
ACGCGCCCGCCAAAGCTCAGAATGGTTATTTGTTGAACAATCGCTTCGCTGGCTGAAGC
CTGGCGGAACTTTGGCTGTAGTGTTGCCTCGCAGCGTTCTGACGAACCCATCGTCAGCG
TACGAACGTAGCTTGTTGGCGAAACTTGGCTACCTGAAGGCTGTCATTCAATTGCCACC
GGAAACGTTCTTGGTGACCGGGGCGCAGACAAACACAGTGGTAGCCTTCATTGAGAAAT
ACGCTTCTGATAAGGACCGTGAAAAGAAAGTAGGAGTAGTCCAGGCGACCTTATCGAAT
GTCGGATACGACAGCACAGGACGCCCGCGTCAAGGGTCGCAACTTGATGGTTTAGCCAA
GCATATGCGCGACCCAAAAGGTTCACCCTGTGATTATGTTTCTGTCCTGGCTCCCCAGA
AGGCTGACCGCACCTTCGAGGCACTGTCTGCGAAGTCCGCGTCAAAACGTCGCTCGAAC
CTTGGTGTTCCTTTAGGTGATTTGGCCAGCGAGATTCGCACCGGACGTACGCCACCTCG
TGCAGCATATAGTGAGAATGGCGGTCTTTTTCTTATCAAGGTAGGCAACTTGACTGGGA
GTGGAATTAACTGGATTGCTCGCGACCGTAACTTTATTGATACGTCAACGATGACTAGT
CGCAAACTTGGGGGCATTCAATTGGTCAAAAACGGCGACATCGTCTTAACTAGTAGCGC
ACACAGTCCAGTCTATATTGCAAAGAAAGTTGACATTATCACCAAAGTTCCAGAATGGG
TAGGCGGCCGTGCATCATTTGTGGGAGAAGTAATGTTGGTGCGCCCAAAGCAAGAACGC
ATTGCACCTATGTTACTGCTGGCCTACTTACGCCAACCGACAGTGGCCTCTCAGATCCA
GGAAATGGTGCGCGGTCAGACGGCGCACTTACACGCAGATGATCTTGCGGCTCTGATGG
TCCCTACATCAGTGCTGGATGCTTCCGATGACTGGCGTGAGGTGCAGGAATTGCTGGTC
GAAGAGGTAGCCTTAAATGACCGCCTGAACGAGATCGCACGTTTACAACAGAGTATCGC
GAACAAGTTAAGCGATGGTTTGCGCGCCGGGTAA

M.*Xmn*I (POC1482-KPL20)

ATGCGTGATCTTGCTAGTACTTATCGTCGTGCCCACAATACCATGCGCAATTTAGATGG
ACTTCAACCGCAAGAGGCGTTTGAGGAACTGCTGAAGTTCTTATTCTTAAAGCAAATGT
CCGAGGAAGATGGTATCGCCCCTACAACGGGAAGCGCGATTCGCAAACGCTTTGCATTC
CACCTGTCCCGCCACAGTAGTTGGAGCACGAGTTTATGGCGCGATCGTGACTTTCACCT
TTCAGACCAGTGCTTAGAACAATTGAACGCTCTGTTCAGCGGGATTAATTTCACACAGA
TCGATTATGATATTCGTTCGGCGGCTCTTCGTGAGTTTTTGACACCAGAAGTACGCAAG
GGACTTGGGATCTTTCTGACTCCTGATGAAGTCGTACGTGAGGTAGTCTCGTTTGTAGA
CCCGCCCAGCTCTGCTAAATGCCTGGATCCTGCGTGCGGATCGGGGACCTTTTTAATTG
AAGTGATCAAGAAGTGGCGTAAGGAAAATGCGCAGAAGATCAGTGTCTGGGGGGCGGAT
AAGAACCCTCGTATGCTTTTAATTGGAGAACTTAACCTTGGTCACTTCCCTGGCTTAAC
GTTTAATCGCGCCCTGATGGACTCGCTTGTCGAACCGGGCAAACGCCATTCTAAACCCT
GGTGTCGCTACGGCTACTTCGATTTTATTCTTACCAATCCTCCATTCGGCGTAACAGTC
GAAGCCTCGGGTGCGGCTTACTCGGGGTACGATATCGCGTTTACAGCAAATGGAGAGCC
ACGCGCCCGCCAAAGCTCAGAATGGTTATTTGTTGAACAATCGCTTCGCTGGCTGAAGC
CTGGCGGAACTTTGGCTGTAGTGTTGCCTCGCAGCGTTCTGACGAACCCATCGTCAGCG
TACGAACGTAGCTTGTTGGCGAAACTTGGCTACCTGAAGGCTGTCATTCAATTGCCACC
GGAAACGTTCTTGGTGACCGGGGCGCAGACAAACACAGTGGTAGCCTTCATTGAGAAAT
ACGCTTCTGATAAGGACCGTGAAAAGAAAGTAGGAGTAGTCCAGGCGACCTTATCGAAT
GTCGGATACGACAGCACAGGACGCCCGCGTCAAGGGTCGCAACTTGATGGTTTAGCCAA
GCATATGCGCGACCCAAAAGGTTCACCCTGTGATTATGTTTCTGTCCTGGCTCCCCAGA
AGGCTGACCGCACCTTCGAGGCACTGTCTGCGAAGTCCGCGTCAAAACGTCGCTCGAAC
CTTGGTGTTCCTTTAGGTGATTTGGCCAGCGAGATTCGCACCGGACGTACGCCACCTCG
TGCAGCATATAGTGAGAATGGCGGTCTTTTTCTTATCAAGGTAGGCAACTTGACTGGGA
GTGGAATTAACTGGATTGCTCGCGACCGTAACTTTATTGATACGTCAACGATGACTAGT
CGCAAACTTGGGGGCATTCAATTGGTCAAAAACGGCGACATCGTCTTAACTAGTAGCGC
ACACAGTCCAGTCTATATTGCAAAGAAAGTTGACATTATCACCAAAGTTCCAGAATGGG
TAGGCGGCCGTGCATCATTTGTGGGAGAAGTAATGTTGGTGCGCCCAAAGCAAGAACGC
ATTGCACCTATGTTACTGCTGGCCTACTTACGCCAACCGACAGTGGCCTCTCAGATCCA
GGAAATGGTGCGCGGTCAGACGGCGCACTTACACGCAGATGATCTTGCGGCTCTGATGG
TCCCTACATCAGTGCTGGATGCTTCCGATGACTGGCGTGAGGTGCAGGAATTGCTGGTC
GAAGAGGTAGCCTTAAATGACCGCCTGAACGAGATCGCACGTTTACAACAGAGTATCGC
GAACAAGTTAAGCGATGGTTTGCGCGCCGGGTAA

M1.*Hpy*AII (POC1483-KPL21)

ATGATTCTGAATAAGATCTACATTGAGGATGTGTTCACCTTCCTGGACAAGCTTGAGGA
TAAGTCGGTGGATCTTGCTATCATCGATCCACCGTATAATCTTAAAATTGCATCATGGG
ACTCCTTTAAAAACGATGAAGAGTTCTTAACTTTTAGCTATGCATGGATCGATAAGATG
CTTCCAAAGCTGAAAGATACAGGATCGTTCTATATTTTCAATACCCCTTTCAACTGTGC
CCTTTTTTTAGCCTACTTACATCATAAAAAAGTGCATTTCTTGAACTTTATTACATGGG
TGAAAAAAGACGGGTTTGCCAATGCCAAGAAACGTTACAACCATGCTCAAGAATCGATT
CTGTTTTACTCAATGCACAAGAAAAATTACACATTTAATGCGGACGAAATTCGTATTGC
GTATGAGAGTGCGGAACGTATTAAACATGCTCAGTCCAAGGGTATCCTTAAAAATAATA
AACGTTGGTTCCCCAATCCCAAGGGAAAACTGTGCCTGGACGTGTGGGAAATTACTTCG
CAGCGCCACGTAGAGAAAGAAAAAGGAAAAATTCTGAAACCGAAACACCCATCGATCAA
ACCGAAGGCACTGATTGAGCGCATGATCAAGGCATCATCACACAAAAATGACCTTATCT
TGGACTTGTTTTCGGGCTCCGGGATGACTTCTTTAGTGGCAAAAAGCTTGGAGCGTAAT
TTTATCGGCTGTGAGTCCCACGCAGAGTATGTGCACGGATCACTGGAAATGTTCCGCTA
TAACGAGTGTGAATAA

M1.*Hpy*AII (POC1484-KPL22)

ATGATTCTGAATAAGATCTACATTGAGGATGTGTTCACCTTCCTGGACAAGCTTGAGGA
TAAGTCGGTGGATCTTGCTATCATCGATCCACCGTATAATCTTAAAATTGCATCATGGG
ACTCCTTTAAAAACGATGAAGAGTTCTTAACTTTTAGCTATGCATGGATCGATAAGATG
CTTCCAAAGCTGAAAGATACAGGATCGTTCTATATTTTCAATACCCCTTTCAACTGTGC
CCTTTTTTTAGCCTACTTACATCATAAAAAAGTGCATTTCTTGAACTTTATTACATGGG
TGAAAAAAGACGGGTTTGCCAATGCCAAGAAACGTTACAACCATGCTCAAGAATCGATT
CTGTTTTACTCAATGCACAAGAAAAATTACACATTTAATGCGGACGAAATTCGTATTGC
GTATGAGAGTGCGGAACGTATTAAACATGCTCAGTCCAAGGGTATCCTTAAAAATAATA
AACGTTGGTTCCCCAATCCCAAGGGAAAACTGTGCCTGGACGTGTGGGAAATTACTTCG
CAGCGCCACGTAGAGAAAGAAAAAGGAAAAATTCTGAAACCGAAACACCCATCGATCAA
ACCGAAGGCACTGATTGAGCGCATGATCAAGGCATCATCACACAAAAATGACCTTATCT
TGGACTTGTTTTCGGGCTCCGGGATGACTTCTTTAGTGGCAAAAAGCTTGGAGCGTAAT
TTTATCGGCTGTGAGTCCCACGCAGAGTATGTGCACGGATCACTGGAAATGTTCCGCTA
TAACGAGTGTGAATAA

M2.*Hpy*AII (POC1485-KPL23)

ATGAACATTAATAAGGTGTTCTACCATTCATCGACAAATATGAACGAAGTACCCGATAA
TTCGGTTGATCTTATCATCACCTCGCCTCCTTATTTTAATATCAAAGACTATGCCAAAA
ATGGCACACAAGACTTGCAACATTCTGCTCAGCATGTGGAAGATTTGGGTGCTTTAGAG
AAATACGAGGATTATCTGCTGGGTCTGTTGAAGGTTTGGTTGGAATGTTATCGTGCTCT
GAAGCCAAACGGTAAACTGTGCATCAACGTACCATTGATGCCTATGTTGAAGAAGGTAT
TGAATACGCATTATAATCGTCACATTTTCGACTTACACGCGGATATTCAACACTCAATT
CTTCATGATTTAAACAACATGTTGAAAAATAAGCCGAAGATGTTTTTGTTGGATGTGTA
TATTTGGAAGCGTGCCAATCCAACGAAACGCTTGATGTTTGGATCCTACCCGTACCCCC
GCAATTTTTACGCACAGAATACTATTGAGTTCATCGGCGTTTTCGTGAAGGATGGTAAA
CCAAAACAACCTACTGAAGAGCAAAAAGAGCAAAGTCAATTAACCCAAGAGGAATGGGT
AGAGTTCACTAAGCAAATCTGGGAGATTCCTATTCCTAATAAGAACGACATCGCTTTTG
GAAAACACGCAGCCCTGATGCCCGCAGAACTGGCCCGCCGCTTGATTCGTCTTTATTCT
TGTGTCGGCGACGTCGTCTTAGACCCATTTTCGGGCTCCGGCACGACGCTTCGCGAAGC
CAAGCTTCTTAAACGCAACTTCATTGGGTACGAGCTGTATGAAAACTATAAACCGCTGA
TCGAGCAGAAATTGGGCAATTTATTTGACTTTGAATAA

M2.*Hpy*AII (POC1486-KPL24)

ATGAACATTAATAAGGTGTTCTACCATTCATCGACAAATATGAACGAAGTACCCGATAA
TTCGGTTGATCTTATCATCACCTCGCCTCCTTATTTTAATATCAAAGACTATGCCAAAA
ATGGCACACAAGACTTGCAACATTCTGCTCAGCATGTGGAAGATTTGGGTGCTTTAGAG
AAATACGAGGATTATCTGCTGGGTCTGTTGAAGGTTTGGTTGGAATGTTATCGTGCTCT
GAAGCCAAACGGTAAACTGTGCATCAACGTACCATTGATGCCTATGTTGAAGAAGGTAT
TGAATACGCATTATAATCGTCACATTTTCGACTTACACGCGGATATTCAACACTCAATT
CTTCATGATTTAAACAACATGTTGAAAAATAAGCCGAAGATGTTTTTGTTGGATGTGTA
TATTTGGAAGCGTGCCAATCCAACGAAACGCTTGATGTTTGGATCCTACCCGTACCCCC
GCAATTTTTACGCACAGAATACTATTGAGTTCATCGGCGTTTTCGTGAAGGATGGTAAA
CCAAAACAACCTACTGAAGAGCAAAAAGAGCAAAGTCAATTAACCCAAGAGGAATGGGT
AGAGTTCACTAAGCAAATCTGGGAGATTCCTATTCCTAATAAGAACGACATCGCTTTTG
GAAAACACGCAGCCCTGATGCCCGCAGAACTGGCCCGCCGCTTGATTCGTCTTTATTCT
TGTGTCGGCGACGTCGTCTTAGACCCATTTTCGGGCTCCGGCACGACGCTTCGCGAAGC

CAAGCTTCTTAAACGCAACTTCATTGGGTACGAGCTGTATGAAAACTATAAACCGCTGA
TCGAGCAGAAATTGGGCAATTTATTTGACTTTGAATAA

M1.*Mbo*II (POC1487-KPL25)

ATGGTAGAGAACATGTTAGAGATCAATAAAATCCACCAAATGAATTGCTTCGATTTTTT
GGACCAGGTTGAGAACAAATCTGTTCAGTTGGCGGTCATTGATCCTCCATATAACCTGA
GTAAGGCGGATTGGGATTCTTTTGATTCACATAACGAGTTCCTGGCATTTACGTACCGC
TGGATCGATAAGGTTCTGGACAAGTTGGACAAAGACGGGTCGCTTTACATCTTTAACAC
TCCGTTTAATTGCGCTTTTATTTGCCAGTACCTTGTTAGTAAGGGAATGATCTTTCAAA
ACTGGATTACGTGGGATAAACGTGACGGAATGGGGTCAGCGAAACGCCGTTTTAGCACA
GGCCAAGAGACTATCCTGTTCTTTAGCAAATCCAAAAACCATACATTTAACTACGATGA
GGTCCGCGTCCCTTACGAGTCAACCGACCGTATCAAGCACGCCAGTGAGAAGGGAATCC
TGAAAAACGGAAAACGTTGGTTCCCGAACCCAAACGGCCGTCTGTGCGGAGAGGTGTGG
CATTTCAGTAGTCAGCGTCACAAGGAGAAGGTTAATGGTAAAACCGTTAAACTTACGCA
CATTACCCCTAAGCCCCGTGACCTGATTGAACGTATCATTCGCGCCAGTTCTAATCCTA
ATGACCTTGTCTTAGACTGTTTCATGGGGTCTGGCACAACTGCCATTGTAGCCAAGAAG
TTGGGGCGTAACTTCATCGGATGCGATATGAACGCAGAATATGTAAATCAGGCAAATTT
TGTGCTGAACCAGTTGGAGATTAACTAA

M1.*Mbo*II (POC1488-KPL26)

ATGGTAGAGAACATGTTAGAGATCAATAAAATCCACCAAATGAATTGCTTCGATTTTTT
GGACCAGGTTGAGAACAAATCTGTTCAGTTGGCGGTCATTGATCCTCCATATAACCTGA
GTAAGGCGGATTGGGATTCTTTTGATTCACATAACGAGTTCCTGGCATTTACGTACCGC
TGGATCGATAAGGTTCTGGACAAGTTGGACAAAGACGGGTCGCTTTACATCTTTAACAC
TCCGTTTAATTGCGCTTTTATTTGCCAGTACCTTGTTAGTAAGGGAATGATCTTTCAAA
ACTGGATTACGTGGGATAAACGTGACGGAATGGGGTCAGCGAAACGCCGTTTTAGCACA
GGCCAAGAGACTATCCTGTTCTTTAGCAAATCCAAAAACCATACATTTAACTACGATGA
GGTCCGCGTCCCTTACGAGTCAACCGACCGTATCAAGCACGCCAGTGAGAAGGGAATCC
TGAAAAACGGAAAACGTTGGTTCCCGAACCCAAACGGCCGTCTGTGCGGAGAGGTGTGG
CATTTCAGTAGTCAGCGTCACAAGGAGAAGGTTAATGGTAAAACCGTTAAACTTACGCA
CATTACCCCTAAGCCCCGTGACCTGATTGAACGTATCATTCGCGCCAGTTCTAATCCTA
ATGACCTTGTCTTAGACTGTTTCATGGGGTCTGGCACAACTGCCATTGTAGCCAAGAAG
TTGGGGCGTAACTTCATCGGATGCGATATGAACGCAGAATATGTAAATCAGGCAAATTT
TGTGCTGAACCAGTTGGAGATTAACTAA

Protein sequences of methylases with His6-tag

M2.*Eco*31I (POC1463-KPL01), N-terminal His6-tag

MGSSHHHHHHSSGLVPRGSHMTKSETFMIPNHKAAKLSELDMMIVNSVPPGGNWKNIPL
DVPSKRIEQIRDSYAQGKGSRSTYYGRLLPDMPAYTINTYFNRPGNGCHIHYEQDRVLS
QREAARLQSFPDDFIFFGGQTAINTQIGNAVPPFLAFLIAKEIEKAIGNTGYYIDLFSG
AGGLGLGFKWAGWTPLLANDIEEKYLQTYSNNVHKEVLCGSISDNETFSKIADKISGFK
KLYFDKQLWILGGPPCQGFSTAGNARTMDDPRNSLFMHYKSLLNEIKPNGFIFENVAGL
LNMEKGKVFERVKEEFSSTMKTMNGWILNSEHYAIPQRRKRVILVGSNDPLFSIEPPQK
LTEDKESWVSVKDALSDLPPLQHGEDGSGKYYIHHPENDYQLFMRGNITPSEYYERNIK
PSL

M2.*Eco*31I (POC1464-KPL02), C-terminal His6-tag

MTKSETFMIPNHKAAKLSELDMMIVNSVPPGGNWKNIPLDVPSKRIEQIRDSYAQGKGS
RSTYYGRLLPDMPAYTINTYFNRPGNGCHIHYEQDRVLSQREAARLQSFPDDFIFFGGQ
TAINTQIGNAVPPFLAFLIAKEIEKAIGNTGYYIDLFSGAGGLGLGFKWAGWTPLLAND
IEEKYLQTYSNNVHKEVLCGSISDNETFSKIADKISGFKKLYFDKQLWILGGPPCQGFS
TAGNARTMDDPRNSLFMHYKSLLNEIKPNGFIFENVAGLLNMEKGKVFERVKEEFSSTM
KTMNGWILNSEHYAIPQRRKRVILVGSNDPLFSIEPPQKLTEDKESWVSVKDALSDLPP
LQHGEDGSGKYYIHHPENDYQLFMRGNITPSEYYERNIKPSLKLAAALEHHHHHH

M2.*Eco*31I_2 (POC1465-KPL03), truncated M2.*Eco*31I, N-terminal His6-tag

MGSSHHHHHHSSGLVPRGSHMIPNHKAAKLSELDMMIVNSVPPGGNWKNIPLDVPSKRI
EQIRDSYAQGKGSRSTYYGRLLPDMPAYTINTYFNRPGNGCHIHYEQDRVLSQREAARL
QSFPDDFIFFGGQTAINTQIGNAVPPFLAFLIAKEIEKAIGNTGYYIDLFSGAGGLGLG
FKWAGWTPLLANDIEEKYLQTYSNNVHKEVLCGSISDNETFSKIADKISGFKKLYFDKQ
LWILGGPPCQGFSTAGNARTMDDPRNSLFMHYKSLLNEIKPNGFIFENVAGLLNMEKGK
VFERVKEEFSSTMKTMNGWILNSEHYAIPQRRKRVILVGSNDPLFSIEPPQKLTEDKES
WVSVKDALSDLPPLQHGEDGSGKYYIHHPENDYQLFMRGNITPSEYYERNIKPSL

M2.*Eco*31I_2 (POC1466-KPL04), truncated M2.*Eco*31, C-terminal His6-tag

MIPNHKAAKLSELDMMIVNSVPPGGNWKNIPLDVPSKRIEQIRDSYAQGKGSRSTYYGR
LLPDMPAYTINTYFNRPGNGCHIHYEQDRVLSQREAARLQSFPDDFIFFGGQTAINTQI
GNAVPPFLAFLIAKEIEKAIGNTGYYIDLFSGAGGLGLGFKWAGWTPLLANDIEEKYLQ
TYSNNVHKEVLCGSISDNETFSKIADKISGFKKLYFDKQLWILGGPPCQGFSTAGNART
MDDPRNSLFMHYKSLLNEIKPNGFIFENVAGLLNMEKGKVFERVKEEFSSTMKTMNGWI
LNSEHYAIPQRRKRVILVGSNDPLFSIEPPQKLTEDKESWVSVKDALSDLPPLQHGEDG
SGKYYIHHPENDYQLFMRGNITPSEYYERNIKPSLKLAAALEHHHHHH

M2.*Bsa*I (POC1467-KPL05), N-terminal His6-tag

MGSSHHHHHHSSGLVPRGSHMIPNHVSSKLSELDMLIIKHVPPGGNWKDIPEWVPSKRL
EQIRKSYAEGKGSRSTYYGRLLPDMPSYTINTYFNRPGNGCHIHYEQDRTLSQREAARL
QSFPDDFIFYGSKTAINNQIGNAVPPLLAYQIAKAFPFKGQFVDLFSGAGGLSLGFLWA
GWKPIIANDIDKWALTTYMNNIHNEVVLGDIRDEKVSETIIQKCLIAKKSNPDRPLFVL

GGPPCQGFSTAGKKRSIVDERNWLFESYVSILKEVKPDGFIFENVTGLLSMEKGAFFEM
VKSELSKTVSNLFVYKLNSVDYGVPQRRNRVVIIGDSTGTKNSEPPIPITSLKGEKTLF
DALSSAISVKEALSDLPLLSPNEDGSWKNYVCEPQNIYQSFMRKKITAQQYIEMLSSLA
II

M2.*Bsa*I (POC1468-KPL06), C-terminal His6-tag

MIPNHVSSKLSELDMLIIKHVPPGGNWKDIPEWVPSKRLEQIRKSYAEGKGSRSTYYGR
LLPDMPSYTINTYFNRPGNGCHIHYEQDRTLSQREAARLQSFPDDFIFYGSKTAINNQI
GNAVPPLLAYQIAKAFPFKGQFVDLFSGAGGLSLGFLWAGWKPIIANDIDKWALTTYMN
NIHNEVVLGDIRDEKVSETIIQKCLIAKKSNPDRPLFVLGGPPCQGFSTAGKKRSIVDE
RNWLFESYVSILKEVKPDGFIFENVTGLLSMEKGAFFEMVKSELSKTVSNLFVYKLNSV
DYGVPQRRNRVVIIGDSTGTKNSEPPIPITSLKGEKTLFDALSSAISVKEALSDLPLLS
PNEDGSWKNYVCEPQNIYQSFMRKKITAQQYIEMLSSLAIIKLAAALEHHHHHH

M2.*Bsa*I_2 (POC1469-KPL07), truncated M2.*Bsa*I, N-terminal His6-tag

MGSSHHHHHHSSGLVPRGSHMLIIKHVPPGGNWKDIPEWVPSKRLEQIRKSYAEGKGSR
STYYGRLLPDMPSYTINTYFNRPGNGCHIHYEQDRTLSQREAARLQSFPDDFIFYGSKT
AINNQIGNAVPPLLAYQIAKAFPFKGQFVDLFSGAGGLSLGFLWAGWKPIIANDIDKWA
LTTYMNNIHNEVVLGDIRDEKVSETIIQKCLIAKKSNPDRPLFVLGGPPCQGFSTAGKK
RSIVDERNWLFESYVSILKEVKPDGFIFENVTGLLSMEKGAFFEMVKSELSKTVSNLFV
YKLNSVDYGVPQRRNRVVIIGDSTGTKNSEPPIPITSLKGEKTLFDALSSAISVKEALS
DLPLLSPNEDGSWKNYVCEPQNIYQSFMRKKITAQQYIEMLSSLAII

M2.*Bsa*I_2 (POC1470-KPL03), truncated M2.*Bsa*I, C-terminal His6-tag

MLIIKHVPPGGNWKDIPEWVPSKRLEQIRKSYAEGKGSRSTYYGRLLPDMPSYTINTYF
NRPGNGCHIHYEQDRTLSQREAARLQSFPDDFIFYGSKTAINNQIGNAVPPLLAYQIAK
AFPFKGQFVDLFSGAGGLSLGFLWAGWKPIIANDIDKWALTTYMNNIHNEVVLGDIRDE
KVSETIIQKCLIAKKSNPDRPLFVLGGPPCQGFSTAGKKRSIVDERNWLFESYVSILKE
VKPDGFIFENVTGLLSMEKGAFFEMVKSELSKTVSNLFVYKLNSVDYGVPQRRNRVVII
GDSTGTKNSEPPIPITSLKGEKTLFDALSSAISVKEALSDLPLLSPNEDGSWKNYVCEP
QNIYQSFMRKKITAQQYIEMLSSLAIIKLAAALEHHHHHH

M.*Osp*807II (POC1471-KPL09), N-terminal His6-tag

MGSSHHHHHHSSGLVPRGSHMAKKDTNLKLSHLYKGNVSEVYGRWPSPDLIVSDGAYGV
RGFRGDTVDAAGLTDWYKPHVLAWAKAAKPSTSLWFWNTEVGWATVHPLLLSTGWEYVQ
LAVWDKGLAHIAGNVNGKTIRQLPVVTEVAALYRRTVYLETGDGLTLNAKSWLRAEWRR
SGLSLSKSNEACGVKNAATRKYLTADWLWYWPPGDAVQKMAEYCMQYGKKTSWPYFSLD
GKTMISAHDWDSLRTTWNHRNGVTNVWSRPPLADSERLKGTMERSAPRTYKPTKQSAAH
LNQKPLDLMLTQVAAASNVGDTVWEPFGGLCSASVASSLLGRRSYAAEIDDTFYKLAAA
RLNEANAYFESNGVYEFKEGE

M.*Osp*807II (POC1472-KPL10), C-terminal His6-tag

MAKKDTNLKLSHLYKGNVSEVYGRWPSPDLIVSDGAYGVRGFRGDTVDAAGLTDWYKPH
VLAWAKAAKPSTSLWFWNTEVGWATVHPLLLSTGWEYVQLAVWDKGLAHIAGNVNGKTI

RQLPVVTEVAALYRRTVYLETGDGLTLNAKSWLRAEWRRSGLSLSKSNEACGVKNAATR
KYLTADWLWYWPPGDAVQKMAEYCMQYGKKTSWPYFSLDGKTMISAHDWDSLRTTWNHR
NGVTNVWSRPPLADSERLKGTMERSAPRTYKPTKQSAAHLNQKPLDLMLTQVAAASNVG
DTVWEPFGGLCSASVASSLLGRRSYAAEIDDTFYKLAAARLNEANAYFESNGVYEFKEG
ELEHHHHHH

M2.*Nme*MC58II (POC1473-KPL11), N-terminal His6-tag

MGSSHHHHHHSSGLVPRGSHMITISNEDNMILMSRYPDKYFDLAIVDPPYGILNKTKRG
GDYKFNMNEYSQWDIKPDQTYFNELFRVSKNQIIWGGNYFGELWLRSEYNKGFIIWDKN
QPETLNNFSMAEMAWSSFDRPSKIFRFSVRKNRNKTHPTQKPVELYQWLLKMYAKQGDK
ILDTHLGSGTLAIACCIAQFDLTACEINSDYYQQSIEKIKNNLPEARISFGHPGYCIIE

M2.*Nme*MC58II (POC1474-KPL12), C-terminal His6-tag

MITISNEDNMILMSRYPDKYFDLAIVDPPYGILNKTKRGGDYKFNMNEYSQWDIKPDQT
YFNELFRVSKNQIIWGGNYFGELWLRSEYNKGFIIWDKNQPETLNNFSMAEMAWSSFDR
PSKIFRFSVRKNRNKTHPTQKPVELYQWLLKMYAKQGDKILDTHLGSGTLAIACCIAQF
DLTACEINSDYYQQSIEKIKNNLPEARISFGHPGYCIIEKLAAALEHHHHHH

M.*Sen*0738I (POC1475-KPL13), N-terminal His6-tag

MGSSHHHHHHSSGLVPRGSHMSISSAIKSLQDIMRKDAGVDGDAQRLGQLSWLLFLKIF
DAQEQALEIEQEKYRLPMPERYLWRNWAADNEGITGDKLLAFVNDDLFPTLKDLPAQID
INPRGYVVKQAFSDAYNYMKNGTLLRQVINKLNEIDFTRASERHLFGDIYEQILRDLQA
AGNAGEFYTPRAVTRFMVERVDPKLGESIMDPACGTGGFLACAFDHVKNHYAHTVTDHQ
ILQKQIHGVEKKQLPHLLCTTNMLLHGIEVPVQIRHDNTLNKPLSSWDEQMDVIITNPP
FGGTEEDGIEKNFPSDMQTRETADLFLQLIIEVLAKNGRAAVVLPDGTLFGEGVKTKIK
KLLTEECNLHTIVRLPNGVFNPYTGIKTNLLFFTKGQPTKEIWFYEHPYPAGVKNYSKT
KPMKFEEFQAEIDWWGNEADGFASRVENEQAWKVSIDEVIARNFNLDIKNPHQAETVSH
DPDELLAQYAKQQEAIQTLRHQLRDILGTALSGKEAN

M.*Sen*0738I (POC1476-KPL14), C-terminal His6-tag

MSISSAIKSLQDIMRKDAGVDGDAQRLGQLSWLLFLKIFDAQEQALEIEQEKYRLPMPE
RYLWRNWAADNEGITGDKLLAFVNDDLFPTLKDLPAQIDINPRGYVVKQAFSDAYNYMK
NGTLLRQVINKLNEIDFTRASERHLFGDIYEQILRDLQAAGNAGEFYTPRAVTRFMVER
VDPKLGESIMDPACGTGGFLACAFDHVKNHYAHTVTDHQILQKQIHGVEKKQLPHLLCT
TNMLLHGIEVPVQIRHDNTLNKPLSSWDEQMDVIITNPPFGGTEEDGIEKNFPSDMQTR
ETADLFLQLIIEVLAKNGRAAVVLPDGTLFGEGVKTKIKKLLTEECNLHTIVRLPNGVF
NPYTGIKTNLLFFTKGQPTKEIWFYEHPYPAGVKNYSKTKPMKFEEFQAEIDWWGNEAD
GFASRVENEQAWKVSIDEVIARNFNLDIKNPHQAETVSHDPDELLAQYAKQQEAIQTLR
HQLRDILGTALSGKEANLEHHHHHH

S.*Sen*0738I (POC1477-KPL15), N-terminal His6-tag

MGSSHHHHHHSSGLVPRGSHMAVEKLIVDHIDTWTTALQTRSTAGRGSSGKIDLYGIKK
LRELILELAVRGKLVPQDPNDKPASVLLERIATEKAELVKQGKIKKQKPLPEISEEEKP
FELPAGWEWARLNELAPMGIIDGDWIESKDQDPSGAYRLIQLADVGVGDFKDKSDRYIN

TSTFHRLNCHQLMEGDILIARLPNPIGRACIFPKLSQSAITVVDIATMRPSGNYSAEYI
ISAINSLTFRQQVESFGKGATRFRIATGHLKTLLLPIPPVQEQYSIFKKIKELMSLCDQ
LEQYSLTSLDAHQQLVETLLTTLTDSQNADELAENWARISEHFDTLFTTEVSIDALKQT
ILQLAVMGKLVPQDPNDEPASELLKRIAQEKAQLVKDGKMKKQKPLPPISDEEKPFELP
SGWEWCLFEDVVDIQSGITKGRNLANRKLISIPYLRVANVQRGYLDLSEVKEIDIPEEE
KDKYHVIKGDLLITEGGDWDTVGRTTVWCHDWYIANQNHVFKGRIIGQDIDPYWLETYM
NSPYARDYFASASKQTTNLASINKTQLRGCPVAIPPSSEAEKIMLKLNDFNELCEKLKL
QIQSAQQTQLHLADALTDAAIN

S.*Sen*0738I (POC1478-KPL16), C-terminal His6-tag

MAVEKLIVDHIDTWTTALQTRSTAGRGSSGKIDLYGIKKLRELILELAVRGKLVPQDPN
DKPASVLLERIATEKAELVKQGKIKKQKPLPEISEEEKPFELPAGWEWARLNELAPMGI
IDGDWIESKDQDPSGAYRLIQLADVGVGDFKDKSDRYINTSTFHRLNCHQLMEGDILIA
RLPNPIGRACIFPKLSQSAITVVDIATMRPSGNYSAEYIISAINSLTFRQQVESFGKGA
TRFRIATGHLKTLLLPIPPVQEQYSIFKKIKELMSLCDQLEQYSLTSLDAHQQLVETLL
TTLTDSQNADELAENWARISEHFDTLFTTEVSIDALKQTILQLAVMGKLVPQDPNDEPA
SELLKRIAQEKAQLVKDGKMKKQKPLPPISDEEKPFELPSGWEWCLFEDVVDIQSGITK
GRNLANRKLISIPYLRVANVQRGYLDLSEVKEIDIPEEEKDKYHVIKGDLLITEGGDWD
TVGRTTVWCHDWYIANQNHVFKGRIIGQDIDPYWLETYMNSPYARDYFASASKQTTNLA
SINKTQLRGCPVAIPPSSEAEKIMLKLNDFNELCEKLKLQIQSAQQTQLHLADALTDAA
INLEHHHHHH

M1.*Eco*31I (POC1479-KPL17), N-terminal His6-tag

MGSSHHHHHHSSGLVPRGSHMASMEEIFYMKHIHLINSLSLDETTKFTKKATGKYYTDP
KIALLMIEKLLPLINSCDKKSYNVADPFSGDGRLITLLIKQWMINGFPDVEWNVYLFDI
ENTGLTYAKNALSELKLAGANINITIKNSDVFYEFKKYVDYFDCVITNPPWENIKPDSR
ELDFFEPSMKSMYIDSLREFDDYLSRVLPYSQPKRKFAGWGTNLSRVGAELSLEICNKN
GLVAIVMPASFFADEQSYILREKFFNSGRIDCINYYPAEAKLFGGADVSSCSFIFNKGE
SLNDNIQLSVYDKNLNIKSLGFFDLSSIDSQYLSIPVSQGVHAVHLLRKLQEGYPTWGS
LEKNGEIWAGREIDETGSSDWTQKSGGGLLFIKGKMIGRYNFHNEKSLRITKKIDKVLS
NSNFVRIAWRDISRPSQKRRMIATIIPPNSLAGNSLGVVYYKSGSQDSLFSLLGIINSL
CFEFQLRSFLATGHVSLSALRKTAIPSEKILLQHSELKQLVISCIEGCCDAELKIEAYV
AKNIYKLDLNEFNKLLSSFDKIELAEKESLLRIFQHYD

M1.*Eco*31I (POC1480-KPL18), C-terminal His6-tag

MEEIFYMKHIHLINSLSLDETTKFTKKATGKYYTDPKIALLMIEKLLPLINSCDKKSYN
VADPFSGDGRLITLLIKQWMINGFPDVEWNVYLFDIENTGLTYAKNALSELKLAGANIN
ITIKNSDVFYEFKKYVDYFDCVITNPPWENIKPDSRELDFFEPSMKSMYIDSLREFDDY
LSRVLPYSQPKRKFAGWGTNLSRVGAELSLEICNKNGLVAIVMPASFFADEQSYILREK
FFNSGRIDCINYYPAEAKLFGGADVSSCSFIFNKGESLNDNIQLSVYDKNLNIKSLGFF
DLSSIDSQYLSIPVSQGVHAVHLLRKLQEGYPTWGSLEKNGEIWAGREIDETGSSDWTQ
KSGGGLLFIKGKMIGRYNFHNEKSLRITKKIDKVLSNSNFVRIAWRDISRPSQKRRMIA
TIIPPNSLAGNSLGVVYYKSGSQDSLFSLLGIINSLCFEFQLRSFLATGHVSLSALRKT

AIPSEKILLQHSELKQLVISCIEGCCDAELKIEAYVAKNIYKLDLNEFNKLLSSFDKIE
LAEKESLLRIFQHYDKLAAALEHHHHHH

M.*Xmn*I (POC1481-KPL19), N-terminal His6-tag

MGSSHHHHHHSSGLVPRGSHMASMRDLASTYRRAHNTMRNLDGLQPQEAFEELLKFLFL
KQMSEEDGIAPTTGSAIRKRFAFHLSRHSSWSTSLWRDRDFHLSDQCLEQLNALFSGIN
FTQIDYDIRSAALREFLTPEVRKGLGIFLTPDEVVREVVSFVDPPSSAKCLDPACGSGT
FLIEVIKKWRKENAQKISVWGADKNPRMLLIGELNLGHFPGLTFNRALMDSLVEPGKRH
SKPWCRYGYFDFILTNPPFGVTVEASGAAYSGYDIAFTANGEPRARQSSEWLFVEQSLR
WLKPGGTLAVVLPRSVLTNPSSAYERSLLAKLGYLKAVIQLPPETFLVTGAQTNTVVAF
IEKYASDKDREKKVGVVQATLSNVGYDSTGRPRQGSQLDGLAKHMRDPKGSPCDYVSVL
APQKADRTFEALSAKSASKRRSNLGVPLGDLASEIRTGRTPPRAAYSENGGLFLIKVGN
LTGSGINWIARDRNFIDTSTMTSRKLGGIQLVKNGDIVLTSSAHSPVYIAKKVDIITKV
PEWVGGRASFVGEVMLVRPKQERIAPMLLLAYLRQPTVASQIQEMVRGQTAHLHADDLA
ALMVPTSVLDASDDWREVQELLVEEVALNDRLNEIARLQQSIANKLSDGLRAG

M.*Xmn*I (POC1482-KPL20), C-terminal His6-tag

MRDLASTYRRAHNTMRNLDGLQPQEAFEELLKFLFLKQMSEEDGIAPTTGSAIRKRFAF
HLSRHSSWSTSLWRDRDFHLSDQCLEQLNALFSGINFTQIDYDIRSAALREFLTPEVRK
GLGIFLTPDEVVREVVSFVDPPSSAKCLDPACGSGTFLIEVIKKWRKENAQKISVWGAD
KNPRMLLIGELNLGHFPGLTFNRALMDSLVEPGKRHSKPWCRYGYFDFILTNPPFGVTV
EASGAAYSGYDIAFTANGEPRARQSSEWLFVEQSLRWLKPGGTLAVVLPRSVLTNPSSA
YERSLLAKLGYLKAVIQLPPETFLVTGAQTNTVVAFIEKYASDKDREKKVGVVQATLSN
VGYDSTGRPRQGSQLDGLAKHMRDPKGSPCDYVSVLAPQKADRTFEALSAKSASKRRSN
LGVPLGDLASEIRTGRTPPRAAYSENGGLFLIKVGNLTGSGINWIARDRNFIDTSTMTS
RKLGGIQLVKNGDIVLTSSAHSPVYIAKKVDIITKVPEWVGGRASFVGEVMLVRPKQER
IAPMLLLAYLRQPTVASQIQEMVRGQTAHLHADDLAALMVPTSVLDASDDWREVQELLV
EEVALNDRLNEIARLQQSIANKLSDGLRAGKLAAALEHHHHHH

M1.*Hpy*AII (POC1483-KPL21), N-terminal His6-tag

MGSSHHHHHHSSGLVPRGSHMILNKIYIEDVFTFLDKLEDKSVDLAIIDPPYNLKIASW
DSFKNDEEFLTFSYAWIDKMLPKLKDTGSFYIFNTPFNCALFLAYLHHKKVHFLNFITW
VKKDGFANAKKRYNHAQESILFYSMHKKNYTFNADEIRIAYESAERIKHAQSKGILKNN
KRWFPNPKGKLCLDVWEITSQRHVEKEKGKILKPKHPSIKPKALIERMIKASSHKNDLI
LDLFSGSGMTSLVAKSLERNFIGCESHAEYVHGSLEMFRYNECE

M1.*Hpy*AII (POC1484-KPL22), C-terminal His6-tag

MILNKIYIEDVFTFLDKLEDKSVDLAIIDPPYNLKIASWDSFKNDEEFLTFSYAWIDKM
LPKLKDTGSFYIFNTPFNCALFLAYLHHKKVHFLNFITWVKKDGFANAKKRYNHAQESI
LFYSMHKKNYTFNADEIRIAYESAERIKHAQSKGILKNNKRWFPNPKGKLCLDVWEITS
QRHVEKEKGKILKPKHPSIKPKALIERMIKASSHKNDLILDLFSGSGMTSLVAKSLERN
FIGCESHAEYVHGSLEMFRYNECEAAALEHHHHHH

M2.*Hpy*AII (POC1485-KPL23), N-terminal His6-tag

MGSSHHHHHHSSGLVPRGSHMNINKVFYHSSTNMNEVPDNSVDLIITSPPYFNIKDYAK
NGTQDLQHSAQHVEDLGALEKYEDYLLGLLKVWLECYRALKPNGKLCINVPLMPMLKKV
LNTHYNRHIFDLHADIQHSILHDLNNMLKNKPKMFLLDVYIWKRANPTKRLMFGSYPYP
RNFYAQNTIEFIGVFVKDGKPKQPTEEQKEQSQLTQEEWVEFTKQIWEIPIPNKNDIAF
GKHAALMPAELARRLIRLYSCVGDVVLDPFSGSGTTLREAKLLKRNFIGYELYENYKPL
IEQKLGNLFDFE

M2.*Hpy*AII (POC1486-KPL24), C-terminal His6-tag

MNINKVFYHSSTNMNEVPDNSVDLIITSPPYFNIKDYAKNGTQDLQHSAQHVEDLGALE
KYEDYLLGLLKVWLECYRALKPNGKLCINVPLMPMLKKVLNTHYNRHIFDLHADIQHSI
LHDLNNMLKNKPKMFLLDVYIWKRANPTKRLMFGSYPYPRNFYAQNTIEFIGVFVKDGK
PKQPTEEQKEQSQLTQEEWVEFTKQIWEIPIPNKNDIAFGKHAALMPAELARRLIRLYS
CVGDVVLDPFSGSGTTLREAKLLKRNFIGYELYENYKPLIEQKLGNLFDFEAAALEHHH
HHH

M1.*Mbo*II (POC1487-KPL25), N-terminal His6-tag

MGSSHHHHHHSSGLVPRGSHMVENMLEINKIHQMNCFDFLDQVENKSVQLAVIDPPYNL
SKADWDSFDSHNEFLAFTYRWIDKVLDKLDKDGSLYIFNTPFNCAFICQYLVSKGMIFQ
NWITWDKRDGMGSAKRRFSTGQETILFFSKSKNHTFNYDEVRVPYESTDRIKHASEKGI
LKNGKRWFPNPNGRLCGEVWHFSSQRHKEKVNGKTVKLTHITPKPRDLIERIIRASSNP
NDLVLDCFMGSGTTAIVAKKLGRNFIGCDMNAEYVNQANFVLNQLEIN

M1.*Mbo*II (POC1488-KPL26), C-terminal His6-tag

MVENMLEINKIHQMNCFDFLDQVENKSVQLAVIDPPYNLSKADWDSFDSHNEFLAFTYR
WIDKVLDKLDKDGSLYIFNTPFNCAFICQYLVSKGMIFQNWITWDKRDGMGSAKRRFST
GQETILFFSKSKNHTFNYDEVRVPYESTDRIKHASEKGILKNGKRWFPNPNGRLCGEVW
HFSSQRHKEKVNGKTVKLTHITPKPRDLIERIIRASSNPNDLVLDCFMGSGTTAIVAKK
LGRNFIGCDMNAEYVNQANFVLNQLEINKLAAALEHHHHHH

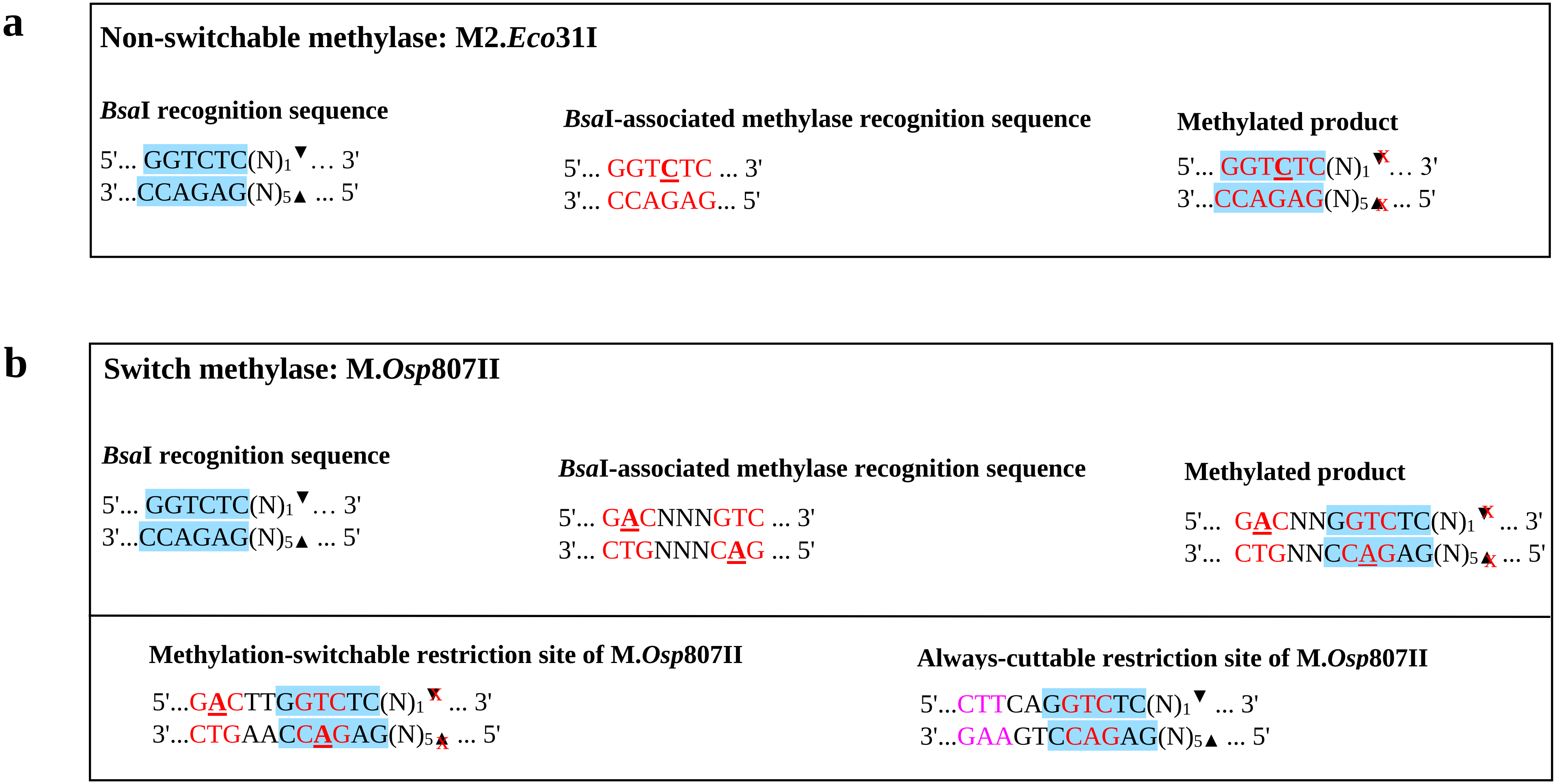

Fig. S1 (a) Representation of a non-switchable methylase, and **(b)** a switch methylase with their methylation-switchable and always-cuttable restriction sites. The type IIS recognition sequence is highlighted in light blue, the type IIS restriction site is marked with ▼▲, the methylase recognition sequence is in red, the methylated base is in bold and underlined, altered bases to prevent methylase activity are in pink and the blocked restriction site is marked with a red x

PCR amplification of methylases

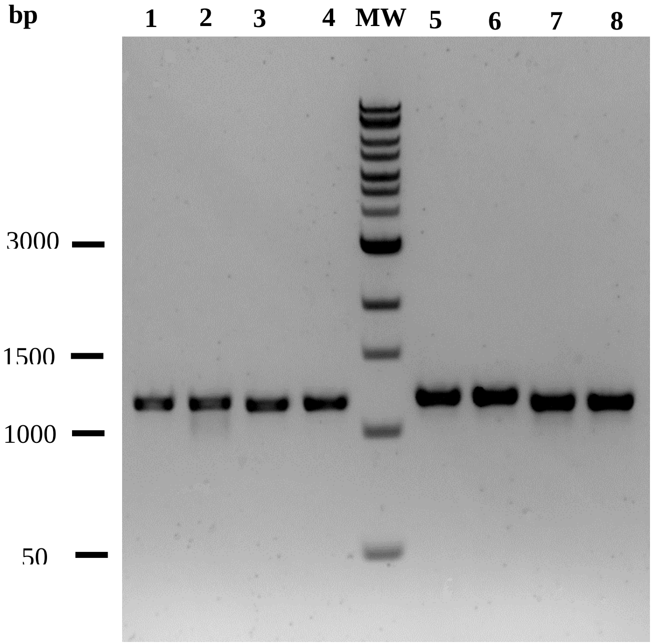

Fig. S2 Agarose gel electrophoresis (1%) showing the PCR products of M2.*Eco*31I and M2.*Bsa*I and their truncated versions. Lanes: 1, KPL01; 2, KLP02; 3, KPL04; 4, KPL04; 5, KPL05; 6, KLP06; 7, KPL07; and 8, KPL08. MW, molecular weight marker (Quick-Load® 1 kb Extend DNA Ladder, New England Biolabs, N3239S, 0.5 to 48.5 kb)

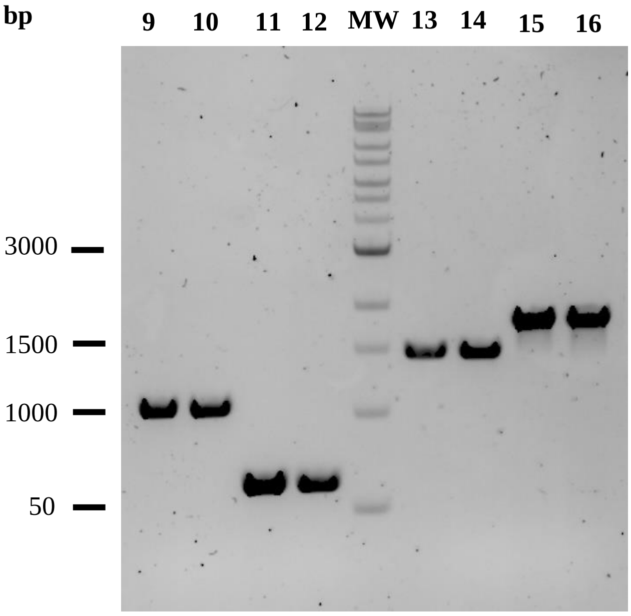

Fig. S3 Agarose gel electrophoresis (1%) showing the PCR products of M.*Osp*807II, M2.*Nme*MC58II, M.*Sen*0738I, and S.*Sen*0738I. Lanes: 9, KPL09; 10, KLP10; 11, KPL11; 12, KPL12; 13, KPL13; 14, KLP14; 15, KPL15; and 16, KPL16. MW, molecular weight marker (Quick-Load® 1 kb Extend DNA Ladder, New England Biolabs, N3239S, 0.5 to 48.5 kb)

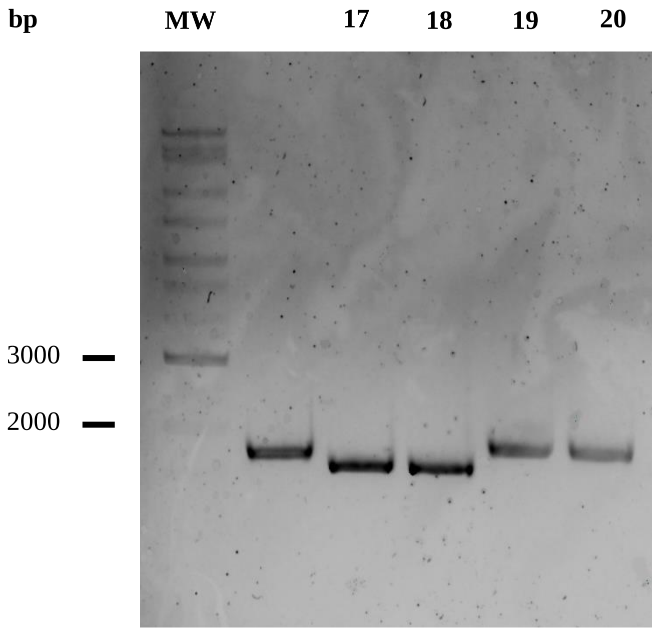

Fig. S4 Agarose gel electrophoresis (1%) showing the PCR products of M1.*Eco*31I and M.*Xmn*I. Lanes: 17, KPL17; 18, KLP18; 19, KPL19; and 20, KPL20. MW, molecular weight marker (Quick-Load® 1 kb Extend DNA Ladder, New England Biolabs, N3239S, 0.5 to 48.5 kb)

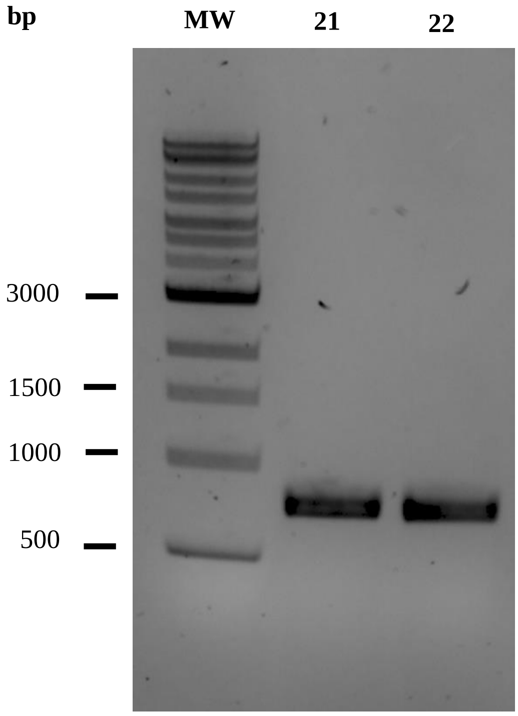

Fig. S5 Agarose gel electrophoresis (1%) showing the PCR products of M1.*Hpy*AII. Lanes: 21, KPL21; and 22, KLP22. MW, molecular weight marker (Quick-Load® 1 kb Extend DNA Ladder, New England Biolabs, N3239S, 0.5 to 48.5 kb)

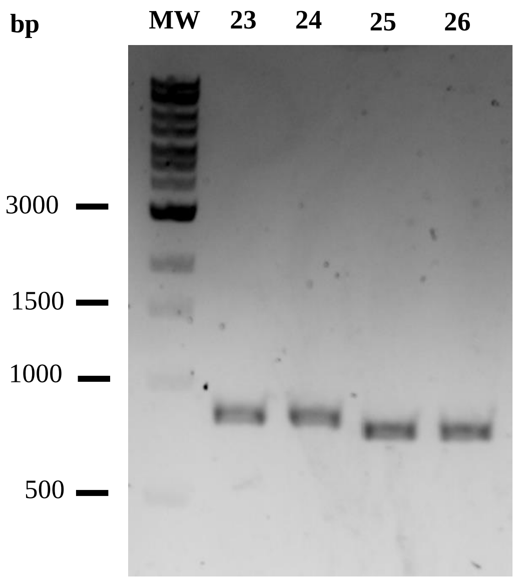

Fig. S6 Agarose gel electrophoresis (1%) showing the PCR products of M2.*Hpy*AII and M1.*Mbo*II. Lanes: 23, KPL23; 24, KLP24; 25, KPL25; and 26, KPL26. MW, molecular weight marker (Quick-Load® 1 kb Extend DNA Ladder, New England Biolabs, N3239S, 0.5 to 48.5 kb)

Protein expression of methylases

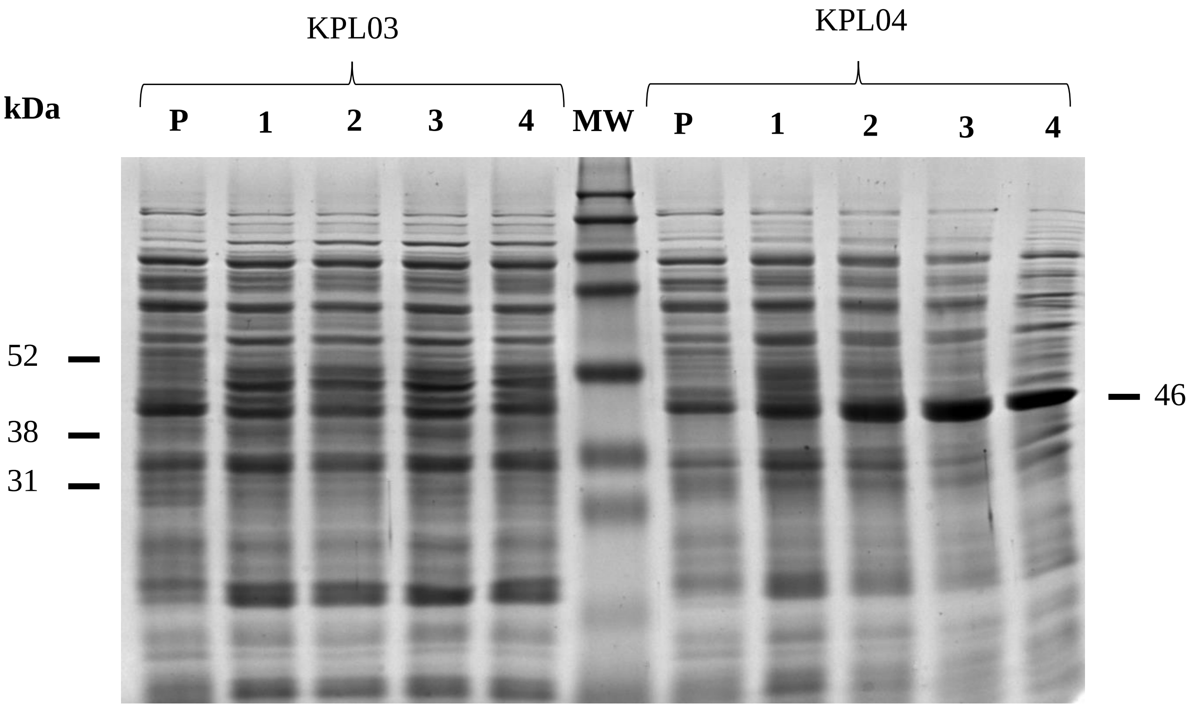

Fig. S7 SDS-PAGE showing the expression of M2.*Eco*31I_2 (KPL03 and KPL04) at different IPTG concentrations. Lanes: P, pre-induction; 1, 0.005 mM; 2, 0.05 mM; 3, 0.5 mM; and 4, 1 mM. MW, molecular weight marker (Amersham™ ECL™ Rainbow™ Marker - Full range, Full Range, Cytiva RPN800E, 12 to 225 kDa)

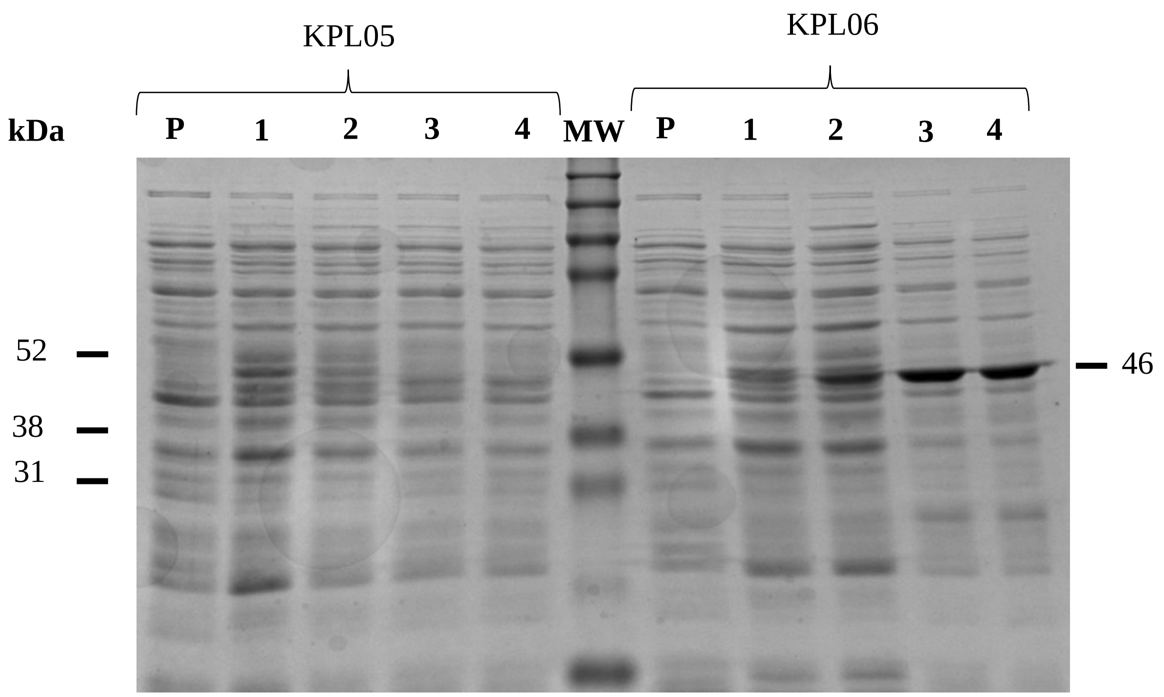

Fig. S8 SDS-PAGE showing the expression of M2.*Bsa*I (KPL05 and KPL06) at different IPTG concentrations. Lanes: P, pre-induction; 1, 0.005 mM; 2, 0.05 mM; 3, 0.5 mM; and 4, 1 mM. MW, molecular weight marker (Amersham™ ECL™ Rainbow™ Marker - Full range, Full Range, Cytiva RPN800E, 12 to 225 kDa)

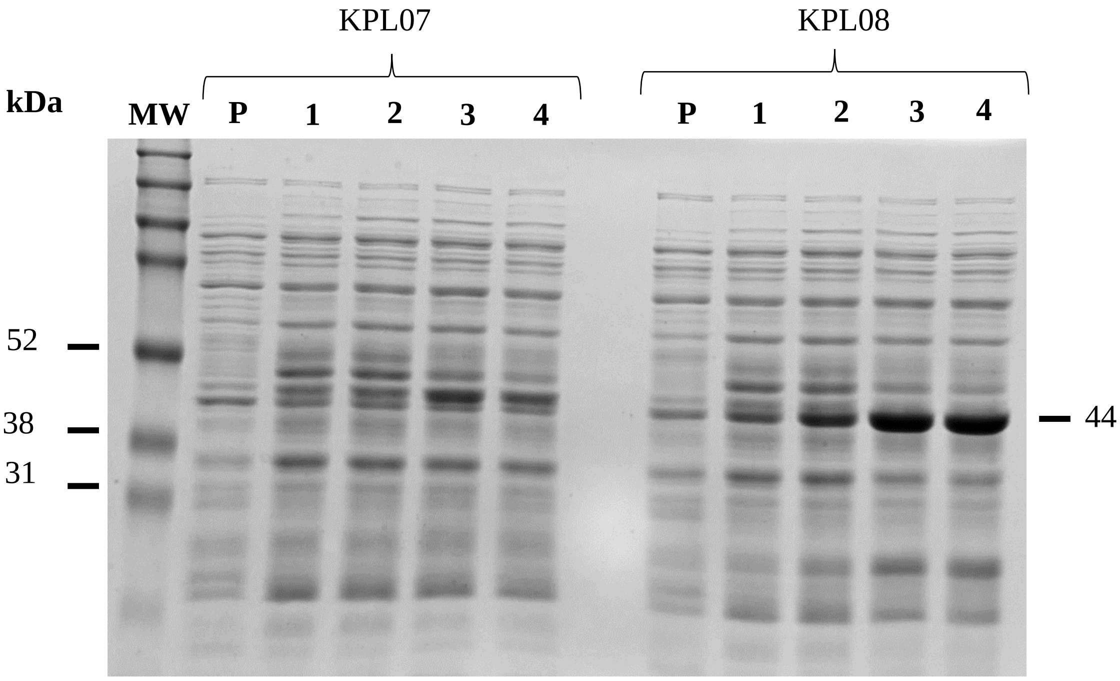

Fig. S9 SDS-PAGE showing the expression of M2.*Bsa*I_2 (KPL07 and KPL08) at different IPTG concentrations. Lanes: P, pre-induction; 1, 0.005 mM; 2, 0.05 mM; 3, 0.5 mM; and 4, 1 mM. MW, molecular weight marker (Amersham™ ECL™ Rainbow™ Marker - Full range, Full Range, Cytiva RPN800E, 12 to 225 kDa)

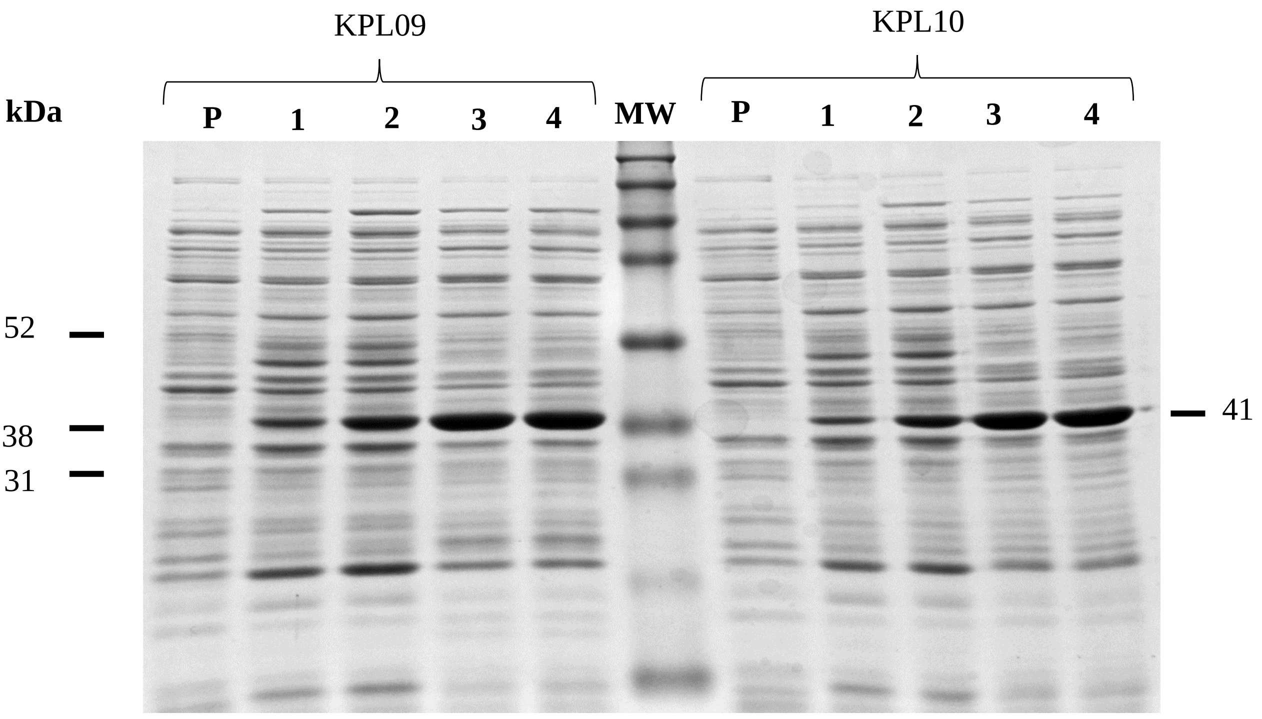

Fig. S10 SDS-PAGE showing the expression of M.*Osp*807II (KPL09 and KPL10) at different IPTG concentrations. Lanes: P, pre-induction; 1, 0.005 mM; 2, 0.05 mM; 3, 0.5 mM; and 4, 1 mM. MW, molecular weight marker (Amersham™ ECL™ Rainbow™ Marker - Full range, Full Range, Cytiva RPN800E, 12 to 225 kDa)

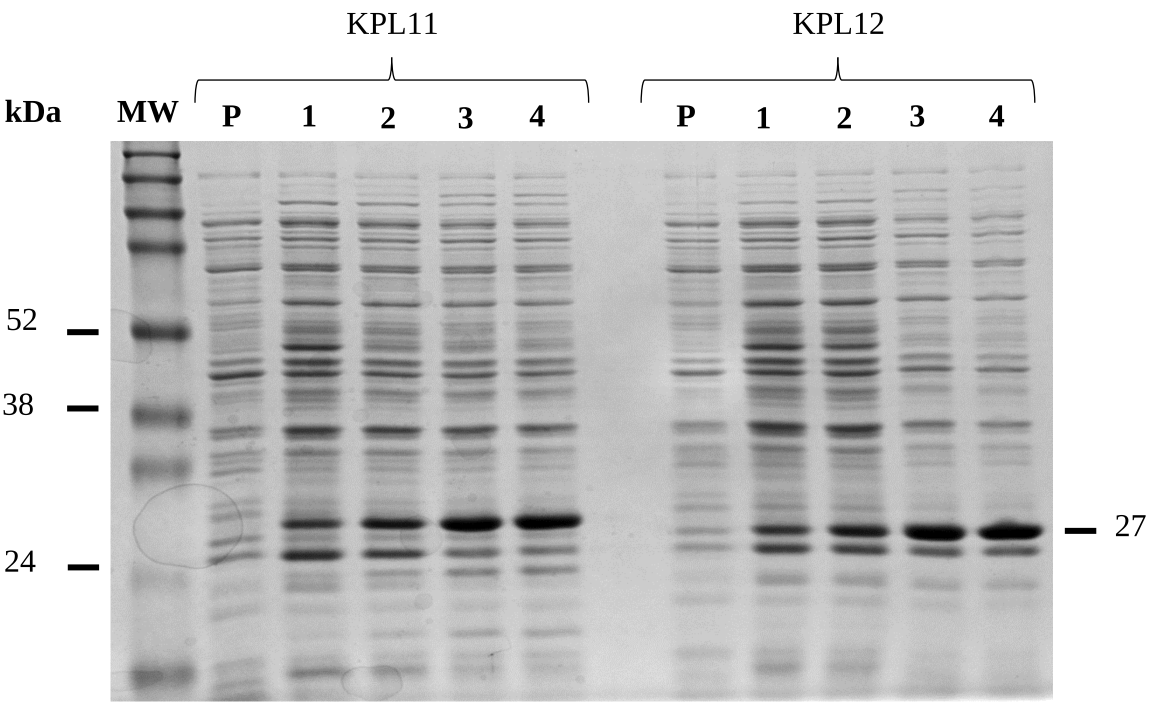

Fig. S11 SDS-PAGE showing the expression of M2.*Nme*MC58II (KPL11 and KPL12) at different IPTG concentrations. Lanes: P, pre-induction; 1, 0.005 mM; 2, 0.05 mM; 3, 0.5 mM; and 4, 1 mM. MW, molecular weight marker (Amersham™ ECL™ Rainbow™ Marker - Full range, Full Range, Cytiva RPN800E, 12 to 225 kDa)

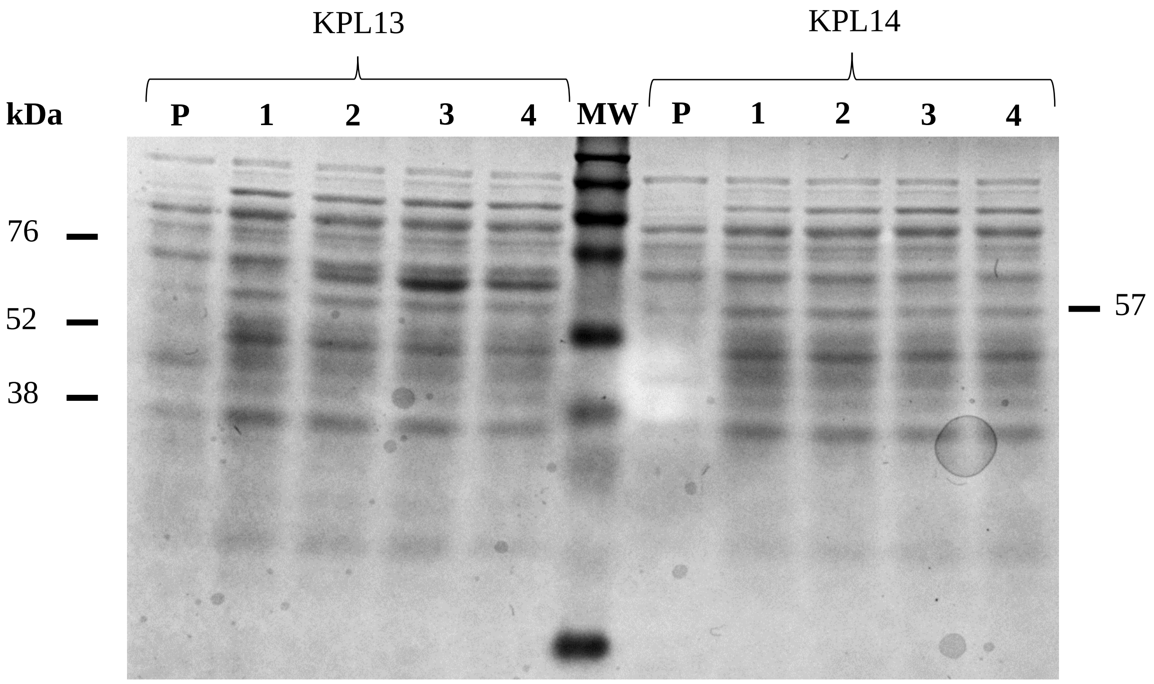

Fig. S12 SDS-PAGE showing the expression of M.*Sen*0738I (KPL13 and KPL14) at different IPTG concentrations. Lanes: P, pre-induction; 1, 0.005 mM; 2, 0.05 mM; 3, 0.5 mM; and 4, 1 mM. MW, molecular weight marker (Amersham™ ECL™ Rainbow™ Marker - Full range, Full Range, Cytiva RPN800E, 12 to 225 kDa)

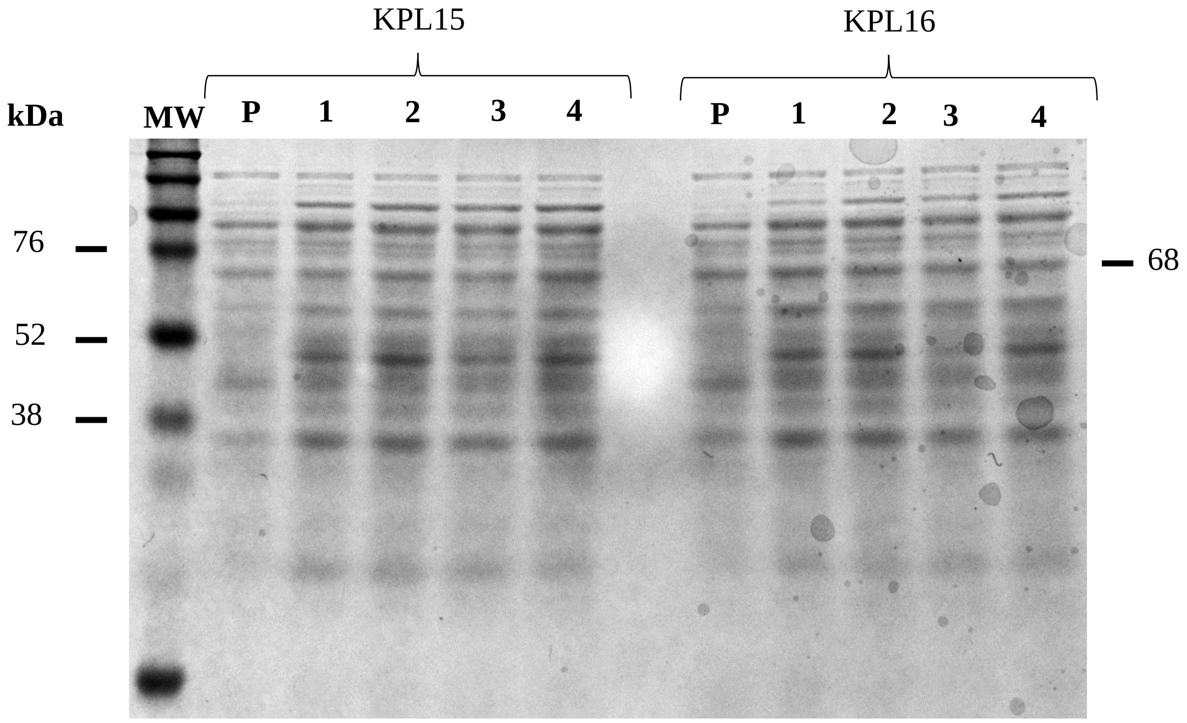

Fig. S13 SDS-PAGE showing the expression of S.*Sen*0738I (KPL15 and KPL16) at different IPTG concentrations. Lanes: P, pre-induction; 1, 0.005 mM; 2, 0.05 mM; 3, 0.5 mM; and 4, 1 mM. MW, molecular weight marker (Amersham™ ECL™ Rainbow™ Marker - Full range, Full Range, Cytiva RPN800E, 12 to 225 kDa)

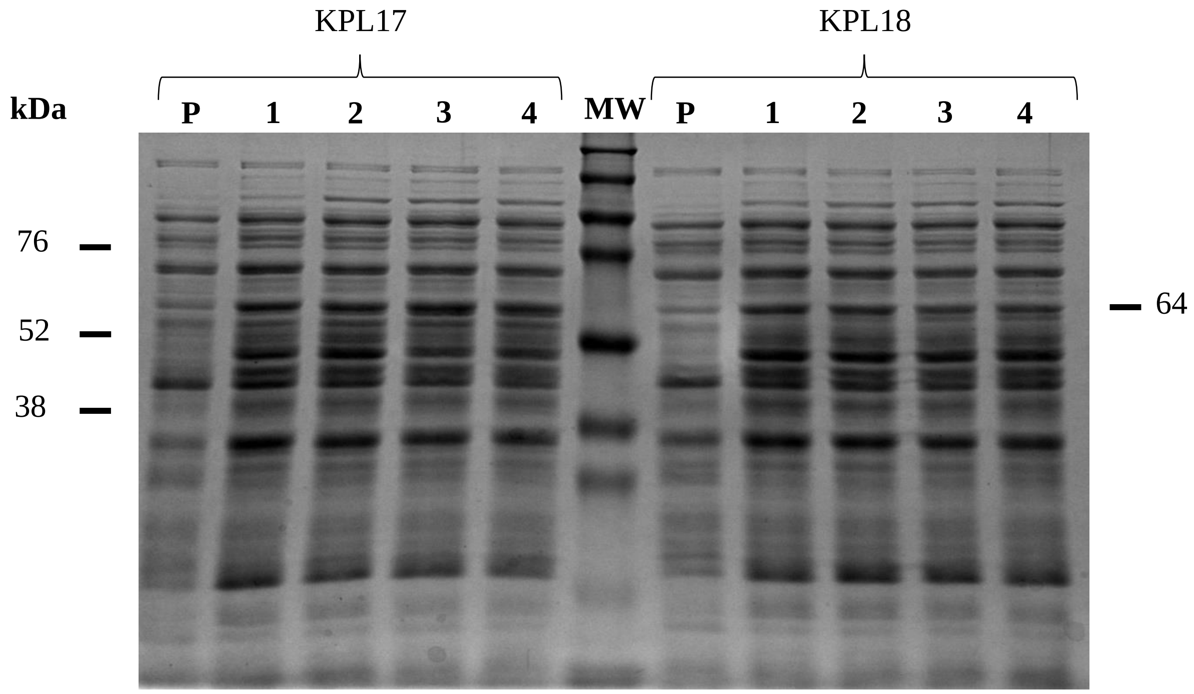

Fig. S14 SDS-PAGE showing the expression of M1.*Eco*31I (KPL17 and KPL18) at different IPTG concentrations. Lanes: P, pre-induction; 1, 0.005 mM; 2, 0.05 mM; 3, 0.5 mM; and 4, 1 mM. MW, molecular weight marker (Amersham™ ECL™ Rainbow™ Marker - Full range, Full Range, Cytiva RPN800E, 12 to 225 kDa)

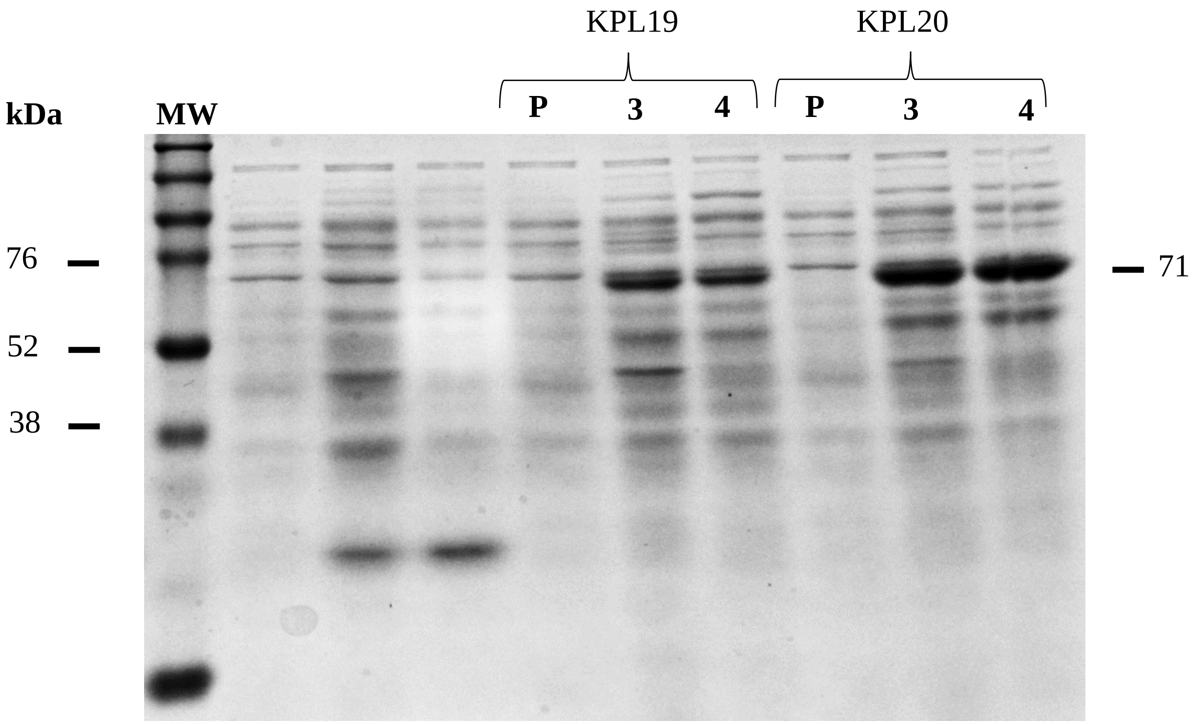

Fig. S15 SDS-PAGE showing the expression of M.*Xmn*I (KPL19 and KPL20) at different IPTG concentrations. Lanes: P, pre-induction; 3, 0.5 mM; and 4, 1 mM. MW, molecular weight marker (Amersham™ ECL™ Rainbow™ Marker - Full range, Full Range, Cytiva RPN800E, 12 to 225 kDa)

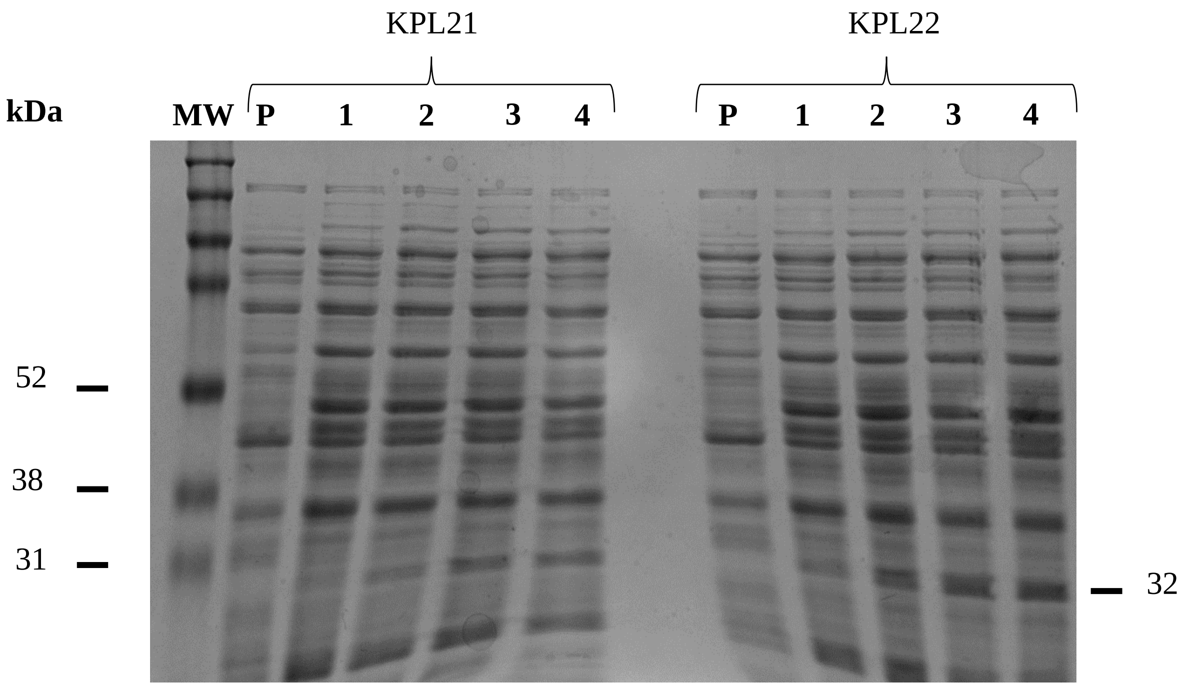

Fig. S16 SDS-PAGE showing the expression of M1.*Hpy*AII (KPL21 and KPL22) at different IPTG concentrations. Lanes: P, pre-induction; 1, 0.005 mM; 2, 0.05 mM; 3, 0.5 mM; and 4, 1 mM. MW, molecular weight marker (Amersham™ ECL™ Rainbow™ Marker - Full range, Full Range, Cytiva RPN800E, 12 to 225 kDa)

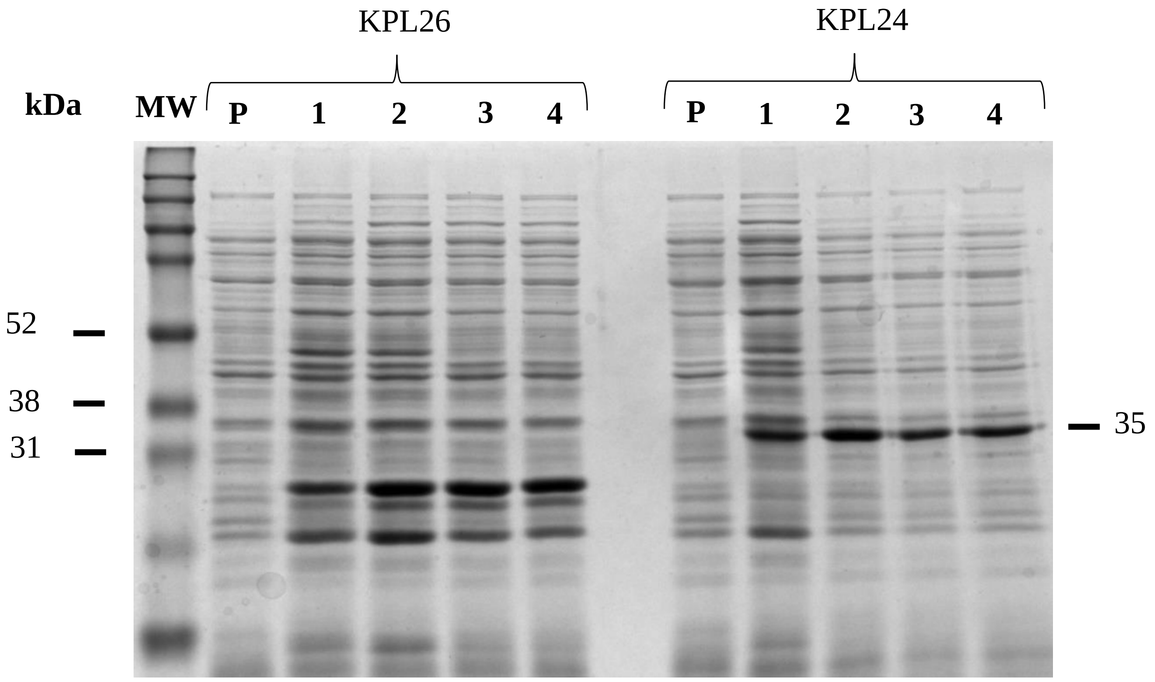

Fig. S17 SDS-PAGE showing the expression of M2.*Hpy*AII (KPL24) and M1.*Mbo*II (KPL26) at different IPTG concentrations. Lanes: P, pre-induction; 1, 0.005 mM; 2, 0.05 mM; 3, 0.5 mM; and 4, 1 mM. MW, molecular weight marker (Amersham™ ECL™ Rainbow™ Marker - Full range, Full Range, Cytiva RPN800E, 12 to 225 kDa)

Analysis of DNA methylation

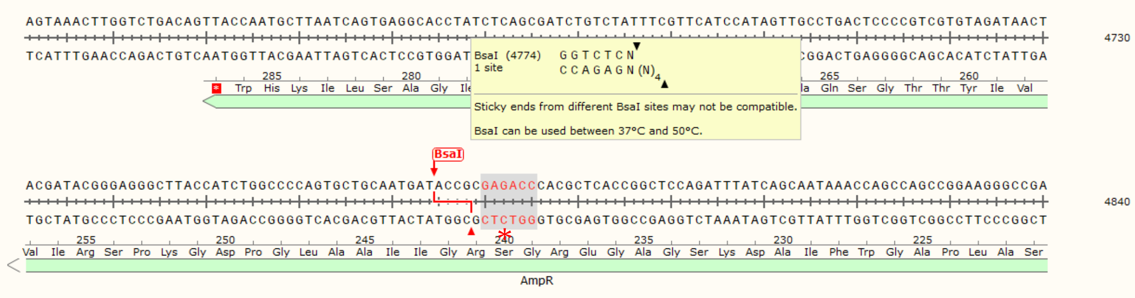

Fig. S18 pET15b fragment for testing the activity of M2.*Eco*31I, M1.*Eco*31I, and M2.*Bsa*I *Bsa*I-associated non-switchable methylases. The recognition sequence of the methylase is in red with the methylated base (C) for M2.*Eco*31I marked with a red asterisk (*). The *Bsa*I recognition sequence is in the reverse direction

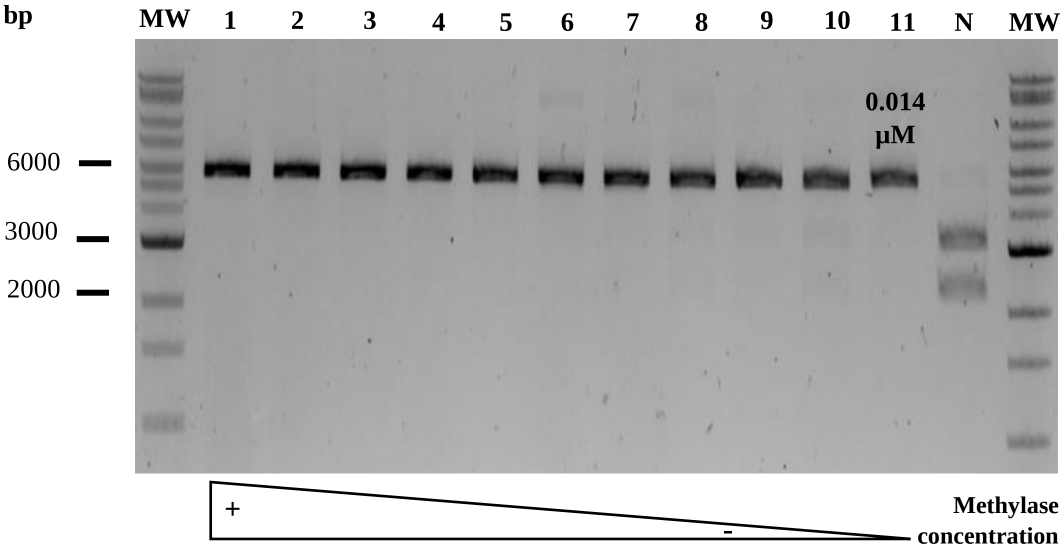

Fig. S19 Agarose gel electrophoresis (1%) showing the activity of M2.*Eco*31I_2 (KPL04) *Bsa*I-associated non-switchable methylase on pET15b at different protein concentrations. Lanes:1 to 11, 2-fold serial dilutions from 14 to 0.014 µM; and N, negative control (without methylase). MW, molecular weight marker (Quick-Load® 1 kb Extend DNA Ladder, New England Biolabs, N3239S, 0.5 to 48.5 kb)

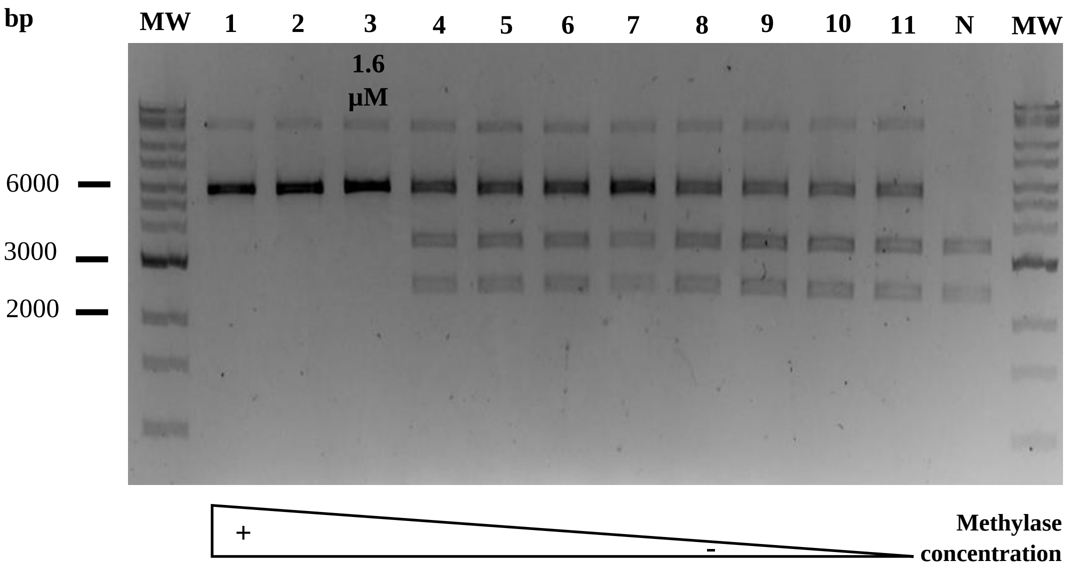

Fig. S20 Agarose gel electrophoresis (1%) showing the activity of M2.*Bsa*I (KPL06) *Bsa*I-associated non-switchable methylase on pET15b at different protein concentrations. Lanes:1 to 11, 2-fold serial dilutions from 6.3 to 0.006 µM; and N, negative control (without methylase). MW, molecular weight marker (Quick-Load® 1 kb Extend DNA Ladder, New England Biolabs, N3239S, 0.5 to 48.5 kb)

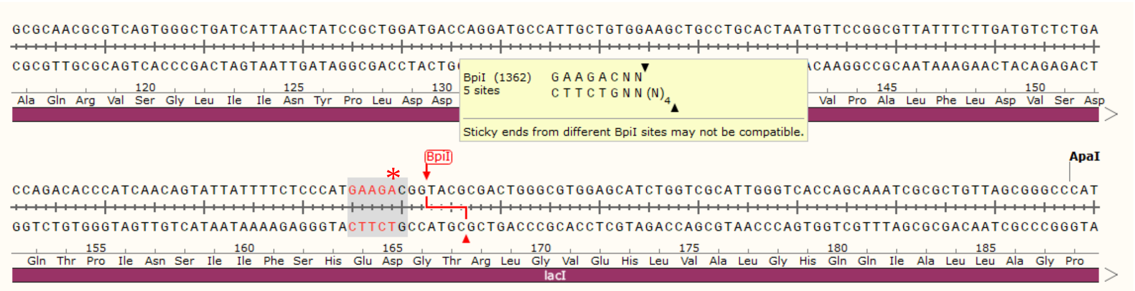

Fig. S21 pET15b fragment for testing the activity of M1.*Hpy*AII, M2.*Hpy*AII, and M1.*Mbo*II *Bpi*I-associated non-switchable methylases. The recognition sequence of the methylase is in red with the methylated base (A) for M1.*Hpy*AII marked with a red asterisk (*)

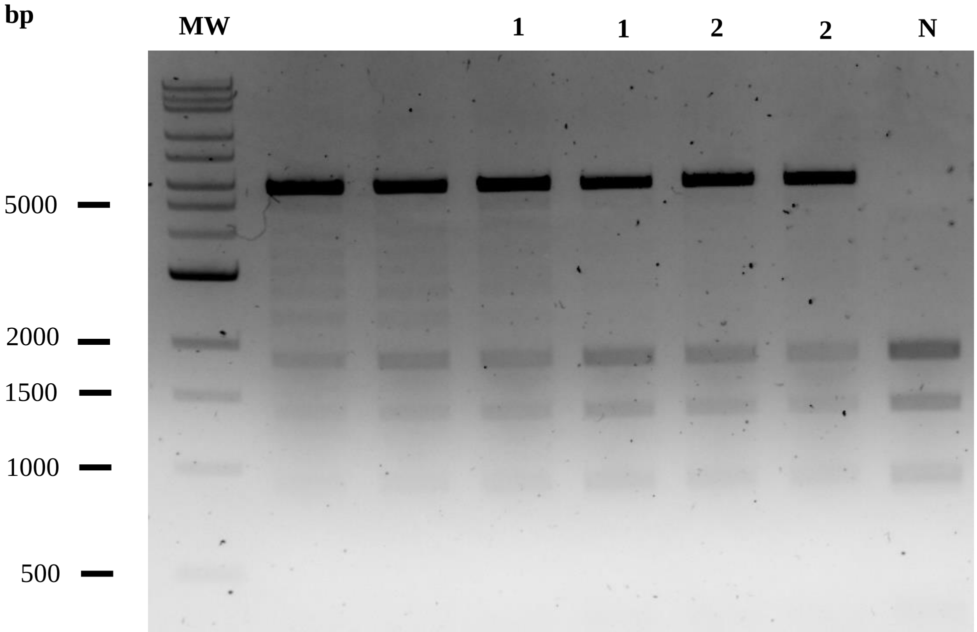

Fig. S22 Agarose gel electrophoresis (1%) showing the activity of M1.*Hpy*AII (KPL21 and KPL22) *Bpi*I-associated non-switchable methylase on pET15b. Lanes: 1, KPL21 (1.17 µM); 2, KPL22 (1.96 µM); and N, negative control (without methylase). MW, molecular weight marker (Quick-Load® 1 kb Extend DNA Ladder, New England Biolabs, N3239S, 0.5 to 48.5 kb). Bands less than 500 bp are not observed in the gel

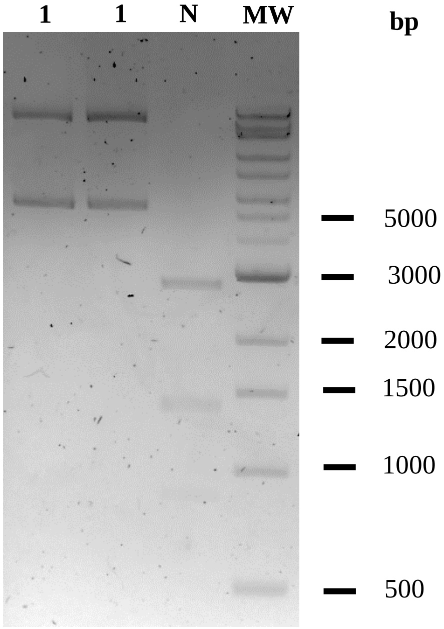

Fig. S23 Agarose gel electrophoresis (1%) showing the activity of M1.*Mbo*II (KPL26) *Bpi*I-associated non-switchable methylase on pET15b. Lanes: 1, KPL26 (15 µM); and N, negative control (without methylase). MW, molecular weight marker (Quick-Load® 1 kb Extend DNA Ladder, New England Biolabs, N3239S, 0.5 to 48.5 kb). The reactions were digested only with *Bpi*I. The methylated plasmid exhibits a band corresponding to the undigested plasmid (5708 bp) and a band corresponding to nicked plasmid DNA. The non-methylated plasmid exhibits 5 bands (339, 374, 863, 1379, and 2753 bp) as a result of the digestion of *Bpi*I. Bands less than 500 bp are not observed in the gel

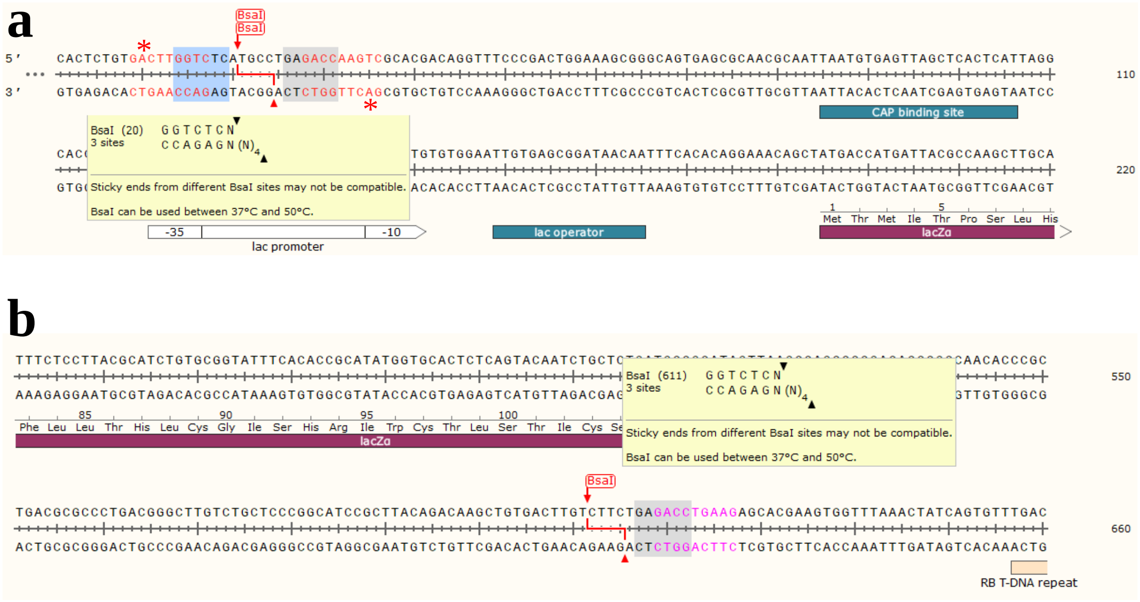

Fig. S24 POC1399 for testing the activity of M.*Osp*807II *Bsa*I-associated switch methylase. **(A)** *Bsa*I sites at position 20 (methylation-switchable restriction site) where the recognition sequence of the methylase is in red and the methylated base (A: adenine) is marked with a red asterisk (*). **(B)** *Bsa*I site at position 611 (always-cuttable restriction site) where the recognition sequence of the methylase has been altered such that it is no longer recognised by the methylase and so is not functional (in magenta). The second and third *Bsa*I recognition sequences are in the reverse direction

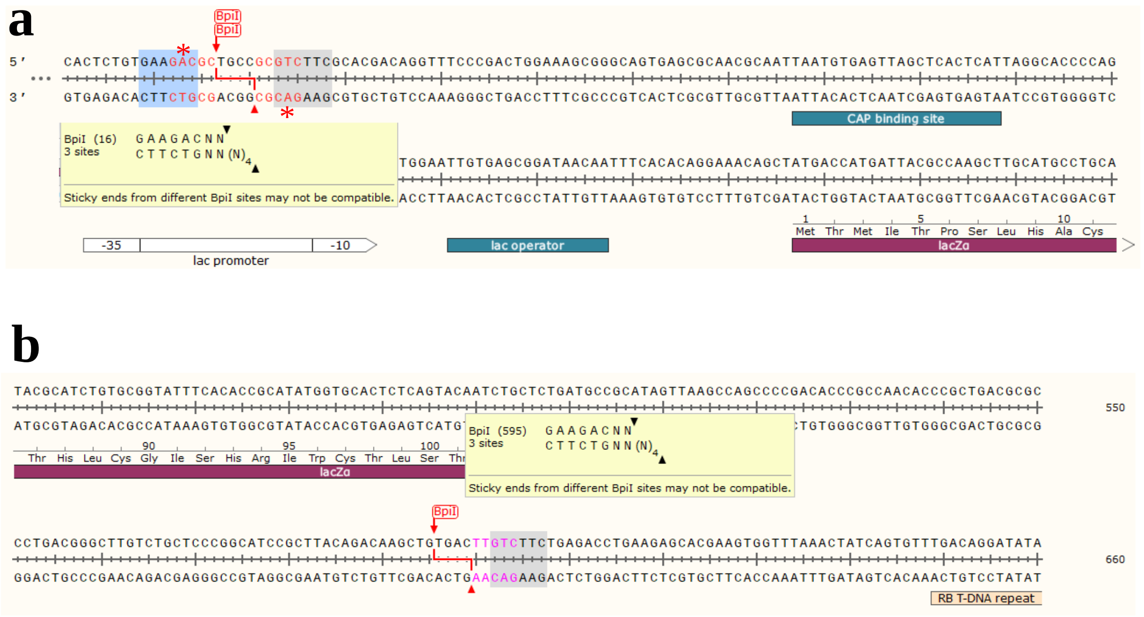

Fig. S25 POC1400 for testing the activity of M2.*Nme*MC58II *Bpi*I-associated switch methylase. **(a)** *Bpi*I sites at position 16 (methylation-switchable restriction site) where the recognition sequence of the methylase is in red and the methylated base (A: adenine) is marked with a red asterisk (*). **(b)** *Bpi*I site at position 595 (always-cuttable restriction site) where the recognition sequence of the methylase has been altered such that it is no longer recognised by the methylase and so is not functional (in magenta). The second and third *Bpi*I recognition sequences are in the reverse direction

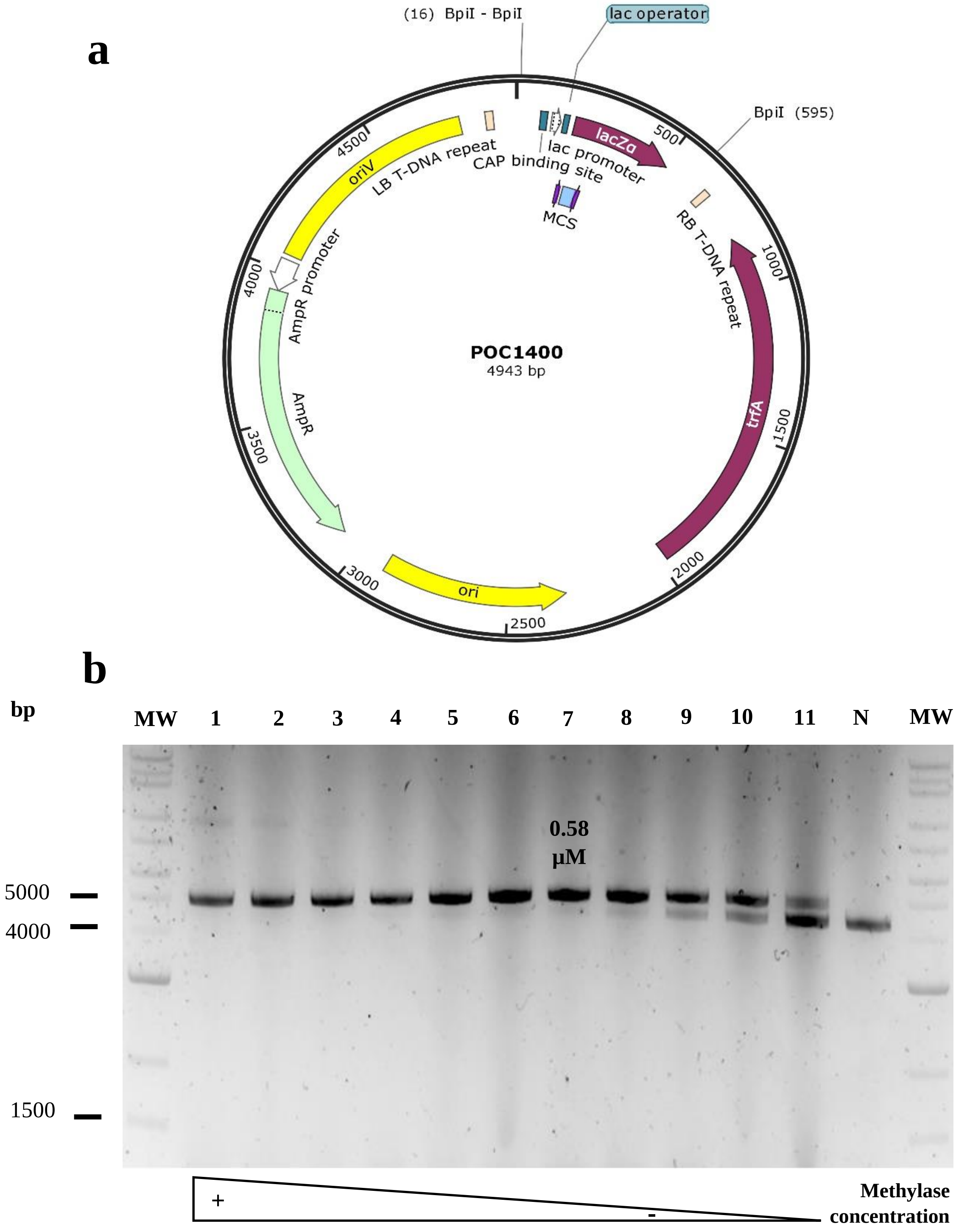

Fig. S26 Activity of *Bpi*I-associated switch methylases on POC1400. **(a)** POC1400 showing the *Bpi*I restriction sites for the activity test (Supplementary Figure S24). The methylated plasmid will exhibit a band on the gel (4943 bp) corresponding to the linearised plasmid digested by *Bpi*I at position 595. The non-methylated plasmid will exhibit two bands (579 and 4364 bp) as a result of *Bpi*I digestion at positions 16 and 595. **(b)** Agarose gel electrophoresis (1%) showing the activity of M2.*Nme*MC58II (KPL11) at different protein concentrations. Lanes: 1 to 11, 2-fold serial dilutions from 37.5 to 0.04 µM; and N, negative control (without methylase). MW, molecular weight marker (Quick-Load® 1 kb Extend DNA Ladder, New England Biolabs, N3239S, 0.5 to 48.5 kb). Bands less than 1500 bp are not observed in the gel

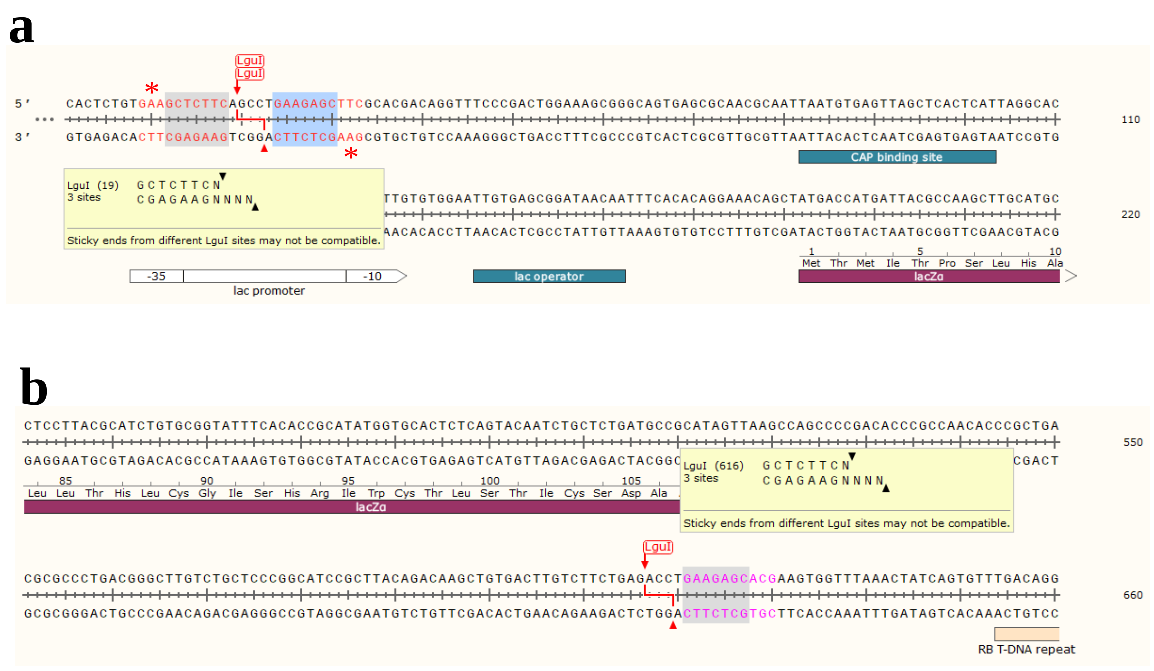

Fig. S27 POC1401 for testing the activity of M.*Xmn*I *Lgu*I-associated switch methylase. **(a)** *Lgu*I sites at position 19 (methylation-switchable restriction site) where the recognition sequence of the methylase is in red and the methylated base (A: adenine) is marked with a red asterisk (*). **(b)** *Lgu*I site at position 616 (always-cuttable restriction site) where the recognition sequence of the methylase has been altered such that it is no longer recognised by the methylase and so is not functional (in magenta). The second and third *Lgu*I recognition sequences are in the reverse direction

a

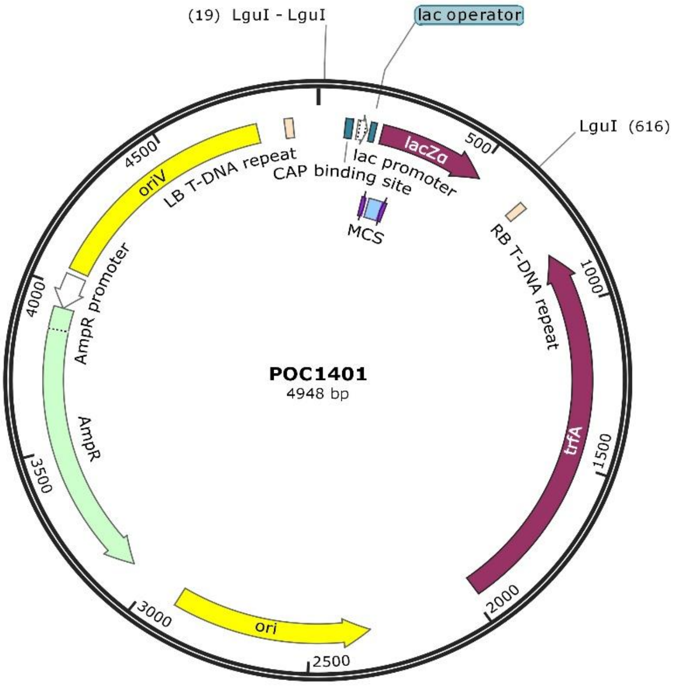

b

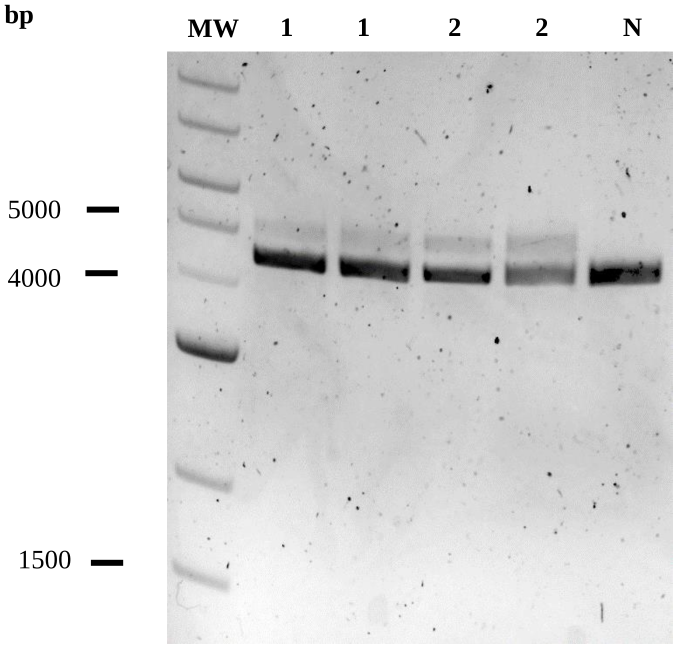

Fig. S28 Activity of *Lgu*I-associated switch methylases on POC1401. **(a)** POC1401 showing the *Lgu*I restriction sites for the activity test (Supplementary Figure S26). The methylated plasmid will exhibit a band on the gel (4948 bp) corresponding to the linearised plasmid digested by *Lgu*I at position 616. The non-methylated plasmid will exhibit two bands (597 and 4351 bp) as a result of *Lgu*I digestion at positions 19 and 616. **(b)** Agarose gel electrophoresis (1%) showing the activity of M.*Xmn*I (KPL19 and KPL20). Lanes: 1, KLP19 at 0.94 µM; 2; KPL20 at 2.2 µM; and N, negative control (without methylase). MW, molecular weight marker (Quick-Load® 1 kb Extend DNA Ladder, New England Biolabs, N3239S, 0.5 to 48.5 kb). Bands less than 1500 bp are not observed in the gel

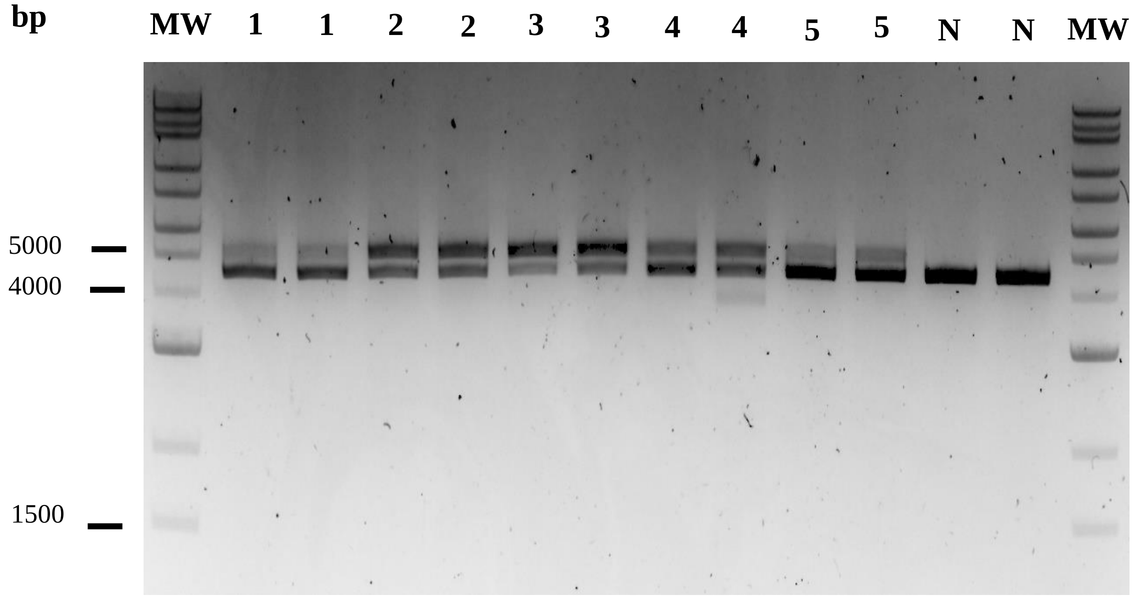

Fig. S29 Effect of pH on the activity of M.*Xmn*I (KPL20) *Lgu*I-associated switch methylase on POC1401. The methylated plasmid exhibits a band on the gel (4948 bp) corresponding to the linearised plasmid digested by *Lgu*I at position 616. The non-methylated plasmid exhibits two bands (597 and 4351 bp) as a result of *Lgu*I digestion at positions 19 and 616. Lanes: 1, pH 6.9; 2, pH 7.5; 3, pH 7.9; 4, pH 8.4; 5, pH 8.8; and N, negative control (without methylase). MW, molecular weight marker (Quick-Load® 1 kb Extend DNA Ladder, New England Biolabs, N3239S, 0.5 to 48.5 kb). A concentration of 2.2 µM of methylase was used. Bands less than 1500 bp are not observed in the gel
